# Coordination-induced axial chirality controls the metal-centred configuration in a stereogenic-at-iron catalyst†

**DOI:** 10.1039/d4cc06227b

**Published:** 2025-01-08

**Authors:** Benedikt Buchberger, Nemrud Demirel, Xiulan Xie, Sergei I. Ivlev, Eric Meggers

**Affiliations:** a Fachbereich Chemie, Philipps-Universität Marburg Hans-Meerwein-Strasse 4 35043 Marburg Germany meggers@chemie.uni-marburg.de

## Abstract

A new approach is introduced to control the metal-centred configuration of stereogenic-at-iron catalysts by utilizing axial ligand chirality, which becomes locked upon metal coordination. This strategy is applied to an iron catalyst containing two chelating *N*-(2-pyridyl)-substituted triazol-5-ylidene mesoionic carbenes (MICs) resulting in a helical topology with a stereogenic iron centre.

## Abstract 2

A new approach is introduced to control the metal-centred configuration of stereogenic-at-iron catalysts by utilizing axial ligand chirality, which becomes locked upon metal coordination.

## Graphical abstract

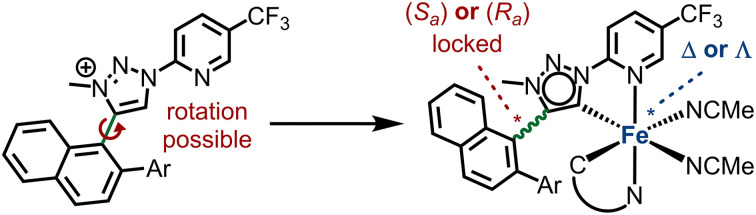

Chiral transition metal complexes play a crucial role in asymmetric catalysis.^1^ Traditionally, these catalysts are developed by combining chiral ligands with metal precursors, a strategy that has dominated for decades. In contrast, a recently emerging chiral-at-metal approach utilizes metal-centred chirality, with achiral ligands arranged around a stereogenic metal centre.^2^ This concept has been successfully applied to the noble metals iridium(iii),^3^ rhodium(iii),^4^ and ruthenium(ii).^5^ More recently, our focus has shifted to 3d metals, which are appealing for sustainable catalysis due to their abundance and lower toxicity. We reported the first example of a chiral-at-iron catalyst, in which chirality is generated exclusively by achiral ligands.^6^ In this catalyst, two bidentate *N*-(2-pyridyl)-substituted N-heterocyclic carbenes (PyNHC) chelate iron(ii) to induce a helical topology with a stereogenic iron centre. Two additional acetonitrile ligands, positioned *trans* to the σ-donating NHC ligands, create labile coordination sites that facilitate substrate binding and catalysis. While this catalyst demonstrated promising catalytic activity in asymmetric Cannizzaro reactions, a Nazarov cyclisation, and hetero-Diels–Alder reactions, some racemisation occurred over time.^6,7^ To address this, we have explored various scaffold modifications aimed at improving configurational stability and expanding the reactivity of the iron catalysts (Fig. 1a). Replacing the imidazol-2-ylidene NHC ligand with a more π-accepting benzimidazol-2-ylidene improved configurational stability, but we were unable to demonstrate catalytic activity beyond Lewis acid catalysis.^8^ To enhance reactivity, we introduced a stronger σ-donor, 1,2,3-triazol-5-ylidene, which enabled nitrene-mediated intramolecular C(sp^3^)–H amination.^9^ However, this scaffold suffered from significant configurational lability. For compensation, we incorporated chirality into the ligand framework by using a chiral pinene-modified pyridyl ligand. However, this had the drawback of providing reduced asymmetric induction (Fig. 1b).

**Fig. 1:**
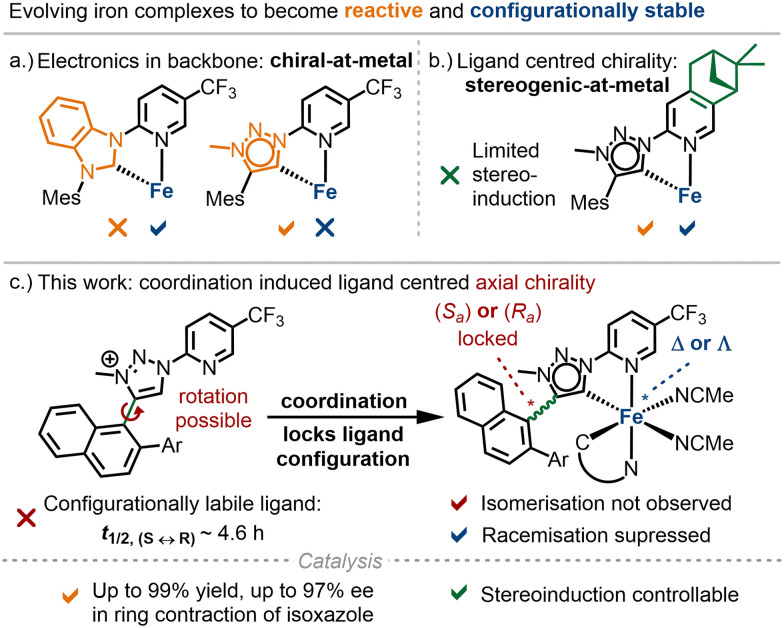
Previous work and this study to control the stereogenic iron centre in chiral iron catalysts.

Herein, we present a new strategy to increase configurational stability of chiral-at-iron catalysts by exploiting a locked axial chirality in the ligands upon metal coordination (Fig. 1c).

We selected the naphthyl triazolium ligands 1 and 2 (Scheme 1), which inherently feature axial chirality but undergo rapid racemisation (half-life of 4.6 hours at room temperature for 1).^10^ We hypothesised that converting these bidentate ligands into iron-coordinated mesoionic carbenes (MICs),^11^ specifically by replacing the C–H bond at the 5-position of the triazolium ion with a C–Fe bond, would restrict rotation due to the sterically demanding coordination sphere around the iron. This would effectively freeze the induced axial chirality and promote a matched and stable metal-centred configuration.

**Scheme 1:**
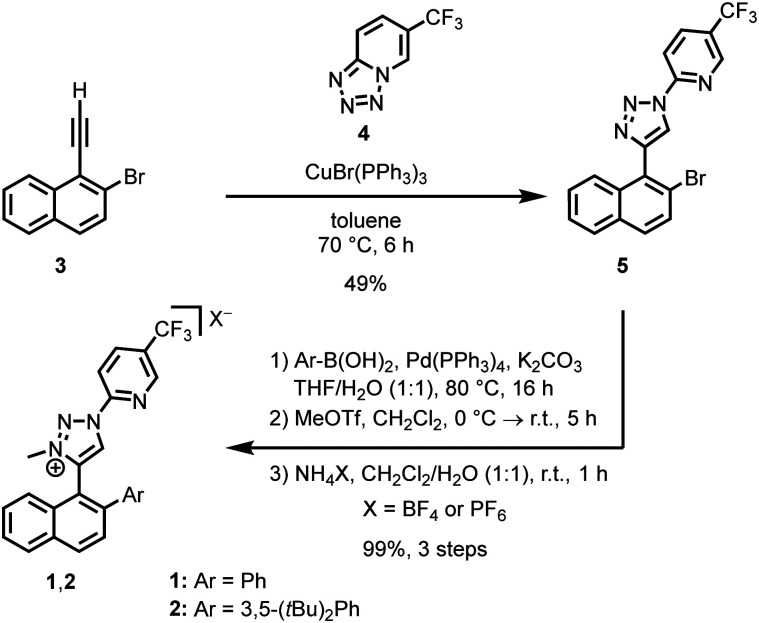
Synthesis of ligands 1 and 2 from the alkyne 3.

The ligand synthesis started with the alkyne 3. A CuAAC reaction with tetrazole 4 at 70 °C in toluene afforded the 1,4-disubstituted triazole 5 (49%).^12^ This was followed by a Suzuki coupling with an aryl boronic acid to introduce the desired aryl moiety (Ar = Ph or 3,5-(*t*Bu)_2_Ph), a methylation with MeOTf, and subsequent anion exchange with NH_4_PF_6_ or NH_4_BF_4_ to provide ligands 1 (Ar = Ph) and 2 (Ar = 3,5-(*t*Bu)_2_Ph) in 99% yield over three steps.

Next, with the ligands in hand, we synthesised racemic iron(ii) complexes by first generating silver carbenes of the triazolium ligands using Ag_2_O, followed by transmetalation with FeCl_2_ (Table 1).^13^ Interestingly, two diastereomers formed, one with *C*_2_-symmetry and one with *C*_1_-symmetry: *rac*-Fe1-C_2_ and *rac*-Fe1-C_1_ for Ar = Ph, as well as *rac*-Fe2-C_2_ and *rac*-Fe2-C_1_ for Ar = 3,5-(*t*Bu)_2_Ph. We found that the reaction conditions significantly affect the ratio. For example, conducting the transmetalation at room temperature for 2.5 hours, followed by the addition of 10 equiv. of NH_4_PF_6_, favoured the formation of the *C*_1_-symmetric isomers (entries 1 and 2). In contrast, when the transmetalation was performed in the presence of 2.5 equiv. of AgBF_4_, the diastereomeric ratio shifted toward the *C*_2_-symmetric diastereomer for Ar = 3,5-(*t*Bu)_2_Ph (entry 3), while for Ar = Ph, both diastereomers were generated in equal amounts (entry 4).

**Table 1:** Reaction conditions for the synthesis of iron complexes^a^

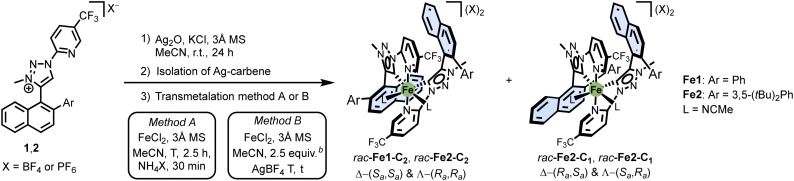								
Entry	Method	Ar	*T* (°C)	*t* (h)	Isomer-ratio^c^		Yield^d^ (%)	X
					*C*_2_	*C*_1_		
1	A	Ph	r.t.	3	1	2.4	91	PF_6_
2	A	3,5-(*t*Bu)_2_Ph	r.t.	3	1	2.2	66	PF_6_
3	B	3,5-(*t*Bu)_2_Ph	−40 °C → r.t.^e^	70^e^	7	1	64^f^	BF_4_
4	B	Ph	0 °C → r.t.^e^	17^e^	1	1	78^f^	BF_4_

a The depiction of the complexes only features Δ-enantiomers; a representation of the possible isomers and their relation can be found in the ESI.

b Referred to the intermediate monocationic Ag–carbene dimer.

c Ratio determined by ^1^H and ^19^F NMR after short workup.

d Yields determined from crude NMR and absolute weight of the crude after short workup. Free ligand was calculated out by factoring the crude NMR ratios and the respective molecular masses.

e See ESI for a detailed gradual warming protocol.

f Yield after silica column chromatography to ensure full removal of unreacted traces of the Ag–carbene intermediate.

Fig. 2 displays crystal structures of the racemic complexes *rac*-Fe1-C_1_ and *rac*-Fe2-C_2_ representative for the major isomers described in this work. In the obtained stereogenic-at-iron complexes, iron is coordinated by two chelating 1,2,3-triazol-5-ylidenes in addition to two MeCN ligands, while two PF_6_^−^ anions complement the dicationic complexes. The structures reveal an interligand π–π interaction between the *N*-pyridyl moiety of one ligand and the naphthyl ring of the other, while also suggesting a high steric barrier of rotation around the C–C axis between the naphthalene and the triazolyl carbene. This induces the prior configurationally labile ligands to lock their axial chirality as stable atropisomers in the *S*_*a*_ or *R*_*a*_ configuration. In the case of the *C*_2_-symmetrical *rac*-Fe1,2-C_2_ both naphthyl substituents feature the same axial chirality (Δ-*S*_*a*_,*S*_*a*_ and Λ-*R*_*a*_,*R*_*a*_), while in the *C*_1_-symmetrical *rac*-Fe1,2-C_1_ the two naphthyl moieties feature differing axial chirality (Δ-*R*_*a*_,*S*_*a*_ and Λ-*S*_*a*_,*R*_*a*_).

**Fig. 2:**
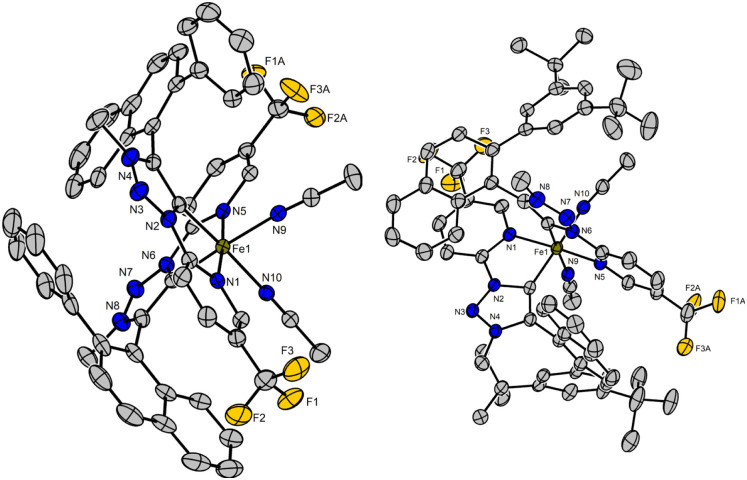
XRD-structures of *rac*-Fe1-C_1_ (left) and *rac*-Fe2-C_2_ (right). The PF_6_^−^ anions, the acetonitrile solvent molecule and the hydrogen atoms are not shown. No disorder is shown. Displacement ellipsoids are shown at 50% probability level at 100 K.

In order to investigate the configurational stability of the chiral iron complexes, we attempted the chiral resolution of the racemic complexes *rac*-Fe1-C_1_ and *rac*-Fe1-C_2_*via* our previously established auxiliary-ligand mediated route by using a chiral fluoro-salicyloxazoline (Salox) (Scheme 2).^14,15^ Accordingly, when we coordinated (*R*)-Salox to *rac*-Fe1-C_1_ under basic conditions, we obtained a 3 : 1 mixture of two diastereomers in overall 99% yield. The two diastereomers were identified as Δ-(*S*_*a*_,*R*_*a*_)-(*R*)-Fe1Aux-a and Δ-(*S*_*a*_,*R*_*a*_)-(*R*)-Fe1Aux-b by NMR (Scheme 2a) (see ESI,† for further details). Interestingly, both diastereomers contain a metal-centred Δ-configuration. Since the racemic starting material was composed from a 1 : 1 mixture of Λ and Δ, it can be concluded that the metal-centred configuration in Fe1-C_1_ is labile. Therefore, the *C*_1_-symmetric complex Fe1-C_1_ was not investigated further.

**Scheme 2:**
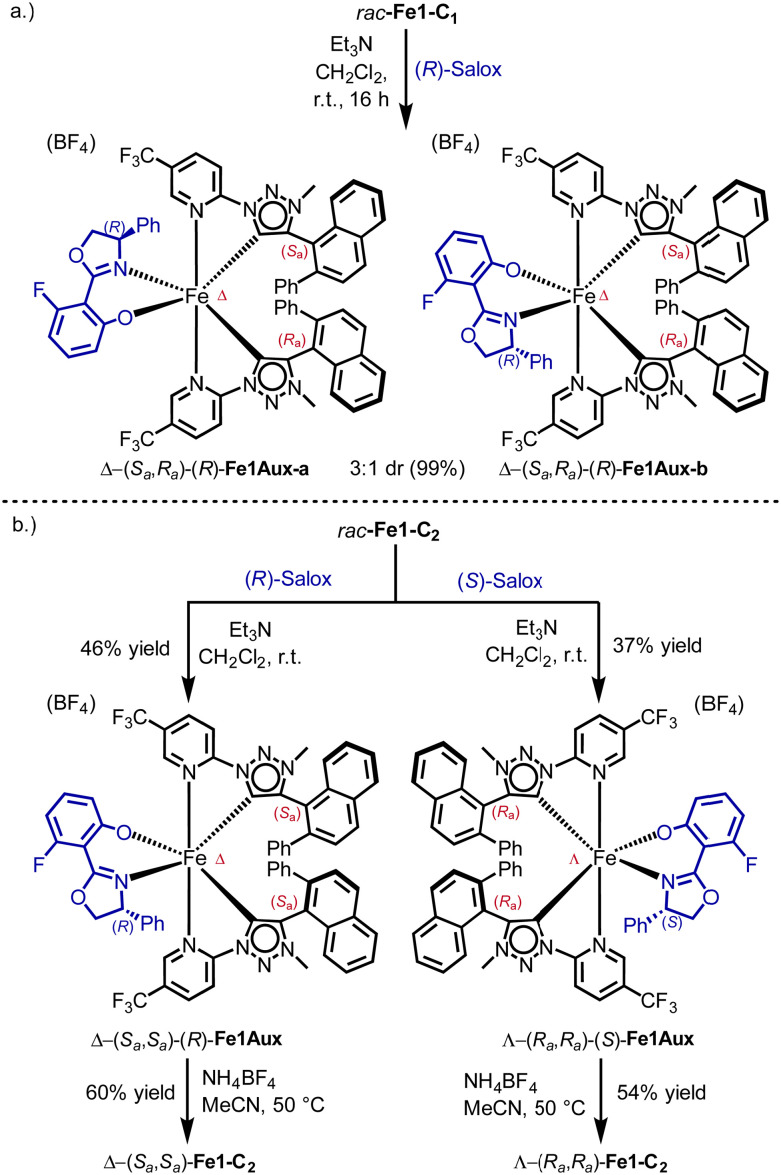
Chiral resolution of *rac*-Fe1-C_2_ and attempted resolution of *rac*-Fe1-C_1_.

In contrast, reacting *rac*-Fe1-C_2_ with (*R*)-Salox furnished Λ-(*R*_*a*_,*R*_*a*_)-(*R*)-Fe1Aux and Δ-(*S*_*a*_,*S*_*a*_)-(*R*)-Fe1Aux with a dr of 1 : 1. However, only the diastereomer Δ-(*S*_*a*_,*S*_*a*_)-(*R*)-Fe1Aux was stable during column chromatography and could be isolated in 46% yield (Scheme 2b). In analogy, Λ-(*R*_*a*_,*R*_*a*_)-(*S*)-Fe1Aux was obtained *via* the same route by employing the mirror-imaged chiral auxiliary (*S*)-Salox. Cleavage of the chiral auxiliary with the weak acid NH_4_BF_4_ at 50 °C then afforded the two enantiomers Δ-(*S*_*a*_,*S*_*a*_)-Fe1-C_2_ (60%) and Λ-(*R*_*a*_,*R*_*a*_)-Fe1-C_2_ (54%). The mirror imaged behaviour of the enantiomers was verified by circular dichroism (Fig. 3). Their enantiomeric purity was confirmed through ^19^F NMR after recoordination of the chiral auxiliary (see ESI,† for details). Thus, it can be concluded that the metal-centred configuration in Fe1-C_2_ is stable in contrast to a facile isomerisation observed in Fe1-C_1_.

**Fig. 3:**
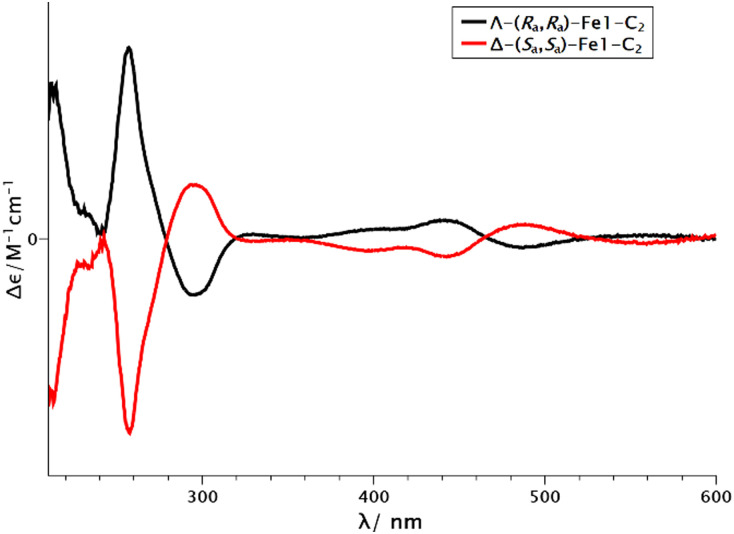
Circular dichroism spectra of Λ-(*R*_a_,*R*_a_)-Fe1-C_2_ (black) and Δ-(*S*_a_,*S*_a_)-Fe1-C_2_ (red) (0.25 mM in MeCN).

Having established the configurational stability of the stereogenic-at-iron complex Fe1-C_2_, we next assessed the atropisomerism^16^ of the ligand 1 employed in this complex. As a result, the rotamers interconvert *via* zero-order kinetics with a half-life of 280 min, corresponding to a rotational energy barrier of 23 kcal mol^−1^ (see ESI,† for details). Thus, ligand 1 can be classified as a LaPlante class 2 atropoisomer.^17^ Axially chiral compounds within this class represent major challenges for further application such as chiral ligands or drug candidates, as they feature rapid interconversion *via* bond-rotation.

However, this rotation is effectively frozen in the corresponding iron complexes,^18,19^ which has direct implications for the configurational stability of the stereogenic iron centre. In the *C*_1_-symmetric complex Fe1-C_1_, both the *S*_a_- and *R*_a_-configured ligand 1 is present, meaning that inversion of the metal-centred configuration (Λ *vs.* Δ) leads to the formation of enantiomers (Fig. 4). As demonstrated, this isomerisation occurs rapidly, consistent with our prior findings on the configurational lability of iron MIC complexes. In contrast, the *C*_2_-symmetric complex Fe1-C_2_ contains axially chiral ligands with identical configurations (either *S*_a_ or *R*_a_). Here, inversion of the metal-centred configuration (Λ *vs.* Δ) results in the formation of diastereomers. For instance, Δ-(*S*_a_,*S*_a_)-Fe1-C_2_ would isomerize into its diastereomer Λ-(*S*_a_,*S*_a_)-Fe1-C_2_*. Notably, this diastereomer is not observed experimentally. Structural modelling suggests that steric clashes between the phenyl substituents on the naphthyl moieties prevent this isomerisation. Thus, the configuration of the metal centre in Δ-(*S*_a_,*S*_a_)-Fe1-C_2_ and its enantiomer Λ-(*R*_a_,*R*_a_)-Fe1-C_2_ is controlled thermodynamically rather than kinetically by the axial chirality of the ligands.

**Fig. 4:**
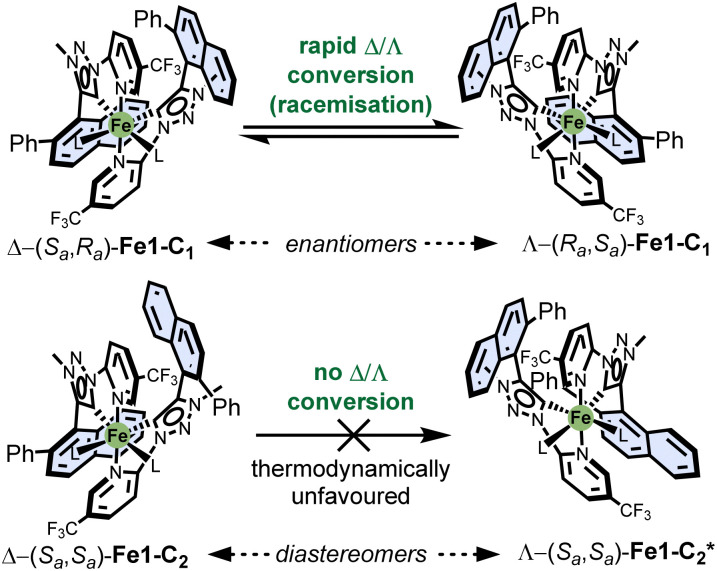
Configurational stabilities of the discussed stereogenic-at-iron complexes.

Finally, we investigated the catalytic properties of the stereogenic-at-iron complexes. We found that Δ-(*S*_a_,*S*_a_)-Fe1-C_2_ exhibited high catalytic activity in the ring contraction of isoxazole 6 to the chiral 2*H*-azirine 7 (Table 2).^20^ Initial experiments employing 0.1 mol% of Δ-(*S*_a_,*S*_a_)-Fe1-C_2_ at room temperature achieved full conversion after only 15 minutes, albeit with a moderate enantiomeric excess (ee) of 72% (entry 1). However, decreasing the temperature to −40 °C improved the ee to 96%, while an increased catalyst loading of 1.0 mol% was necessary to achieve full conversion after 24 hours (entry 2). Further reducing the temperature to −50 °C and extending the reaction time to 40 hours, while using 1.5 mol% of the catalyst, provided the best result with an isolated yield of 93% and 97% ee (entry 3). Although catalytic asymmetric versions of this ring contraction have been reported, the enantioselectivity demonstrated here is unprecedented for iron-catalysed systems and rivals that of ruthenium catalysts.^20^

**Table 2:** Catalytic ring contraction of an isoxazole to a chiral 2*H*-azirine^a^

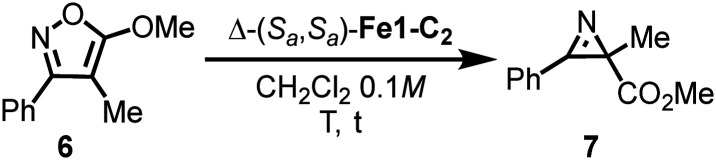					
Entry	Catalyst loading	*T* (°C)	*t* (h)	Yield^b^	ee (%)
1	0.1 mol%	r.t.	0.25	99	72
2	1.0 mol%	−40	24	98 (95)^c^	96
3	1.5 mol%	−50	40	95 (93)^c^	97

a Reaction conditions: Under N_2_ atmosphere. A solution of Δ-(*S*_a_,*S*_a_)-Fe1-C_2_ (0.1–1.5 mol%) in CH_2_Cl_2_ (0.5 mL) was added to substrate 6 (10 mg, 0.05 mmol) and stirred for the indicated time and temperature.

b Yield was determined *via*^1^H NMR analysis with 1,3,5-trimethoxybenzene as standard.

c Isolated yield.

In summary, we here presented an example in which the metal-centred configuration of a stereogenic-at-iron catalyst is regulated by axially chiral ligands. Notably, the free ligand undergoes rapid atropisomerisation but is frozen into a single configuration only upon coordination to the metal. Future work will investigate the use of related non-racemic ligands with fixed axial chirality.

This project has received funding from the European Research Council (ERC) under the European Union's Horizon 2020 research and innovation programme (grant agreement No 883212).

## Data availability

The ESI,† contains detailed synthetic procedures and complete characterisation data for all new compounds, as well as CD spectra of all non-racemic iron complexes.

## Conflicts of interest

There are no conflicts to declare.

## Supplementary Material

CC-061-D4CC06227B-s001

CC-061-D4CC06227B-s002
