# Supplementary material for: Coordination-induced axial chirality controls the metal-centred configuration in a stereogenic-at-iron catalyst
Source: Chem Commun (Camb). 2025 Jan 8;61(9):1894–7. doi: 10.1039/d4cc06227b (PMC11708482; doi:10.1039/d4cc06227b)
Supplement: CC-061-D4CC06227B-s001 [file CC-061-D4CC06227B-s001.pdf]

Supplementary Information (SI) for Chemical Communications.
This journal is © The Royal Society of Chemistry 2025

Supplementary Information

Coordination-Induced Axial Chirality Controls the Metal-Centred Configuration in a Stereogenic-at-Iron-Catalyst

Benedikt Buchberger, Nemrud Demirel, Xiulan Xie, Sergei I. Ivlev and Eric Meggers*

Department of Chemistry, Philipps-Universität Marburg, Hans-Meerwein-Strasse 4, 35041 Marburg, Germany

*Corresponding author: meggers@chemie.uni-marburg.de

Table of Contents

1 General Information **S2**

2 Ligand Synthesis **S3**

3 Iron Complex Synthesis **S12**

3.1 Initial Optimisation of Isomer Control S12

3.2 Synthesis of *rac*-Fe1 and *rac*-Fe2 .... S16

3.3 Remarks for Table 1 .... S18

4 Auxiliary Complex Synthesis **S19**

4.1 Configurational stability of Fe1-C_1 .... S20

4.1.1 Diastereomeric Mixture in Auxiliary-Complex Formation S20

4.1.2 NMR Studies on Diastereomeric Mixture S21

4.2 Configurational Stability of Fe1-C_2 .... S28

5 Auxiliary Cleavage **S30**

6 Configurational Stability of Ligand 1 .... **S32**

7 Enantioselective Ring Contraction of Isoxazoles to 2*H*-azirines **S33**

8 Characterisation of Fe(II)-Complexes **S34**

9 NMR-Spectra **S40**

10 Chiral HPLC Traces **S58**

11 CD-Spectra **S60**

12 Structure elucidation of C_2-Isomer and C_1-Isomer for Fe1 and Fe2 .... **S61**

12.1 Relation of Possible Isomers S61

12.2 Single Crystal X-Ray Diffraction S62

13 References **S67**

1 General Information

All reactions were carried out under a nitrogen atmosphere in oven-dried glassware unless noted otherwise. Solvents were distilled under nitrogen from sodium/benzophenone (THF, Et_2O) or calcium hydride (MeCN, CH_2Cl_2, $CHCl_3$, toluene) prior to use. Reagents that were purchased from commercial suppliers were used without further purification. Flash column chromatography was performed with silica gel 60 M from Macherey-Nagel (irregular shaped, 230−400 mesh, pH 6.8, pore volume: 0.81 mL g^{-1}, mean pore size: 66 Å, specific surface: 492 m^2 g^{-1}, particle size distribution: 0.5% < 25 µm and 1.7% > 71 µm, water content: 1.6%). ^{1}H NMR, ^{13}C {^{1}H} NMR and ^{19}F{^{1}H} NMR spectra were recorded on a Bruker AV NEO 300 MHz, AV II 300 MHz, AV III 500 MHz, AV III HD 500 MHz, or AV NEO 600 MHz spectrometer at ambient temperature. Chemical shift values δ are reported in ppm with the solvent resonance as internal standard. ^{19}F{^{1}H} NMR spectra were calibrated to trichlorofluoromethane ($CFCl_3$, δ = 0 ppm) as external standard. IR spectra were recorded on a Bruker Alpha FT−IR spectrometer. Melting points (MPs) were determined on a Mettler Toledo MP70 using one end closed capillary tubes. Chiral HPLC was performed on an Agilent 1200 or 1260. CD spectra were acquired with a JASCO J-810 CD spectropolarimeter (parameters: 600−200 nm, 1 nm band width, 50 nm min^{-1} scanning speed, accumulation of 3 scans). High-resolution mass spectrometry was performed on a Finnigan LTQ-FT Ultra mass spectrometer (Thermo Fischer Scientific) using ESI, EI or APCI as ionization source. Tetrazole **4**[1] and isoxazole **6**[2] were synthesized after a literature procedure.

2 Ligand Synthesis

Synthesis of alkyne 3

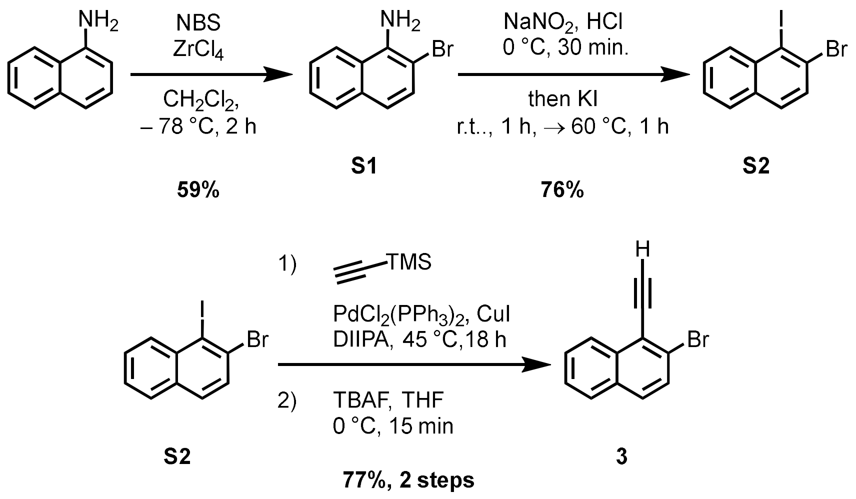

2-bromonaphthalen-1-amine (S1)

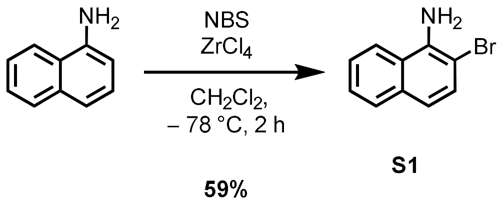

Following a modified procedure from ZHANG *et al.*[3] in an oven dried SCHLENK flask, a solution of NBS (9.94 g, 55.9 mmol, 1.00 equiv.) in CH_2Cl_2 (150 mL, 0.37 *M*) was cooled to −78 °C. Then ZrCl4 (651 mg, 2.80 mmol, 0.05 equiv.) and 1-naphtylamine (8.00 g, 55.9 mmol, 1.00 equiv.) were added. The solution was stirred for 2 h at that temperature before it was quenched by adding a sat. *aq*. $NaHCO_3$-solution. The phases were separated and the aqueous layer was extracted with CH_2Cl_2 (3 x 20 mL) and the combined organic layers were washed with sat. *aq*. $NaHCO_3$ solution (1 x 200 mL), brine (1 x 100 mL), dried over Na_2SO_4, filtered and the solvent removed under reduced pressure. The crude was purified by flash column chromatography (silica gel, *n*-pentane/EtOAc 25:1 → 20:1) to yield the aminonapthalene **S1** as a pink solid (7.27 g, 222 mmol, 59%).

Analytical data of **S1** are in agreement with published data.[4]

TLC (*n*-pentane/EtOAc)**:** R_f = 0.56. **^{1}H NMR** (300 MHz, $CDCl_3$): δ (ppm) = 7.86–7.71 (m, 2H), 7.54–7.42 (m, 2H), 7.17 (d, *J* = 8.7 Hz, 1H), 4.64 (s, 2H). **^{13}C NMR** (75 MHz, $CDCl_3$): δ (ppm) = 139.60, 133.39, 129.89, 128.77, 126.21, 125.86, 123.86, 121.00, 119.48, 104.39. **HRMS**. ESI (+); *m/z* calcd. for $C_{10}H_8BrNH$: $[M + H]^+$: 223.9892, found: 223.9892.

2-bromo-1-iodonaphthalene (S2)

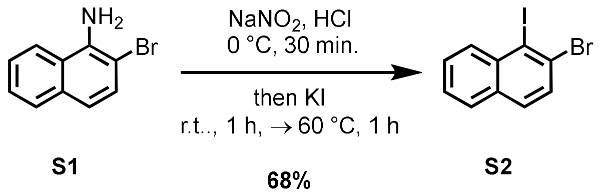

Following a modified procedure from JANČAŘÍK *et al.*[5] a round-bottom flask was charged with aminonaphtalene **S1** (6.00 g, 27 mmol, 1.00 equiv.) followed by slow addition of conc. hydrochloric acid (30 mL). The suspension was stirred at room temperature for 30 min before it was cooled to 0 °C by an ice bath and addition of ice (100 g) to the reaction mixture. Then $NaNO_2$ (2.14 g, 31.1 mmol, 1.15 equiv.) was added in small portions; the pink suspension immediately became a green-yellow solution. After full addition the mixture was stirred for 30 min at 0 °C. Then KI (17.9 g, 108 mmol, 4.00 equiv.) was added in one portion and the mixture was allowed to warm up to room temperature at which the now red solution was further stirred for 1 h, followed by stirring for at 60 °C for further 1 h. The resulting dark mixture was quenched by a sat. *aq.* $Na_2S_2O_3$ solution, extracted with CH_2Cl_2 (3 x 100 mL) washed with sat. *aq.* $Na_2S_2O_3$ solution (1 x 150 mL), brine (1 x 150 mL), dried over Na_2SO_4, filtered and the solvent removed under reduced pressure. The crude was purified by flash-column chromatography (silica gel, *n*-pentane) to yield the iodonapthalene **S2** as yellow solid (6.13 g, 18.4 mmol, 68%).

Analytical data of **S2** are in agreement with published data.[5]

TLC (*n*-pentane)**:** R_f = 0.60. **^{1}H NMR** (300 MHz, $CDCl_3$): δ (ppm) = 8.24 (dd, *J* = 8.7, 1.2 Hz, 1H), 7.82–7.73 (m, 1H), 7.69 (d, *J* = 1.4 Hz, 2H), 7.61–7.48 (m, 2H). **^{13}C NMR** (75 MHz, $CDCl_3$): δ (ppm) = 136.75, 133.97, 131.96, 130.04, 130.02, 129.68, 128.85, 128.51, 126.83, 106.75. **HRMS** EI (+); *m/z* calcd. for $C_{10}H_6BrI$: $[M]^+$: 331.86976, found: 331.87035.

2-bromo-1-ethynylnaphthalene (3)

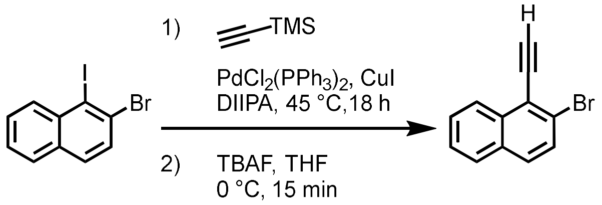

S2 **3**

77%, 2 steps

Following a modified procedure from AKHMETOV *et al.* [6] an oven dried SCHLENK flask was charged with iodonaphtalene **S2** (6.50 g, 19.5 mmol, 1.00 equiv.), $PdCl_2(PPh_3)_2$ (684 mg, 0.976 mmol, 0.05 equiv.), CuI (372 mg, 1.95 mmol, 0.10 equiv.), evacuated and flushed with nitrogen, suspended in diisopropylamin (36 mL, 0.5 *M*) and degassed by bubbling with nitrogen for 30 min. Then, TMS-acetylene (3.25 mL, 23.4 mmol, 1.20 equiv.) was added and the mixture stirred at 45 °C for 18 h. As full conversion was observed via TLC, the mixture was diluted with *n*-pentane (50 mL) and filtered over a pad of Celite followed by washing with a sat. *aq*. NH_4Cl solution (3 x 50 mL) and brine (1 x 25 mL), dried over Na_2SO_4, filtered and the solvent removed under reduced pressure. The resulting crude brown oil was used directly in the next step.

The crude oil from the previous step was dissolved in THF (200 mL, 0.1 *M*), cooled to 0 °C and TBAF (70% in H_2O, diluted with THF) (8.02 g, 21.5 mmol, 1.10 equiv.) was added dropwise and stirring continued for 15 min at that temperature. Upon completion, the mixture was filtered over a plug of Celite and Silica to remove salts and water and eluted with CH_2Cl_2 (100 mL) before the solvents were removed under reduced pressure. The crude was purified by flash-column chromatography (silica gel, *n*-pentane) to yield the alkyne **3** as brown solid (3.47 g, 15.0 mmol, 77% over two steps).

Analytical data of **3** are in agreement with published data. [7]

TLC (*n*-pentane)**:** R_f = 0.52. **^{1}H NMR** (300 MHz, $CDCl_3$): δ (ppm) = 8.36 (d, *J* = 8.2 Hz, 1H), 7.82 (d, *J* = 8.0 Hz, 1H), 7.70 (d, *J* = 8.9 Hz, 1H), 7.68–7.48 (m, 3H), 3.82 (s, 1H). **^{13}C NMR** (75 MHz, $CDCl_3$): δ (ppm) = 134.92, 131.75, 130.09, 129.63, 128.41, 128.06, 126.86, 126.36, 125.29, 121.45, 87.22, 80.44. **HRMS** EI (+); *m/z* calcd. for $C_{12}H_7Br$: $[M]^+$: 229.97311, found: 229.97307.

Overview for the synthesis of ligands 1 and 2 from alkyne 3

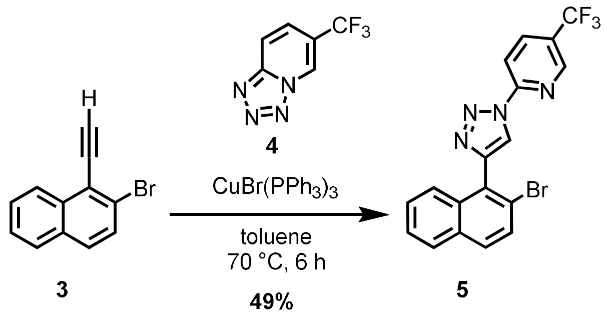

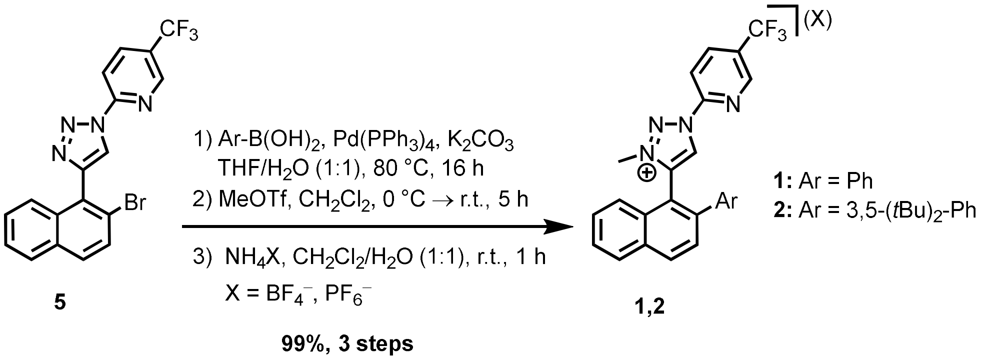

2-(4-(2-bromonaphthalen-1-yl)-1H-1,2,3-triazol-1-yl)-5-(trifluoromethyl)pyridine (5)

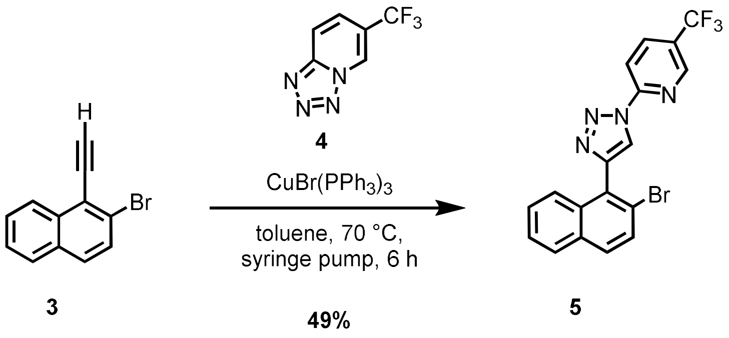

Following a modified procedure from BOLJE *et al.* [8] An oven dried SCHLENK flask was charged with tetrazole **4** (493 mg, 2.62 mmol, 1.20 equiv.) and $CuBr(PPh_3)_3$ (111 mg, 0.12 mmol, 0.05 equiv.), evacuated and flushed with nitrogen, then dissolved in toluene (10 mL) and degassed for 30 min by bubbling with nitrogen. In a separate flask, alkyne **3** (550 mg, 2.38 mmol, 1.00 equiv.) was dissolved in toluene (20 mL) and degassed for 30 min by bubbling with nitrogen. Then the solution containing the alkyne was added to the flask containing the dissolved solids with a syringe pump over the course of 5 h while stirring the mixture at 70 °C. After full addition, stirring at that temperature was continued until full conversion was confirmed by TLC (1 h). Then the solvents were removed under reduced pressure, the crude was absorbed onto silica and purified by flash-column chromatography (silica gel, 25:1 → 20:1 → 15:1) to afford the bromonapthalene triazole **5** as orange solid (493 mg, 1.18 mmol, 49%).

TLC (*n*-pentane/EtOAc 10:1)**:** R_f = 0.33. **MP**: 140 °C. **^{1}H NMR** (500 MHz, $CDCl_3$): δ (ppm) = 8.86 (s, 1H), 8.83 (dd, *J* = 1.6, 0.9 Hz, 1H), 8.50 (d, *J* = 8.6 Hz, 1H), 8.23 (dd, *J* = 8.6, 1.7 Hz, 1H), 7.88 (d, *J* = 8.1 Hz, 1H), 7.82 (d, *J* = 8.5 Hz, 1H), 7.77 (d, *J* = 8.8 Hz, 1H), 7.72 (d, *J* = 9.1 Hz, 1H), 7.54 (ddd, *J* = 8.1, 6.8, 1.3 Hz, 1H), 7.48 (ddd, *J* = 8.3, 6.8, 1.4 Hz, 1H).**^{19}F NMR** (282 MHz, $CDCl_3$): δ (ppm) = −62.19 (s, 3F, CF_3). **^{13}C NMR** (126 MHz, $CDCl_3$): δ (ppm) = 151.47, 146.35 (q, $^3J_{C,F}$ = 4.2 Hz), 145.60, 136.89 (q, $^3J_{C,F}$ = 3.3 Hz), 134.16, 132.51, 130.92, 130.07, 128.33, 128.00, 127.72, 126.71 (q, $^2J_{C,F}$ = 33.7 Hz), 126.62, 126.02, 123.39, 123.19 (d, $^1J_{C,F}$ = 272.3 Hz), 122.35, 113.87. **IR** (neat): $\tilde{\nu}$ (cm $^{-1}$) = 3173 (w), 3120 (w), 3061 (w), 1609 (m), 1594 (w), 1581 (w), 1546 (w), 1494 (m), 1448 (m), 1394 (w), 1369 (w), 1325 (s), 1262 (w), 1225 (m), 1192 (w), 1171 (m), 1117 (s), 1081 (m), 1034 (s), 1014 (w), 995 (w), 954 (w), 942 (w), 852 (m), 828 (m), 799 (s), 774 (m), 760 (w), 735 (m), 723 (w), 716 (w), 668 (w), 658 (w), 649 (w), 635 (w), 611 (w), 597 (w), 561 (w), 535 (w), 518 (w), 484 (m), 432 (w), 422 (m). **HRMS** ESI (+); *m/z* calcd. for $C_{18}H_{10}BrF_3N_4H$: $[M + H]^+$: 419.0114, found: 419.0125.

General procedure A: Synthesis of 2-arylnaphtalenes S3a and S3b

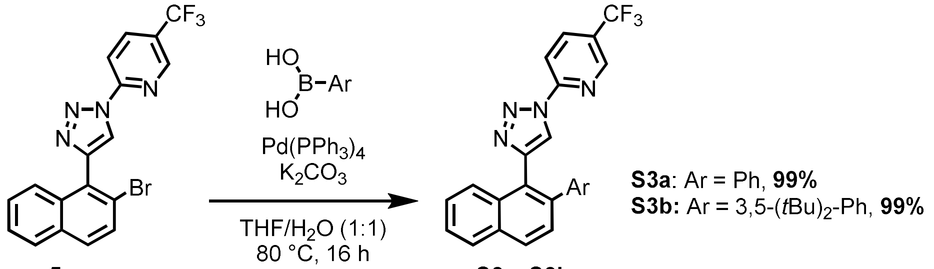

5

S3a, S3b

Following a modified procedure from NIE *et al.* [9] An oven dried SCHLENK flask was charged with bromonapthalene triazole **5** (1.00 equiv.), arylboronic acid (1.20 equiv.), $Pd(PPh_3)_4$ (0.05 equiv.) and K_2CO_3 (3.00 equiv.), evacuated and flushed with nitrogen and then dissolved in a mixture of previously degassed 1:1 (v/v) H_2O/THF (0.1 *M*), heated to 80 °C and stirred at this temperature for 16 h. After cooling to room temperature, the layers were separated, and the aqueous phase was extracted with EtOAc (3 x). The combined organic layers were washed with brine (1 x), dried over Na_2SO_4, filtered and the solvent removed under reduced pressure. The crude was absorbed onto silica and purified by flash-column chromatography (silica gel, *n*-pentane/EtOAc 15:1) to yield the arylnapthalene triazole **S3a** or **S3b** as orange solid.

General procedure B: Synthesis of 3-Methyltriazolium triflate 1 and 2

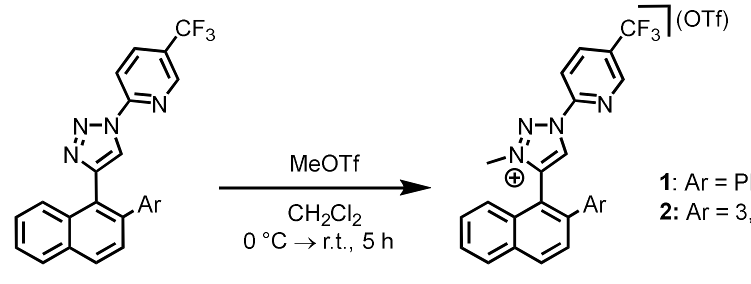

S3 **1,2**

1: Ar = Ph, **99%**
2: Ar = 3,5-(*t*Bu)$_2$-Ph, **99%**

Following a modified procedure from BOLJE *et al.* [10] An oven dried SCHLENK flask was charged with arylnapthalene triazole **S3a** or **S3b** (1.00 equiv.), evacuated and flushed with nitrogen and dissolved in CH_2Cl_2 (0.2 *M*) before the solution was cooled to 0 °C and MeOTf (1.20 equiv.) was added. After stirring for 10 minutes at that temperature, the solution was allowed to warm up to room temperature and stirred for further 5 h. After full conversion was confirmed via TLC, the solution was then diluted with CH_2Cl_2 and filtered over a plug of silica gel and impurities were washed off with CH_2Cl_2. Then, the product was eluted with a mixture of CH_2Cl_2/MeOH 5:1. The solvents were removed under reduced pressure to yield the analytical pure 3-methyltriazolium triflate **1** or **2** as an off-white solid.

General procedure C: Optional anion exchange for ligand 1 and 2

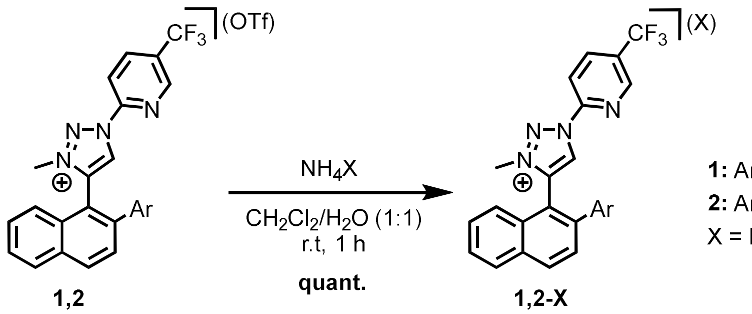

1,2 **1,2-X**

1: Ar = Ph
2: Ar = 3,5-(*t*Bu)$_2$-Ph
X = BF_4^-, PF_6^-

A round bottom flask was charged with 3-methyltriazolium triflate **1** or **2** and a mixture of CH_2Cl_2/H_2O (1:1 v:v, 0.2 *M*) was added before NH_4X (X = BF_4^- or PF_6^-, 10 equiv.) was added at room temperature. Then the mixture was vigorously stirred for 1 h, before the phases were separated and the aqueous phase extracted with CH_2Cl_2 (1 x). Then the combined organic phases were washed with water, dried over Na_2SO_4, filtered and the solvents removed under reduced pressure to yield the analytical pure product 3-methyltriazolium **1-BF$_4$**, **2-BF$_4$**, **1-PF$_6$** or **2-PF$_6$** as an off-white solid in quantitative yield. Full exchange of the respective anion was probed by ^{19}F NMR.

2-(4-(2-phenylnaphthalen-1-yl)-1H-1,2,3-triazol-1-yl)-5-(trifluoromethyl)pyridine (S3a)

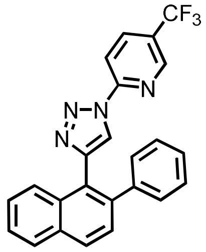

S3a

Following general procedure A, arylnaphtalene triazole **S3a** (593 mg, 1.42 mmol, 99%) was obtained as a yellow solid from the corresponding bromonaphtalene triazole **5** (600 mg, 1.43 mmol).

TLC (*n*-pentane/EtOAc 10:1)**:** R_f = 0.50. **MP**: 152 °C. **^{1}H NMR** (500 MHz, $CDCl_3$): δ (ppm) = 8.68 (dd, *J* = 1.6, 0.8 Hz, 1H), 8.37 (d, *J* = 8.6 Hz, 1H), 8.18 (s, 1H), 8.14 (dd, *J* = 8.7, 2.1 Hz, 1H), 8.00 (d, *J* = 8.9 Hz, 1H), 7.93 (dd, *J* = 8.4, 1.3 Hz, 1H), 7.84 (d, *J* = 8.3 Hz, 1H), 7.59 (d, *J* = 8.5 Hz, 1H), 7.55–7.46 (m, 2H), 7.28–7.17 (m, 5H). **^{19}F NMR** (282 MHz, $CDCl_3$): δ (ppm) = −62.23 (s, 3F, CF_3). **^{13}C NMR** (126 MHz, $CDCl_3$): δ (ppm) = 151.35, 146.18 (q, $^3J_{C,F}$ = 4.1 Hz), 145.99, 141.50, 140.91, 136.68 (q, $^3J_{C,F}$ = 3.3 Hz), 133.13, 132.91, 129.95 (s, 2C), 129.57, 128.23, 128.16 (s, 2C), 128.08, 127.18, 127.07, 126.41 (q, $^2J_{C,F}$ = 33.7 Hz), 126.27, 126.23, 125.16, 123.18 (q, $^1J_{C,F}$ = 272.5 Hz), 121.85, 113.66. **IR** (neat): $\tilde{\nu}$ (cm^{-1}) = 3161 (w), 3113 (w), 3054 (w), 1604 (m), 1491 (m), 1444 (m), 1389 (w), 1367 (w), 1324 (s), 1265 (w), 1218 (m), 1191 (w), 1168 (m), 1141 (w), 1126 (s), 1082 (m), 1030 (s), 992 (m), 969 (w), 946 (w), 915 (w), 864 (m), 830 (s), 793 (w), 763 (s), 749 (w), 730 (w), 714 (w), 702 (s), 685 (w), 656 (w), 635 (w), 615 (w), 579 (w), 545 (w), 537 (w), 527 (w), 475 (m), 432 (m). **HRMS** ESI (+); *m/z* calcd. for $C_{24}H_{15}F_3N_4Na$ $[M + Na]^+$: 439.1141, found: 439.1132.

(4-(2-(3,5-di-tert-butylphenyl)naphthalen-1-yl)-1H-1,2,3-triazol-1-yl)-5-(trifluoromethyl)pyridine (S3b)

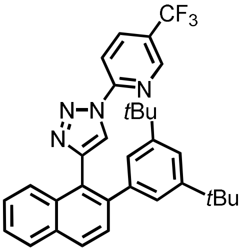

S3b

Following general procedure A, arylnaphtalene triazole **S3b** (502 mg, 0.95 mmol, 99%) was obtained as a yellow solid from the corresponding bromonaphtalene triazole **5** (400 mg, 0.95 mmol).

TLC (*n*-pentane/EtOAc 10:1)**:** R_f = 0.55. **MP**: 144 °C. **^{1}H NMR** (500 MHz, $CDCl_3$): δ (ppm) = 8.69 (dd, *J* = 1.6, 0.8 Hz, 1H), 8.36 (d, *J* = 8.6 Hz, 1H), 8.15 (dd, *J* = 8.6, 1.7 Hz, 1H), 8.10 (s, 1H), 8.02 (d, *J* = 8.2 Hz, 1H), 7.95 (dd, *J* = 8.3, 1.3 Hz, 1H), 7.89 (dd, *J* = 8.2, 0.8 Hz, 1H), 7.69 (d, *J* = 8.5 Hz, 1H), 7.55–7.47 (m, 2H), 7.24 (t, *J* = 1.8 Hz, 1H), 7.11 (d, *J* = 1.8 Hz, 2H), 1.20 (s, 18H). **^{19}F NMR** (282 MHz, $CDCl_3$): δ (ppm) = −62.21 (s, 3F, CF_3). **^{13}C NMR** (126 MHz, $CDCl_3$): δ (ppm) = 151.39, 150.42 (2C), 146.41, 146.21 (q, $^3J_{C,F}$ = 4.2 Hz), 141.80, 140.34, 136.71 (q, $^3J_{C,F}$ = 3.3 Hz), 133.22, 132.85, 129.53, 128.19, 128.07, 127.06, 126.30, 126.20 (q, $^2J_{C,F}$ = 34.0 Hz), 126.11, 125.19, 124.67 (2C), 123.20 (q, $^1J_{C,F}$ = 272.4 Hz), 121.84,

120.67, 113.50, 34.91 (2C), 31.48 (6C). **IR** (neat): $\tilde{\nu}$ (cm $^{-1}$) = 3058 (w), 2955 (w), 2904 (w), 2868 (w), 1738 (w), 1604 (m), 1493 (m), 1443 (w), 1393 (w), 1363 (w), 1323 (s), 1264 (w), 1247 (w), 1222 (m), 1165 (w), 1130 (s), 1080 (m), 1023 (s), 991 (m), 939 (w), 919 (w), 899 (w), 878 (w), 847 (w), 822 (s), 791 (w), 748 (m), 715 (m), 688 (w), 663 (w), 635 (w), 611 (w), 597 (w), 568 (w), 554 (w), 532 (w), 516 (w), 480 (w), 428 (w). **HRMS** ESI (+); *m/z* calcd. for: $C_{32}H_{31}F_3N_4H$ [M + H]$^+$: 529.2574, found: 529.2585.

3-methyl-4-(2-phenylnaphthalen-1-yl)-1-(5-(trifluoromethyl)pyridin-2-yl)-1H-1,2,3-triazol-3-ium tetrafluoroborate (1)

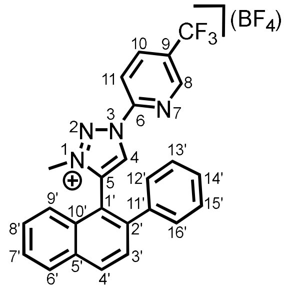

1

Following general procedure B, ligand **1** (415 mg, 0.72 mmol, 99%) was obtained as an off white solid from the corresponding arylnaphtalene triazole **S3a** (300 mg, 0.72 mmol).

TLC (CH_2Cl_2/MeOH 10:1)**:** R_f = 0.32. **MP**: 132 °C. **^{1}H NMR** (600 MHz, CD_3CN): δ (ppm) = 9.40 (s, 1H, *H*4), 9.03 (d, *J* = 2.3 Hz, 1H, *H*8), 8.54 (dd, *J* = 8.6 Hz, 2.3 Hz, 1H, *H*10), 8.36 (d, *J* = 8.5, 1H, *H*4'), 8.31 (d, *J* = 8.6 Hz, 1H, *H*11), 8.17 (d, *J* = 7.8 Hz, 1H, *H*6'), 7.78 (dd, *J* = 8.6, 1H, *H*3'), 7.74–7.68 (m, 3H, *H*7', *H*8', *H*9'), 7.43–7.37 (m, 3H, *H*13', *H*14', *H*15'), 7.34–7.32 (m, 2H, *H*12', *H*16'), 3.87 (s, 3H, NC*H*$_3$). **^{19}F NMR** (282 MHz, CD_3CN): δ (ppm) = −62.45 (s, 3F, CF_3), –151.78 ($^{10}BF_4^-$), –151.84 ($^{11}BF_4^-$). **^{13}C NMR** (125 MHz, CD_3CN): δ (ppm) = 149.89, 147.88 (q, $^3J_{C,F}$ = 3.9 Hz), 143.98, 143.06, 139.86 (q, $^3J_{C,F}$ = 3.4 Hz), 139.82, 134.01, 133.62, 132.72, 130.17 (2C), 130.11, (q, $^4J_{C,F}$ = 34.4 Hz), 130.08 (2C), 129.85, 129.73, 129.53, 129.33, 129.07, 128.41, 125.38, 123.88 (q, $^1J_{C,F}$ = 272.5 Hz, Ar-*C*F_3), 116.86, 116.36, 39.95. **IR** (neat): $\tilde{\nu}$ (cm $^{-1}$) = 3168 (w), 3067 (w), 1603 (m), 1551 (w), 1485 (w), 1442 (w), 1383 (w), 1323 (s), 1270 (w), 1255 (s), 1226 (w), 1191 (w), 1145 (s), 1073 (m), 1028 (s), 1018 (w), 998 (w), 981 (w), 961 (w), 881 (w), 867 (w), 837 (m), 795 (w), 777 (s), 757 (w), 740 (w), 711 (m), 700 (w), 674 (w), 651 (w), 635 (s), 571 (m), 548 (w), 516 (m), 474 (w), 437 (w), 429 (m). **HRMS** ESI (+); *m/z* calcd. for $C_{25}H_{18}F_3N_4$ [M]$^+$: 431.1478, found: 431.1466.

3-methyl-4-(2-(3,5-di-tert-butylphenyl)naphthalen-1-yl)-1-(5-(trifluoromethyl)pyridin-2-yl)-1H-1,2,3-triazol-3-ium trifluoromethanesulfonate (2)

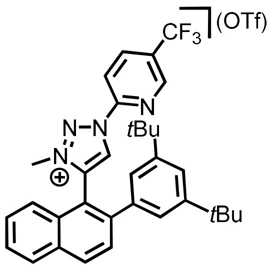

2

Following general procedure B, ligand **2** (247 mg, 0.36 mmol, 99%) was obtained as an off white solid from the corresponding arylnaphtalene triazole **S3b** (191 mg, 0.0.63 mmol).

TLC (CH_2Cl_2/MeOH 10:1)**:** R_f = 0.34. **MP**: 127 °C. **^{1}H NMR** (500 MHz, $CDCl_3$): δ (ppm) = 9.10 (s, 1H), 8.85 (d, *J* = 2.2 Hz, 1H), 8.56 (d, *J* = 8.5 Hz, 1H), 8.37 (dd, *J* = 8.6, 2.3 Hz, 1H), 8.23 (d, *J* = 8.5 Hz, 1H), 8.09 (d, *J* = 8.5 Hz, 1H), 8.01 (d, *J* = 8.1 Hz, 1H), 7.73 (t, *J* = 7.0 Hz, 1H), 7.72 (d, *J* = 8.5 Hz, 1H), 7.65 (t, *J* = 7.4 Hz, 1H), 7.26 (s, 1H), 7.03 (d, *J* = 1.8 Hz, 2H), 3.87 (s, 3H), 1.22 (s, 18H). **^{19}F NMR** (282 MHz, $CDCl_3$): δ (ppm) = −62.44 (s, 3F, CF_3), −78.42 (s, 3F, OTf). **^{13}C NMR** (125 MHz, $CDCl_3$): δ (ppm) = 152.00 (2C), 148.82, 146.37 (q, $^3J_{C,F}$ = 4.0 Hz), 143.37, 142.80, 138.41 (q, $^3J_{C,F}$ = 3.3 Hz), 138.31, 133.32, 132.55, 132.13, 129.99 (q, $^2J_{C,F}$ = 34.2 Hz), 129.97, 128.34, 128.00, 127.87, 127.33, 125.56, 123.58 (2C), 122.58 (q, $^1J_{C,F}$ = 273.1 Hz, Ar-*C*F_3), 122.16, 120.70 (q, $^1J_{C,F}$ = 320.4 Hz, $OS(O)_2$-*C*F_3), 117.04, 115.98, 39.04, 35.04 (2C), 31.40 (6C). **IR** (neat): $\tilde{\nu}$ (cm^{-1}) = 3061 (w), 2961 (w), 2869 (w), 1602 (w), 1553 (w), 1507 (w), 1482 (w), 1435 (w), 1396 (w), 1364 (w), 1326 (s), 1255 (s), 1224 (w), 1134 (s), 1075 (m), 1030 (s), 997 (w), 939 (w), 899 (w), 880 (w), 827 (m), 793 (w), 755 (m), 719 (w), 70 (w), 656 (w), 637 (s), 572 (w), 544 (w), 52 (m), 480 (w), 430 (w). **HRMS** ESI (+); *m/z* calcd. for $C_{33}H_{34}F_3N_4$ $[M]^+$: 543.2730, found: 543.2715.

3 Iron Complex Synthesis

3.1 Initial Optimisation of Isomer Control

Synthesis of Ag-carbene S4

In an oven dried Schlenk tube 3 Å molecular sieves (7.3 mg, 1.0 g/mmol of **2**) was freshly activated by heating at 250 °C for 30 min. under vacuum. After cooling to room temperature, the tube was charged with ligand **2** (5 mg, 7.3 µmol, 1.00 equiv.), KCl (5.4 mg, 73 µmol, 10.0 equiv.) and Ag_2O (5.9 mg, 25 µmol, 3.50 equiv.), evacuated and flushed with nitrogen. Subsequently the solids were dispersed in dry MeCN (0.65 mL, 0.011 *M*) and stirred for the indicated time and temperature under exclusion of light. After completion, the mixture was filtered over a pad of Celite and rinsed with MeCN. The solvents were removed under reduced pressure on a rotary evaporator at 30 °C. Then, the residues were dispersed in CH_2Cl_2 and filtered over a pad of Celite to remove insoluble salts. Removal of the solvents as described above gave the Ag-carbene-dimer **S4**.

Table S1: Optimisation for the synthesis of Ag-carbene **S4**

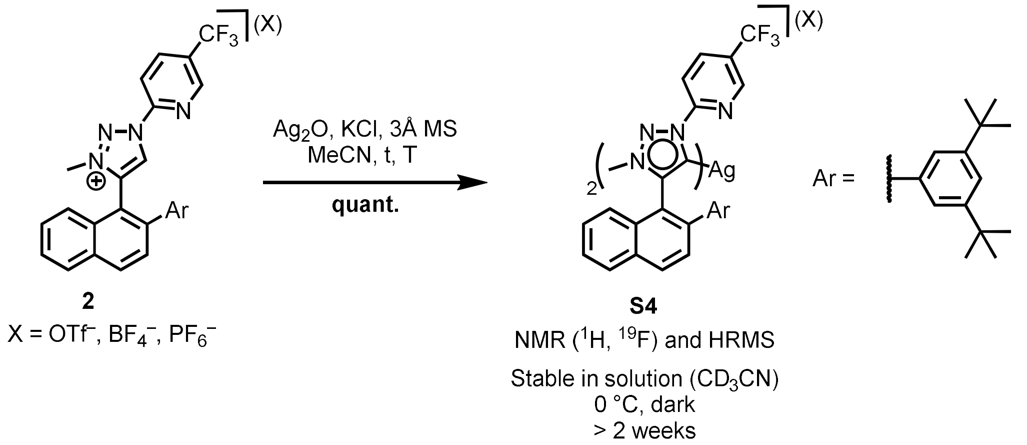

Entry	Temp [°C]	Counterion X⁻	Full conversion after[a]	Significant decomposition observed[a]	Isolated yield
1	80	OTf	30 min	no[d]	n.d.
2	60	OTf	16 h[b]	no	quant.
3	r.t.	OTf	19 h[b,c]	no	n.d.
4	r.t.	PF_6	18 h[b]	no	quant.
5	r.t.	BF_4	18 h[b]	no	quant.

[a] Evaluated via ^{1}H and ^{19}F NMR in CD_3CN at 300K. [b] Conversion was only probed at the indicated times. Full conversion is assumed to have occurred earlier (according to footnote c). [c] Already after 8 h a ratio ligand/Ag-carbene of 1:11 was observed. [d] Prolonged heating overnight revealed slight decomposition to ligand.

Characterisation of Ag-carbene S4

^{1}**H NMR** (300 MHz, CD_3CN): δ (ppm) = 8.30-8.01 (m, 9H), 7.78-7.75 (m, 2H), 7.74-7.72 (m, 1H), 7.70-7.64 (m, 2H), 7.56 (t, *J* = 7.4 Hz, 2H), 7.51-7.45 (m, 1H), 7.29 (d, *J* = 6.4 Hz, 2H), 7.25-7.19 (m, 1H) 7.10 (d, *J* = 3.0 Hz, 4H), 3.43-3.42 (m, 6H), 1.09-1.02 (m, 36H). ^{19}**F NMR** (282 MHz, CD_3CN): δ (ppm) = −62.56 (s, CF_3), −62.76 (s, CF_3), –72.98 (d, $J_{P,F}$ = 706.14 Hz, PF_6). **HRMS** ESI (+); *m/z* calcd. for: $C_{66}H_{66}AgF_6N_8H$ $[M]^+$: 1193.4367, found: 1193.4344.

Ag-carbene **S4** gives the same spectral data regardless of the anion OTf^-, BF_4^- or PF_6^-.

Remarks regarding the stability of Ag-carbene S4

- Storage in CD_3CN in the dark at 0 °C revealed no sign of decomposition after two weeks (NMR).
- Filtration over silica gives decomposition to the ligand starting material.
- Usage of non-dry solvents for the workup of Ag-carbene **S4** had to negative impact.

Optimisation of conditions for transmetalation.

The Ag-carbene **S4** was synthesised according to the procedure above at room temperature with a reaction time between 16 h and 24 h.

The obtained filtrate containing the Ag-carbene **S4** was transferred to an oven dried SCHLENK tube and the solvents removed under vacuum and freshly activated 3 Å molecular sieves (7.3 mg, 1.0 g/ mmol of **2**) were added. Then, a freshly prepared stock solution of AgX in MeCN (0.65 ml MeCN, 0.011 *M* with respect to ligand **2**) was added. (Because the AgX salts are very hygroscopic a special procedure had to be employed in order to minimize water-contamination. A dried and pre-weighed flask containing nitrogen was quickly charged with a spatula tip of AgX. Then, the flask was evacuated on high vacuum for at least 5 h. Subsequent flushing with nitrogen and weighing allowed to determine added amount of AgX. Then, under nitrogen dry MeCN was added to give a solution with appropriate concentration that upon using as solvent for the reaction, contained the respective equivalents of AgX with respect to the Ag-carbene intermediate). Then, the mixture was put to the respective temperature, immediately followed by addition of $FeCl_2$ (0.46 mg, 3.6 µmol, 0.50 equiv.) and further stirred at the respective temperature for the indicated time. After the indicated time, an aliquot was taken under positive nitrogen pressure via syringe. The aliquot was diluted with MeCN, filtered over Celite and the solvents removed at a temperature not higher than the conducted reaction. The residues were dispersed in 20:1 CH_2Cl_2/MeCN, filtered over Celite and solvents of the filtrate were removed as described above. Afterwards, crude NMR of the aliquot in CD_3CN was measured at room temperature.

Table S2: Survey of amount of added $AgPF_6$.

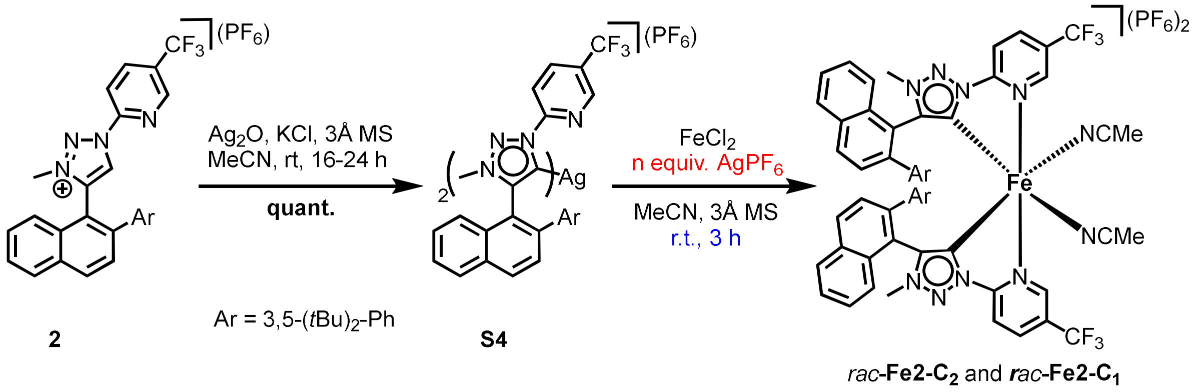

Entry	equiv. $AgPF_6$	Isomer-ratio[a]		
		C_2	C_1	ligand
1	1.0	1	1.6	2
2	2.0	1	0.3	1
3	3.0	1	traces	1.3
4	5.0	0	0	1

[a] Relative ratio of representative ^{19}F NMR signals after workup of the crude.

Table S3: Survey of influence of different temperatures as a function of added $AgPF_6$ amount.

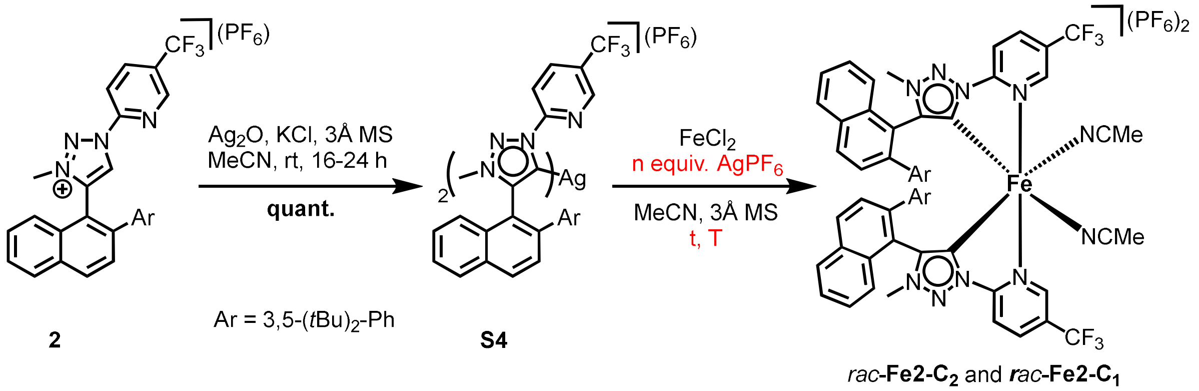

Entry	equiv. $AgPF_6$	t [°C]	T	Conversion of carbene	Isomer-ratio[a]		
					C_2	C_1	ligand
1	2.0	0	5.5 h	none	-	-	n.d.
		→ rt	5.5 h	not complete	1	0.15	0.6
			→ 14 h	not complete	1	0.15	0.6
2	2.0	50	20 min[b]	quant.	1	1.4	0.8
3	3.0	35 → 55[c]	18 h[d]	quant.	1	0.15	6.6
4	5.0	35 → 55[c]	18 h[d]	quant.	1	0.08	22

[a] Relative ratio of representative ^{19}F NMR signals after workup of the crude. [b] Further heating for 20 h gave no change of ratio. [c] Heating increased in 5 °C increments every hour until 55 °C was reached, significant conversion of carbene was observed over time. [d] Heating occurred overnight until full conversion was ensured. This had no influence on the reported isomer ratio.

Table S4: Survey of influence of decreased temperature toward kinetic control and conversion comparing $AgPF_6$ and $AgBF_4$.

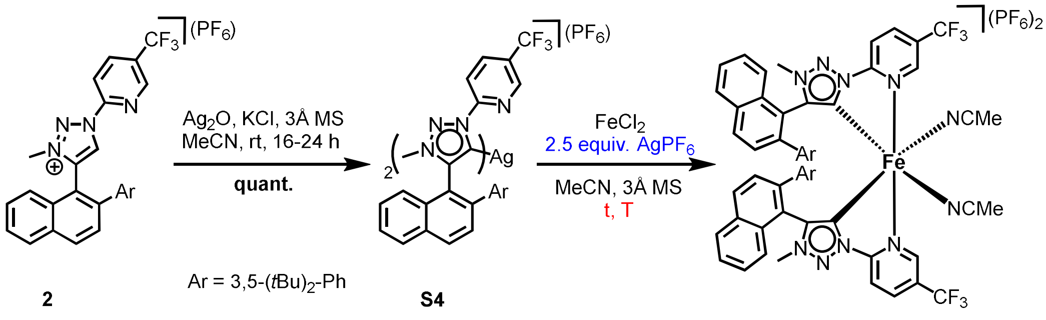

2 S4 *rac*-**Fe2-C**$_2$ and *rac*-**Fe2-C**$_1$

Entry	X^-	t [°C]	T	Conversion of carbene	Isomer-ratio[a]		
					C_2	C_1	ligand
1	PF_6	35	3 h	not complete	1	0.28	0.4
2	PF_6	0 → r.t.	1 h → 18 h	not complete	1	0.15	0.4
3	BF_4	35	1 h	quant.	1	1.8	1.2
4	BF_4	0[b] → r.t.	1 h → 25 h	quant.	1	1	0.3
5	BF_4	−40°C → r.t.	15 h → 2d	quant.	1	0.1	0.3

[a] Relative ratio of representative ^{19}F NMR signals after workup of the crude. [b] At this temperature conversion was already deducible by characteristic colour change of the reaction to dark red (not the case for Entry 2).

In Summary, the above experiments led to the following conclusions:

- With increased amount of AgX (Table S2)
  - The C_2-isomer is favoured.
  - Decomposition rate of carbene ligand decreases slightly.
  - Too large amounts of AgX lead to decomposition.
- In presence of AgX, the ratio in favour of the C_2-isomer further depends on the rate of reaction, which can be controlled by temperature (Table S3, Table S4).
  - Lowering temperature decreases reaction rate.
  - Too low temperature (< 0 °C) does not allow conversion; a temperature gradient is required. Starting at low temp. for C_2-favour, increasing for conversion.
  - After starting reaction at low temperatures, further temperature increase does not alter the isomer-ratio.
  - Too high temperature (> 35 °C) in the presence of AgX leads to increased decomposition to the free ligand (the isolated Fe-complex remains stable up to 50 °C in MeCN: General procedure F).
- Addition of $AgBF_4$ gives an increased reaction rate compared to $AgPF_6$ (Table S4).
  - Reaction-initiation below 0 °C and less steep temperature gradient becomes possible to increase kinetic favour of C_2 and yield respectively.

These conclusions led to transmetalation method B described below and in Table 1 (Main Manuscript).

3.2 Synthesis of *rac*-Fe1 and *rac*-Fe2

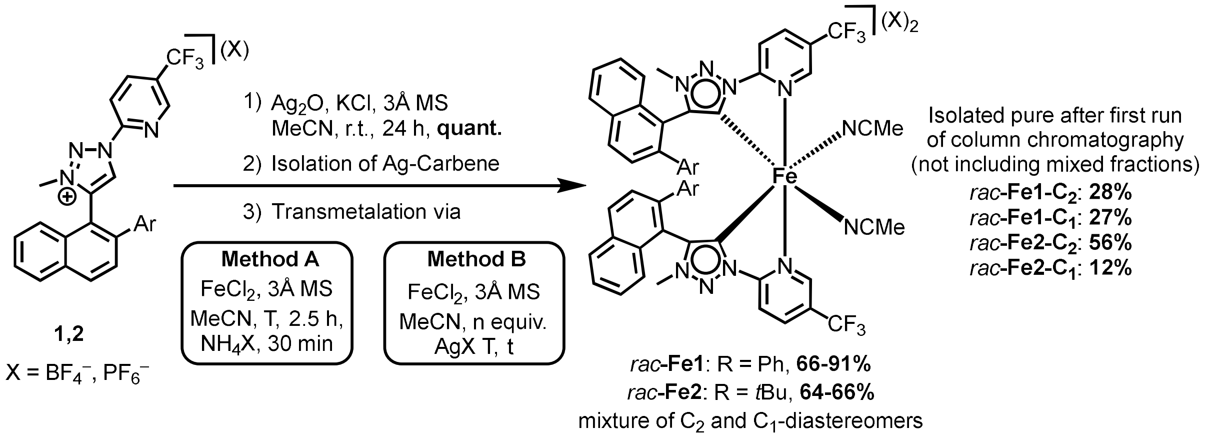

General procedure D: Synthesis of MIC-Fe(II)-complexes *rac*-Fe1 and *rac*-Fe2

Optimised synthesis of the Ag-carbene-intermediate

In an oven dried SCHLENK tube 3 Å molecular sieves (1.0 g/mmol of **1**-X or **2**-X, X = BF_4^-, PF_6^-) was freshly activated by heating at 250 °C for 30 min under vacuum. After cooling to room temperature, the tube was charged with ligand **1**-X or **2**-X (2.05 equiv.), KCl (20.0 equiv.) and Ag_2O (7.0 equiv.), evacuated and flushed with nitrogen. Subsequently the solids were dispersed in dry MeCN (0.011 *M* with respect to 1.0 equiv.) and stirred for 24 h at room temperature under exclusion of light. After completion, the mixture was filtered over a pad of Celite and rinsed with MeCN. The solvents were removed under reduced pressure on a rotary evaporator with a water bath at 30 °C. Then, the residues were dispersed in CH_2Cl_2 and filtered over a pad of Celite to remove insoluble salts. The material was dried on high vacuum and directly used for the next step.

Transmetalation Method A

The Ag-carbene from the step above was transferred quantitatively into an oven dried SCHLENK tube. Then, activated 3 Å molecular sieves (1.0 g/mmol of **1** or **2,** X = BF_4^-, PF_6^-) and $FeCl_2$ (1.00 equiv.) were added. After evacuating and flushing the vessel with nitrogen, dry MeCN (0.011 *M* with respect to 1.00 equiv.) was added at the respective temperature. The mixture was stirred at the indicated temperature for 2.5 h before NH_4X (10.0 equiv.) was added and stirring continued for 30 minutes. After completion the mixture was filtered over Celite, rinsed with MeCN and the solvent removed under reduced pressure. The residues were dispersed in 20:1 CH_2Cl_2/MeCN, filtered over Celite and solvents of the filtrate removed under reduced pressure.

Transmetalation Method B

The Ag-carbene from the step above was transferred quantitatively into an oven dried SCHLENK tube. Then, activated 3 Å molecular sieves (1.0 g/mmol of **1** or **2,** X = BF_4^-, PF_6^-) and $FeCl_2$ (1.00 equiv.) were

added. After evacuating and flushing the vessel with nitrogen, a freshly prepared stock solution of AgX in MeCN (MeCN 0.011 *M* with respect to 1.00 equiv.) was added at the indicated temperature. (Because the AgX salts are very hygroscopic a special procedure had to be employed in order to minimize water-contamination. A dried and pre-weighed flask containing nitrogen was quickly charged with a spatula tip of AgX. Then, the flask was evacuated on high vacuum for at least 5 h. Subsequent flushing with nitrogen and weighing allowed to determine added amount of AgX. Then, under nitrogen dry MeCN was added to give a solution with appropriate concentration that upon using as solvent for the reaction, contained the respective equivalents of AgX *with respect to the Ag-carbene intermediate*). The mixture was stirred at the indicated temperature and time. After completion the mixture was filtered over Celite, rinsed with MeCN and the solvent removed under reduced pressure. The residues were dispersed in 20:1 CH_2Cl_2/MeCN, filtered over Celite and solvents of the filtrate removed under reduced pressure.

Purification via silica gel column chromatography

A column was packed with silica which was dispersed in CH_2Cl_2/MeCN (X = PF_6: 20:1, X = BF_4: 10:1). The surface of the silica was covered with NH_4X, the crude was dissolved in minimal amount of eluent and applied onto the stationary phase. Slow elution via stepwise increase of eluent polarity (CH_2Cl_2/MeCN) facilitated separation of the diastereomers *rac*-**Fe1-C$_2$** and *rac*-**Fe1-C$_1$** or *rac*-**Fe2-C$_2$** and *rac*-**Fe2-C$_1$.**

Note 1: The typical column conditions involved using approximately 250 mL of silica per 100 mg of crude material in a column with a 3.5 cm diameter. Pressurized air was applied during chromatography.

Note 2: The low yields of the isomers only refer to isolated *pure* material after a first run of column chromatography of the crude. Obtained mixed fractions could then further be purified by another chromatography run. The complexes showed no significant decomposition even when subjected to silica column chromatography for several hours and several times.

3.3 Remarks for Table 1

Additional entries for synthesis of iron complexes (Ar = Ph)

Table S5: Reaction condition for the synthesis of *rac*-**Fe1**

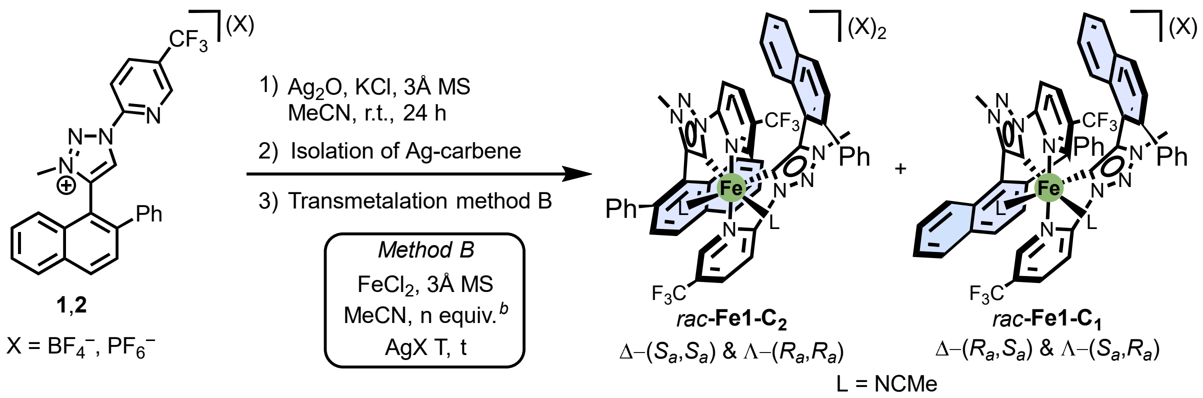

Entry	n equiv. AgX	T (°C)	t (h)	Isomer-ratio[a]		Yield (%)	X^-
				C_2	C_1		
S1	0[g]	4	3	1	2.6	87	PF_6
S2	0[g]	−40 °C → r.t.[e]	4.5[e]	1	2.9	61[f]	PF_6
S3	2.5	r.t.	17	1	1	74	PF_6
S4	2.5	r.t.	17	1	1	66	BF_4
S5	2.5	−40 °C → r.t.[e]	110[e]	1	1	76	BF_4

[a] The depiction of the complexes only features Δ-enantiomers; a representation of the possible isomers and their relation can be found in section 12.1 of this document. [b] Referred to the intermediate mono-cationic Ag-carbene dimer. [c] Ratio determined by ^1H and ^{19}F NMR after short workup. [d] Yields determined from crude NMR and absolute weight of the crude after short workup. Free ligand was calculated out by factoring the crude NMR ratios and the respective molecular masses. [e] For a detailed gradual warming protocol see below. [f] Yield after silica column chromatography to ensure full removal of unreacted traces of the Ag-carbene intermediate. [g] NH_4PF_6 (10 equiv.) was added after completion and stirred for 5 minutes before workup.

Workup procedure to determine crude NMR isomer ratio (footnote c)

The crude reaction mixture was filtered over a plug of Celite, washed with MeCN and the solvents were removed under reduced pressure. The crude was redissolved in CH_2Cl_2/MeCN 20:1 (v:v), filtered, the solvents removed under reduced pressure and analysed by NMR in CD_3CN.

Gradual warming protocol (footnote e)

In presence of the AgX-additive and reaction start at low temperatures, the reaction times had to be increased significantly in order to achieve near full conversion:

Entry 3: −40 °C for 16 h → slowly reach 4 °C (over course of 2 h) stir there for 5.5 h → r.t. for 46 h.

Entry 4: 0 °C for 1 h → r.t. for 16 h.

Entry S4: −40 °C for 1.5 h → slowly reach −25 °C (over course of 1 h) → 4 °C for 2 h.

Entry S5: −40 °C for 16 h → slowly reach 4 °C (over course of 2 h) stir there for 66 h → rt for 27 h.

4 Auxiliary Complex Synthesis

General procedure E: Auxiliary complex synthesis

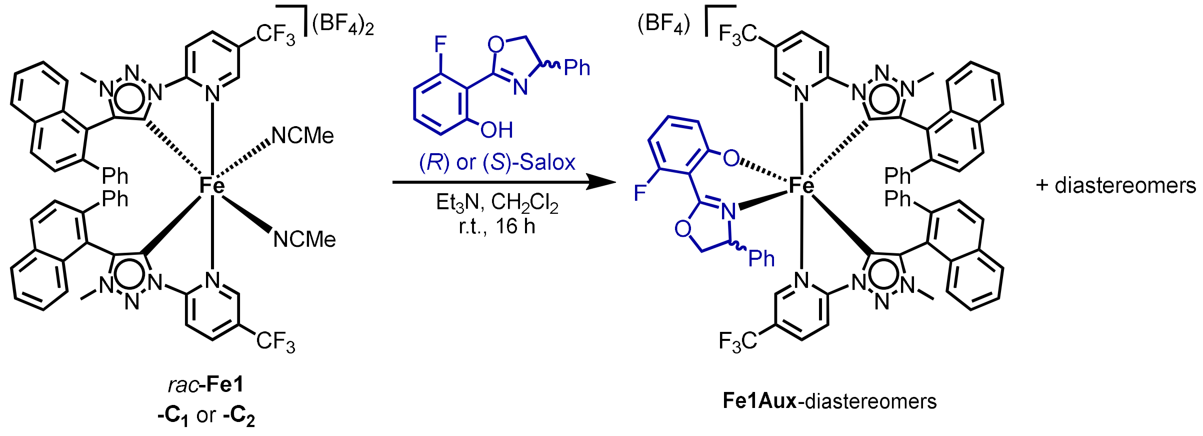

An oven dried SCHLENK tube was charged with *rac*-**Fe1-C₁** or *rac*-**Fe1-C₂** (40 mg, 1.00 equiv.) and (*S*)- or (*R*)- Salox (9.22 mg, 1.05 equiv.), evacuated and flushed with nitrogen. Then, the solids were dissolved in dry CH_2Cl_2 (1.40 mL, 0.025 *M*) and dry Et_3N was added (7.1 µL, 1.50 equiv.). The mixture was stirred at room temperature for 16 h, before it was filtered over Celite and rinsed with CH_2Cl_2. The solvents were removed under reduced pressure. The obtained crude was purified by flash-column chromatography (silica gel, CH_2Cl_2/MeCN 25:1 → 5:1).

For *rac*-**Fe1-C₂**: Following the procedure above, the desired auxiliary complexes Δ-(S_a,S_a)-(*R*)-**Fe1Aux** or Λ-(R_a,R_a)-(*S*)-**Fe1Aux** were obtained. The respective unstable auxiliary complexes Λ-(R_a,R_a)-(*R*)-**Fe1Aux** or Δ-(S_a,S_a)-(*S*)-**Fe1Aux** decomposed during the procedure but could be eluted (NH_4BF_4-addition on column, CH_2Cl_2/MeCN 5:1 → 1:2) as the enantioenriched MeCN complexes Λ-(R_a,R_a)-**Fe1-C₂** or Δ-(S_a,S_a)-**Fe1-C₂**, which could be further purified by converting with (*S*)- or (*R*)- Salox, respectively.

For *rac*-**Fe1-C₁:** Separation or selective decomposition of the obtained diastereomers from the crude mixture was not feasible.

4.1 Configurational stability of Fe1-C_1

4.1.1 Diastereomeric Mixture in Auxiliary-Complex Formation

Auxiliary complex-Synthesis

Method: Coordination of the chiral auxiliary (*R*)-Salox to *rac*-**Fe1-C₁** was used to obtain the respective diastereomeric auxiliary complexes **Fe1Aux** and attempted to be separated. The diastereomeric ratio and yield were determined by ^{19}F NMR.

Procedure: Following general procedure E, *rac*-**Fe1-C_1** (10.0 mg, 8.5 µmol, 1.00 equiv.) was reacted with (*R*)-Salox at room temperature for 16 h. The solvents were removed under vacuum and the crude was dissolved in CD_2Cl_2, then 3,3,3-trifluoropropan-1-ol (2.00 equiv.) as internal standard was added and the solution analysed by ^{19}F NMR.

Observation: A crude yield of 99% was determined via the internal standard (green dots) and two diastereomers with dr = 3:1 (blue/red dots) were obtained (Figure S1a). Isolation by flash column chromatography (silica, CH_2Cl_2/MeCN) yielded the mixture of diastereomers (10.4 mg, 8.3 µmol, 97%) while retaining the dr = 3:1 (Figure S1b).

Result: Following NMR-analysis and assignment of the diastereomeric mixture by NMR (see below) we conclude, that they correspond to Δ-(S_a,R_a)-(*R*)-**Fe1Aux-a** (blue dots) and Δ-(S_a,R_a)-(*R*)-**Fe1Aux-b** (red dots) respectively. This indicates a deracemization and underlines the configurational instability of **Fe1-C_1.**

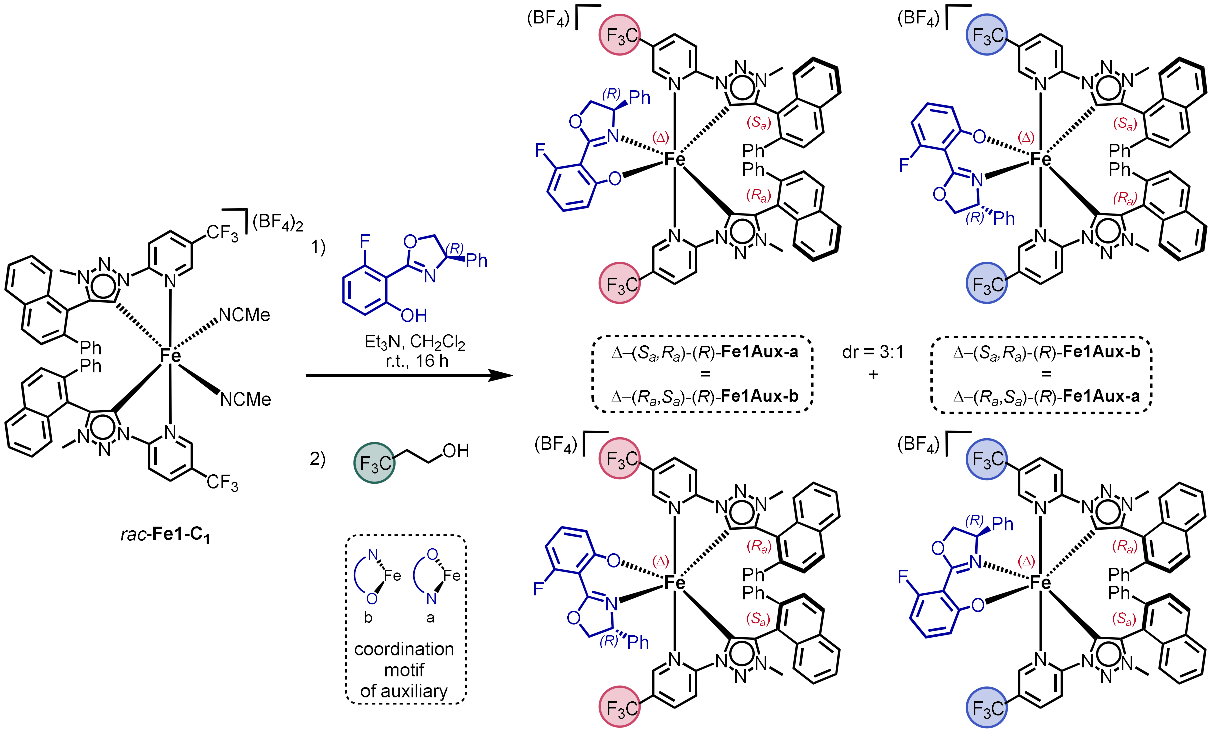

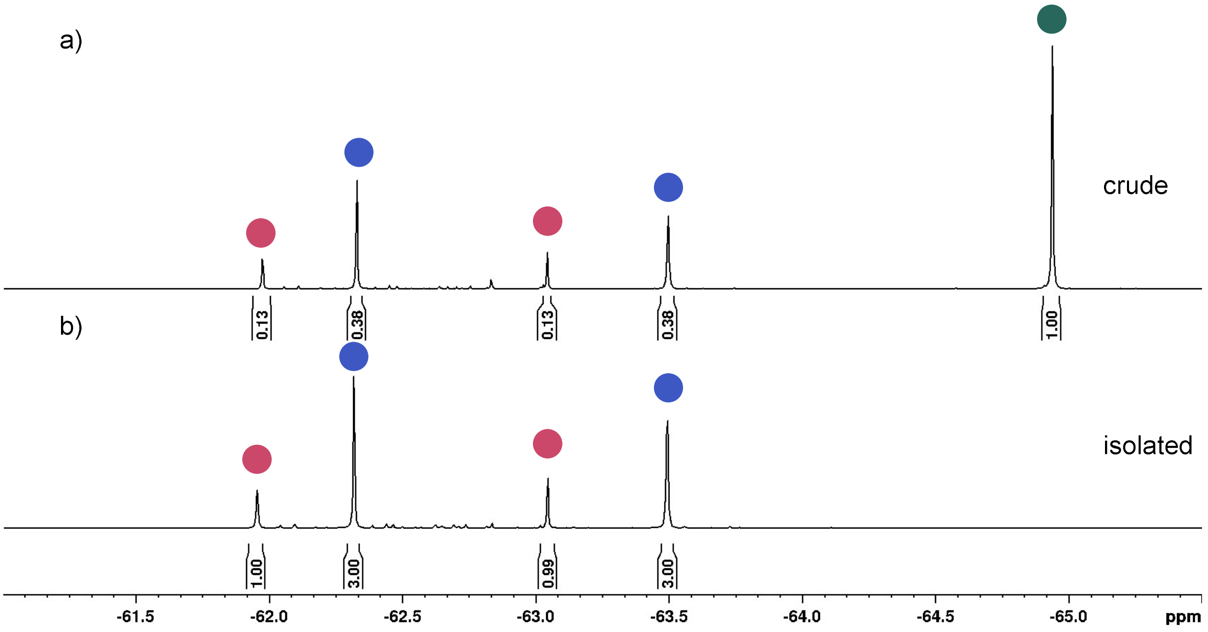

Figure S1: ^{19}F NMR (CD_2Cl_2, 300 K) spectra. a) Crude with 3,3,3-trifluoropropan-1-ol as internal standard (green). b) After column chromatography.

4.1.2 NMR Studies on Diastereomeric Mixture

Method: The obtained diastereomeric mixture containing Δ-(S_a,R_a)-(*R*)-**Fe1Aux-a** and Δ-(S_a,R_a)-(*R*)-**Fe1Aux-b** was analysed in detail by NMR spectroscopy in order to assess the respective metal centred configuration and the different coordination motif of the auxiliary for *both* diastereomers.

Procedure: About 10 mg of the substance were dissolved in 0.6 mL of acetone-d_6 under inert atmosphere and degassed via bubbling with argon. Experiments were performed on a Bruker NEO 600 MHz spectrometer equipped with a 5 mm iTBO probe with z-gradient. Two-dimensional correlation spectra of ^{1}H, ^{1}H DQF-COSY, ^{1}H, ^{13}C HSQC and HMBC were recorded with standard pulse programs.[13] Chemical shifts were referenced to the residual solvent signal.

Results and Discussion

Figure S2 displays the ^{1}H spectrum of a mixture of Δ-(S_a,R_a)-(*R*)-**Fe1Aux-a** and Δ-(S_a,R_a)-(*R*)-**Fe1Aux-b** diastereomers in acetone-d_6. Signals of aliphatic protons in the region 3.3–5.0 ppm are shown in Figure S3. There, pairs of signals with a 3:1 ratio, which corresponds to a molar ratio of 3:1 of the major and minor diastereomers Δ-(S_a,R_a)-(*R*)-**Fe1Aux-a** (filled dots) and Δ-(S_a,R_a)-(*R*)-**Fe1Aux-b** (open dots) can be observed. Additionally, two pairs of singlets at 4.70 and 3.36 ppm, 4.21 and 4.11 ppm were observed and assigned to the NCH_3 of the coordinated MIC ligands, respectively. Furthermore, a pair of double doublets was detected at 4.85 and 3.38 ppm and assigned to CH-9^{Aux} of the chiral Salox-ligand. Finally, two pairs of double doublets were observed at 4.39 and 4.10 ppm, 4.40 and 3.76 ppm, which were assigned to the two diastereotopic protons of CH_2-10^{Aux}, respectively. The above assignments were verified by two-dimensional DQF-COSY and HSQC spectra (Figures S7 and Figure S8).

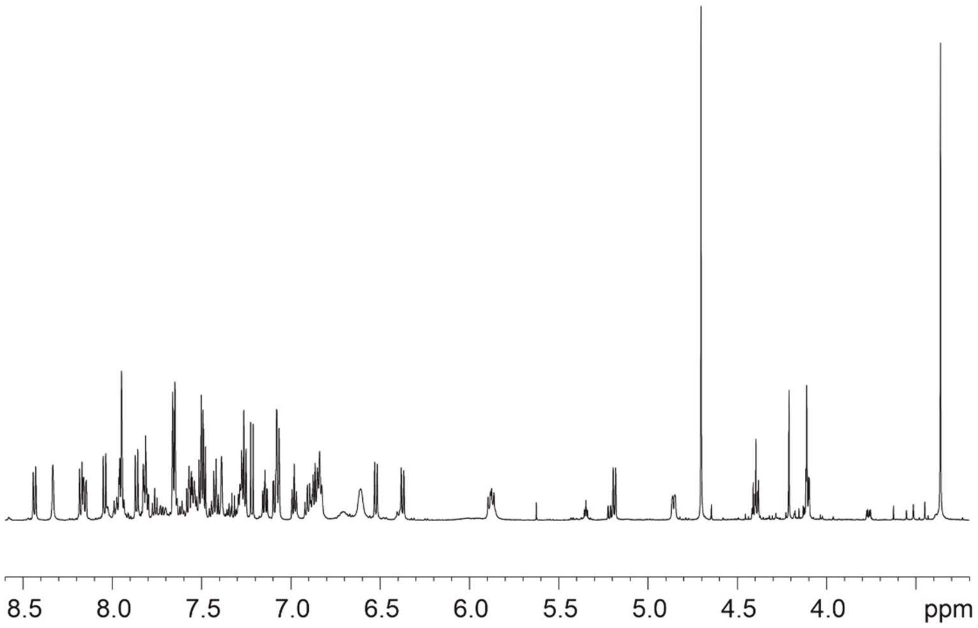

Figure S2: ^{1}H NMR spectrum of the Δ-(S_a,R_a)-(R)-**Fe1Aux-a** and Δ-(S_a,R_a)-(R)-**Fe1Aux-b b** diastereomer-mixture in acetone-d_6 at 298 K.

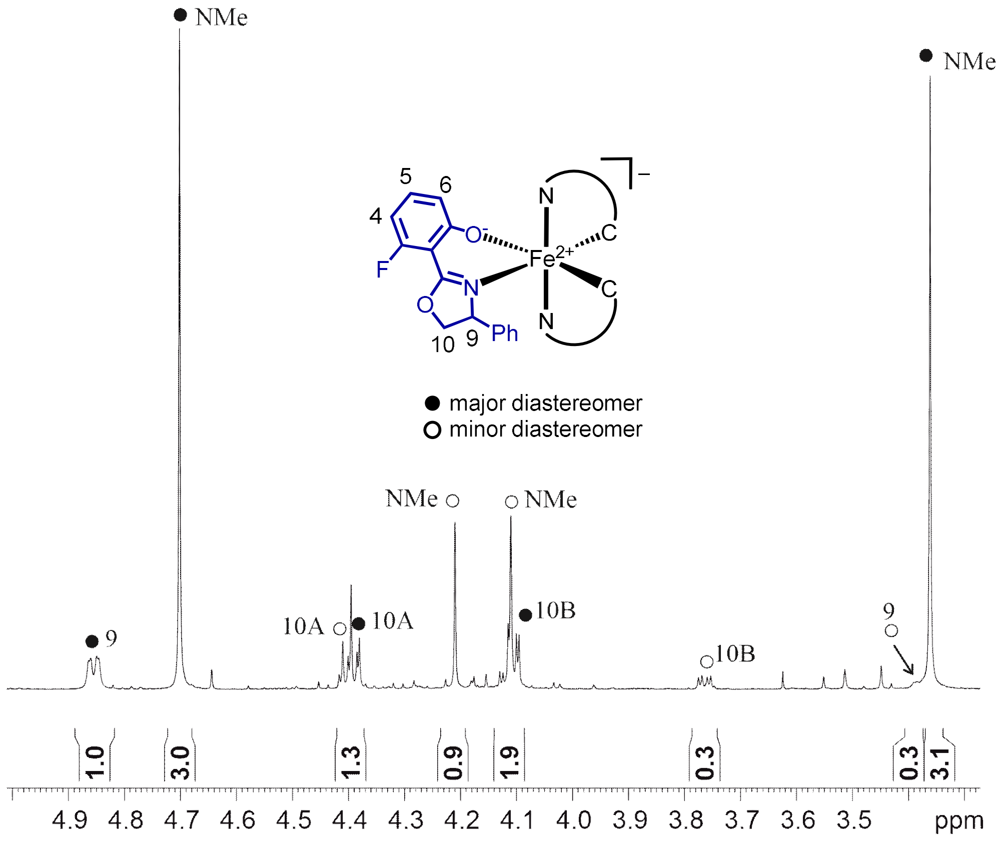

Figure S3: ^{1}H NMR spectrum in the region 3.3–5.0 ppm for aliphatic protons of the Δ-(S_a,R_a)-(R)-**Fe1Aux-a** and Δ-(S_a,R_a)-(R)-**Fe1Aux-b** diastereomer-mixture in acetone-d_6 at 298 K. The stereochemistry of the depicted structure is arbitrary.

A close inspection of the change in chemical shift of H-9^{Aux} (and H-$10B^{Aux}$ as well) between the major and minor diastereomers revealed an upfield shielding of 1.5 ppm (H-9^{Aux}) in the minor diastereomer. This shielding is caused by the ring current of an adjacent naphthalene group in the complex. The ring current induced by the delocalized π-electrons in a magnetic field can cause strong shielding to atoms

located above and below the plane. We already successfully made use of the ring current effect in the stereo chemistry determination of chiral-at-ruthenium phenanthroline complexes.[14]

3D-structures of Δ-(S_a,R_a)-(R)-**Fe1Aux-a** and Δ-(S_a,R_a)-(R)-**Fe1Aux-b** diastereomers are presented in Figure S4. Only the Δ-configured isomers can fulfil the ring current effect on H-9^{Aux}. In order to model the respective Λ-enantiomers, only the stereocenter of the auxiliary ligand needs to be inverted. This would bring H-9^{Aux} away from the naphthalene ring current effect, while also suggesting steric clash of the auxiliary-phenyl with naphthalene ring. Thus the diastereomers were assigned to Δ–*(S_a,R_a)-(R)*-**Fe1Aux-a** and Δ–*(S_a,R_a)-(R)*-**Fe1Aux-b**, respectively.

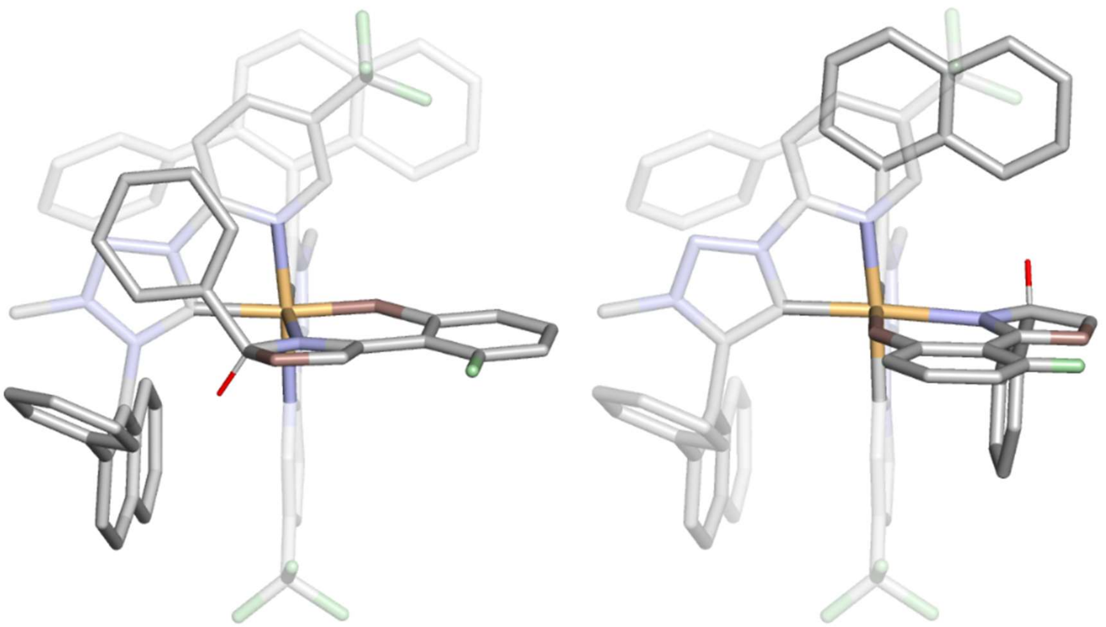

Figure S4: 3D structures of Δ–*(S_a,R_a)-(R)*-**Fe1Aux-a** (left) and Δ–*(S_a,R_a)-(R)*-**Fe1Aux-b** (right) with highlight on H-9^{Aux} (dark red) and the adjacent naphthalene plane. Grey: carbon, blue: nitrogen, pale red: oxygen, green: fluorine, yellow: iron. Hydrogens and two counterions are omitted for clarity. The models were drawn with PyMol derived from the crystal-structure of *rac*-**Fe1-C$_1$**. No energy minimisation was calculated.

Figure S5 shows the ^{1}H spectrum in the region 8.5–5.0 ppm for the aromatic protons of the Δ–*(S_a,R_a)-(R)*-**Fe1Aux-a** and Δ–*(S_a,R_a)-(R)*-**Fe1Aux-b** diastereomer-mixture. Signals of those protons in the first-coordination sphere are unambiguously assigned. Pairs of signals for the major diastereomer were observed for H-8, H-10 and H-11 of ligands **1$_a$** (green) and **1$_b$** (red) respectively. Upfield shielding of 1.0, 1.1 and 1.9 ppm was observed for H-8 (green), H-10 (red) and H-11 (red), respectively. Those shielding were caused by the ring current in naphthalene and agree well with the corresponding position in the 3D structure (see Figure S6 for H-11 and H-10 of **1$_b$**). The signal assignment was fulfilled and verified

by the 2D spectra DQF-COSY and HMBC. Therefore the major diastereomer was assigned to Δ–*(S_a,R_a)-(R)*-**Fe1Aux-a** and the minor diastereomer to Δ–*(S_a,R_a)-(R)*-**Fe1Aux-b**, respectively.

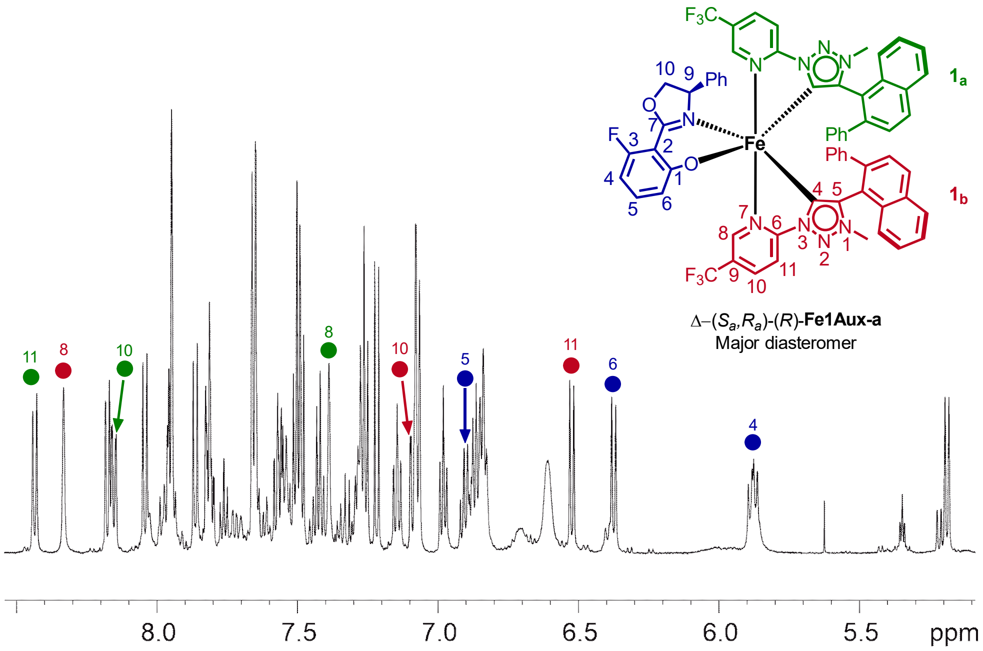

Figure S5: ^{1}H NMR spectrum in the region 5.0–8.5 ppm for aromatic protons of the Δ-(S_a,R_a)-(*R*)-**Fe1Aux-a** and Δ-(S_a,R_a)-(*R*)-**Fe1Aux-b** diastereomer-mixture with highlight on the major Δ-(S_a,R_a)-(*R*)-**Fe1Aux-a** in acetone-d_6 at 298 K.

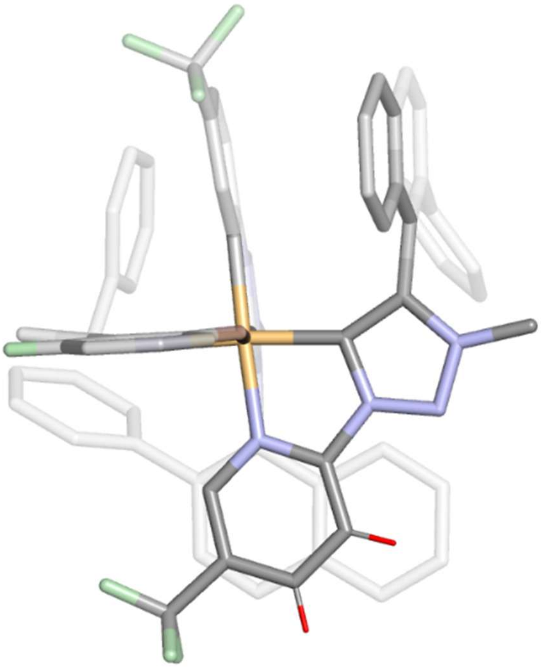

Figure S6: 3D structure of Δ–*(S_a,R_a)-(R)*-**Fe1Aux-a** with highlight on H-10 and H-11 (dark red) above the naphthalene plane. Grey: carbon, blue: nitrogen, pale red: oxygen, green: fluorine, yellow: iron. Hydrogens and two counterions are omitted for clarity. The model was drawn with PyMol derived from the crystal structure of *rac*-**Fe1-C$_1$**. No energy minimisation was calculated.

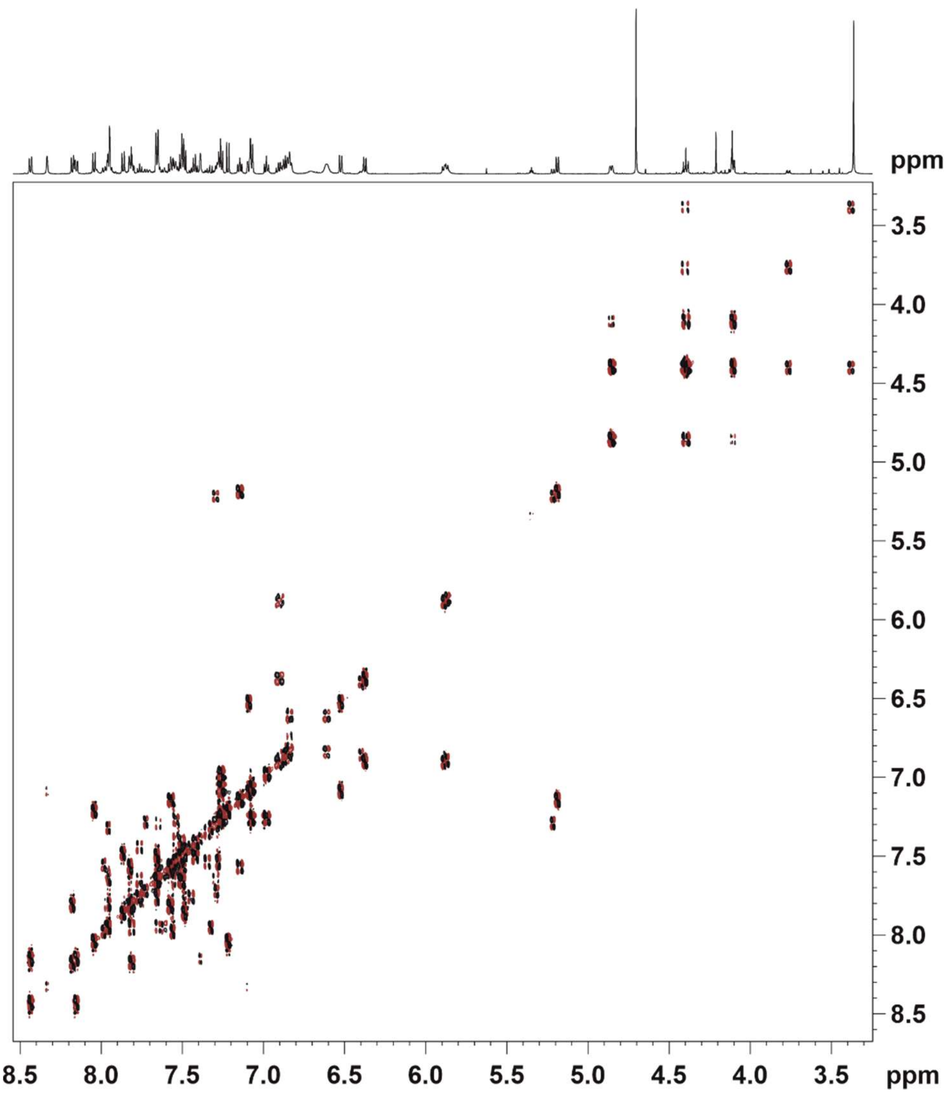

Figure S7: DQF-COSY spectrum of the Δ-(S_a,R_a)-(R)-**Fe1Aux-a** and Δ-(S_a,R_a)-(R)-**Fe1Aux-b**-diastereomer-mixture in acetone-d_6 at 298 K.

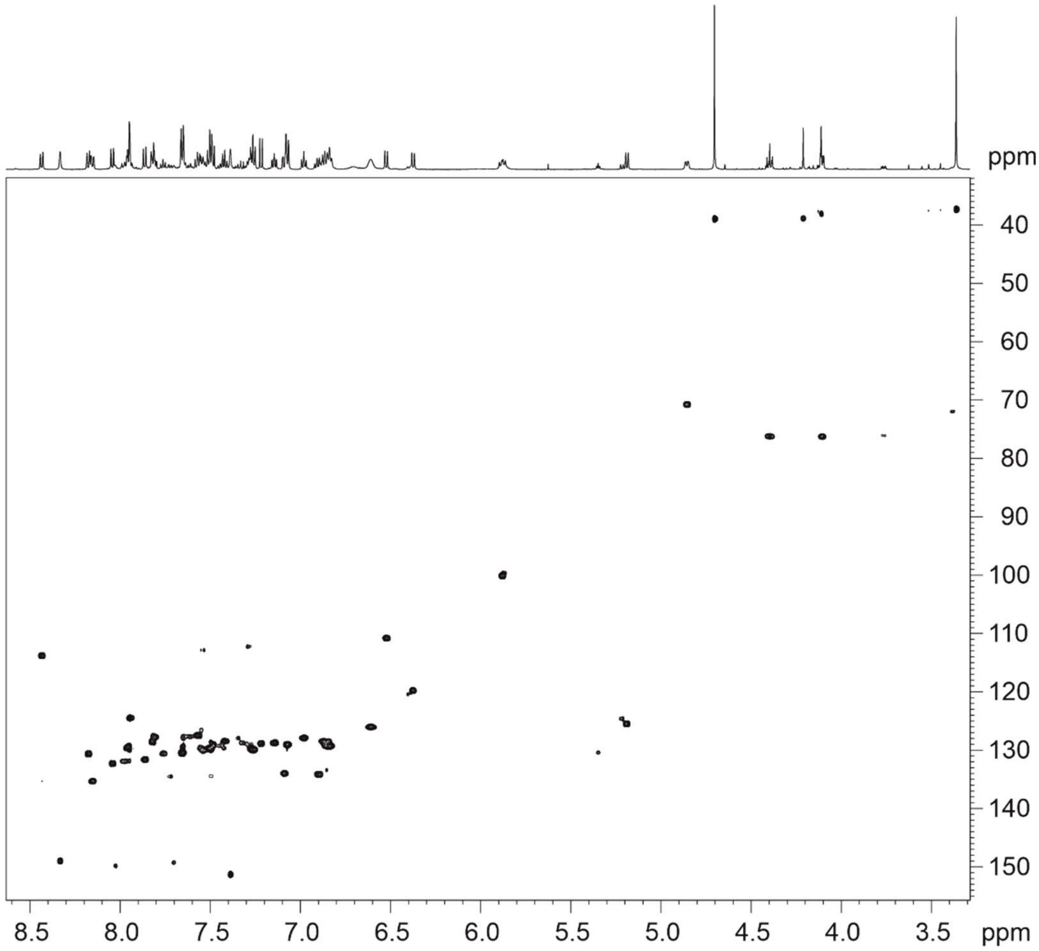

Figure S8: ^{1}H, ^{13}C HSQC spectrum of the Δ-(S_a,R_a)-(R)-**Fe1Aux-a** and Δ-(S_a,R_a)-(R)-**Fe1Aux-b**-diastereomer-mixture in acetone-d_6 at 298 K.

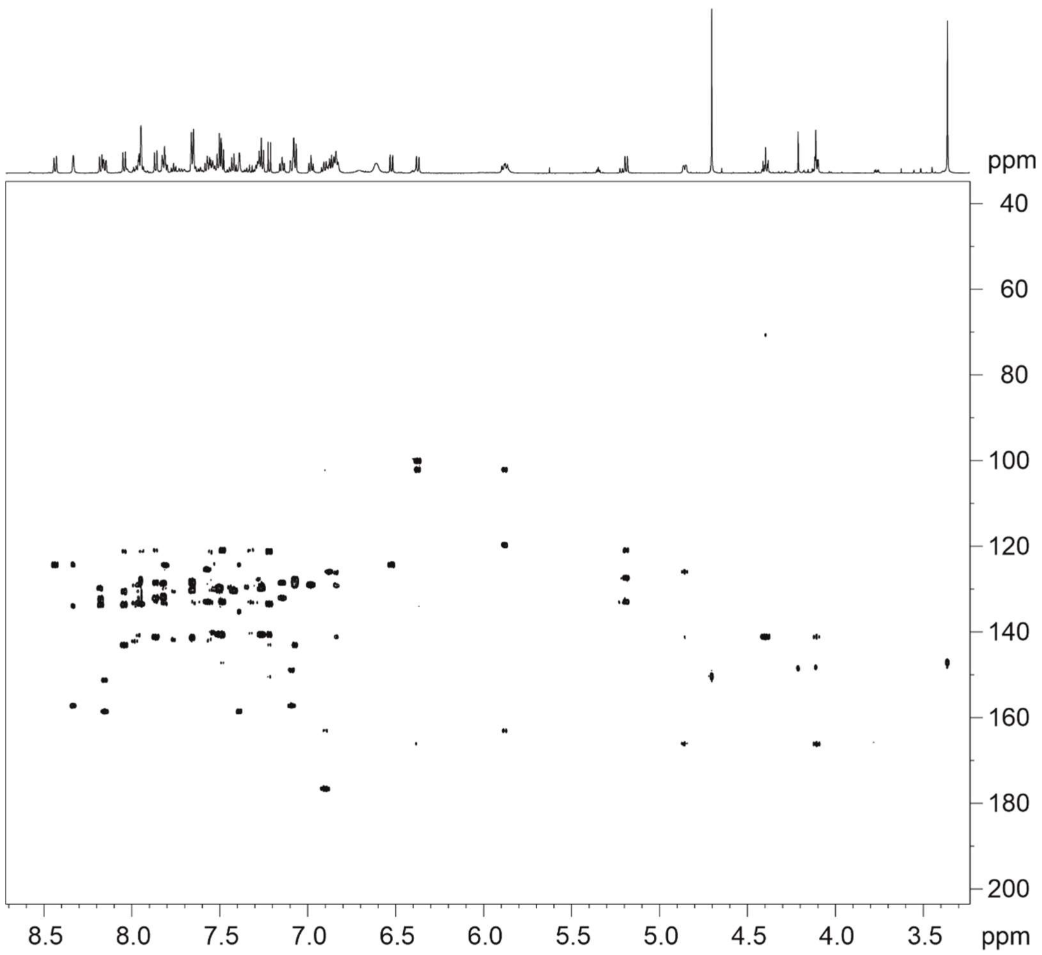

Figure S9: ^{1}H, ^{13}C HMBC spectrum of the Δ-(S_a,R_a)-(R)-**Fe1Aux-a** and Δ-(S_a,R_a)-(R)-**Fe1Aux-b**-diastereomer-mixture in acetone-d_6 at 298 K.

4.2 Configurational Stability of Fe1-C_2

Auxiliary complex-Synthesis

Method: Coordination of the chiral auxiliary (*R*)-Salox to *rac*-**Fe1**-**C_2** was used to obtain the respective diastereomeric auxiliary complexes **Fe1Aux** and attempted to be separated. The diastereomeric ratio was determined by ^{19}F NMR.

Procedure: Following general procedure E, *rac*-**Fe1**-**C_2** (40.0 mg, 34.1 µmol, 1.00 equiv.) was reacted with (*R*)-Salox in the presence of Et_3N at room temperature for 16 h. The solvents were removed under vacuum and the crude was dissolved in CD_2Cl_2 and analysed by ^{19}F NMR. Then, the mixture was treated with silica column chromatography (silica, CH_2Cl_2/MeCN).

Observation: In the crude, two diastereomers (red/blue dots) as well as free ligand (green dot) in a ratio of 2.4 : 2 : 0.4 were obtained (Figure S10a). Column chromatography yielded Δ-(S_a,S_a)-(*R*)-**Fe1Aux** (19.6 mg, 15.6 µmol, 46%) (Figure S10b). alongside an **Fe1**-**C_2** species (Figure S10c).

Result: As after chromatography we only obtain one of the initial auxiliary-complex diastereomers (blue dots) which corresponds to Δ-(S_a,S_a)-(*R*)-**Fe1Aux** in almost quantitative yield (max = 50%) and observe decomposition of the other diastereomer (red dots), we propose that Λ-(R_a,R_a)-(*R*)-**Fe1Aux** is unfavoured and led to the formation of free ligand in the initial crude mixture. Hence, by considering the respective molar ratios in the crude mixture, we calculate an initial dr of 1:1 for Δ-(S_a,S_a)-(*R*)-**Fe1Aux** and Λ-(R_a,R_a)-(*R*)-**Fe1Aux.** Therefore, we conclude that no change in metal centred configuration of *rac*-**Fe1**-**C_2** occurred, marking it configurationally stable under the given conditions.

The same procedure could be applied to the reaction of *rac*-**Fe1**-**C_2** with (*S*)-Salox to give the enantiomer Λ-(R_a,R_a)-(*S*)-**Fe1Aux** (Figure S10d).

Auxiliary complex isolation

The diastereomeric purity after column chromatography of isolated Δ-(S_a,S_a)-(*R*)-**Fe1Aux** or Λ-(R_a,R_a)-(*S*)-**Fe1Aux** respectively was assessed by ^{19}F NMR with 1024 scans. The ^{19}F NMR spectra of isolated Δ-(S_a,S_a)-(*R*)-**Fe1Aux** (Figure S10b). and its enantiomer Λ-(R_a,R_a)-(*S*)-**Fe1Aux** (Figure S10d) show no signals of the respective unstable diastereomers (red dots Figure S10a).

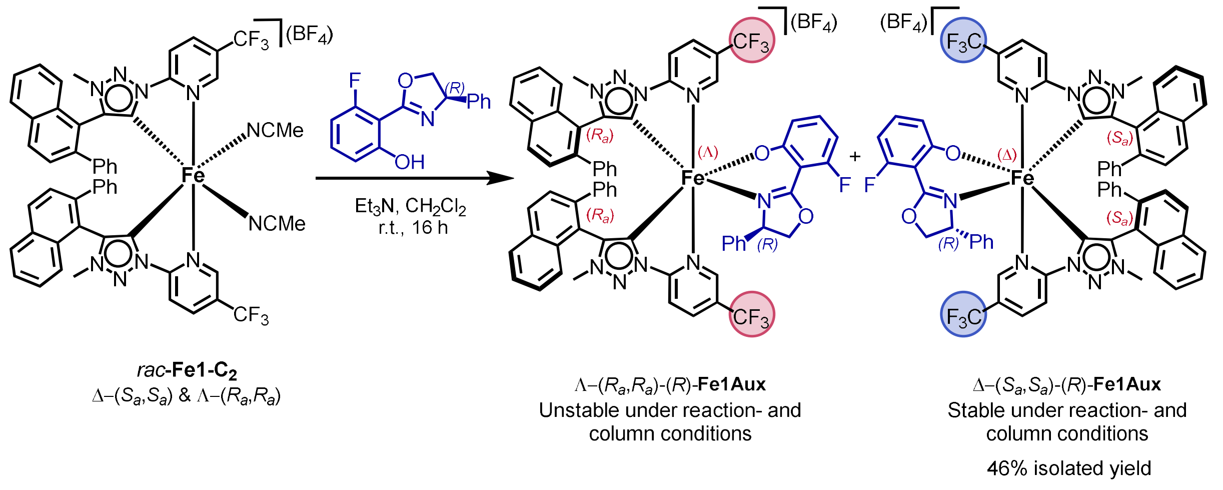

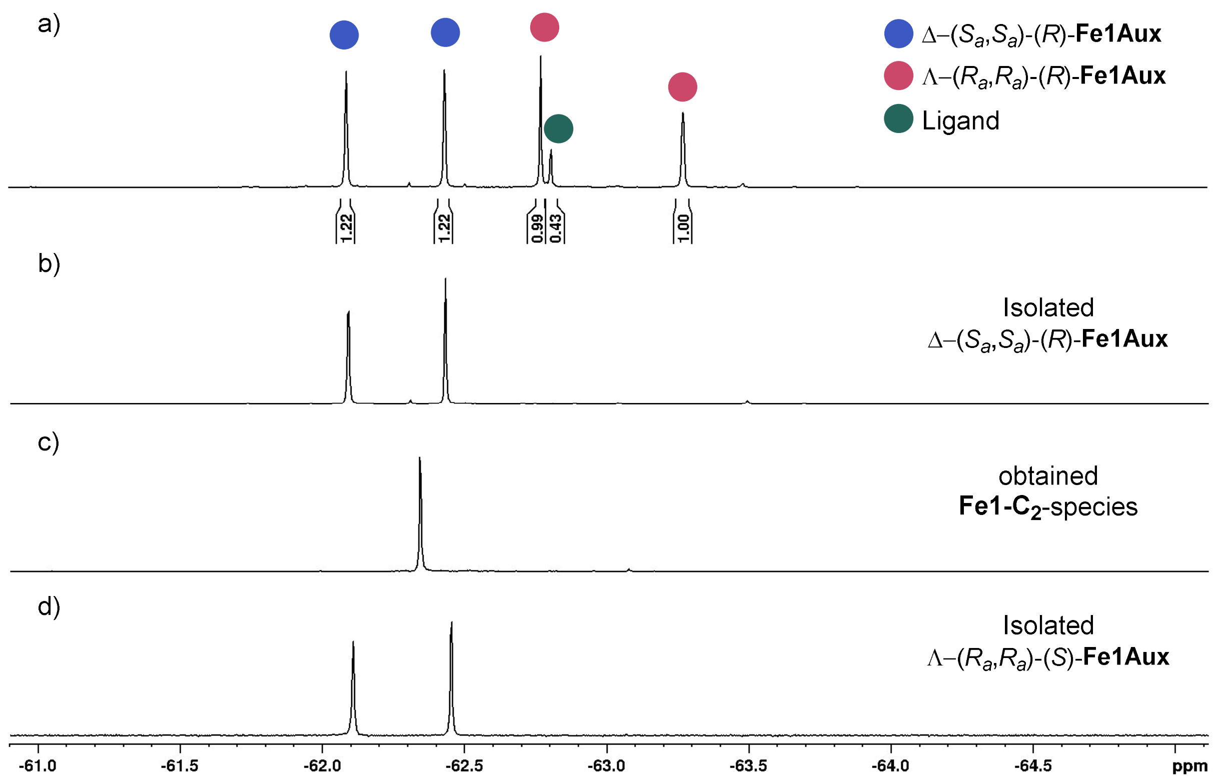

Figure S10: ^{19}F NMR (CD_2Cl_2, 300 K) spectra. a) Crude of the reaction from *rac*-**Fe1-C$_2$** with (*R*)-Salox. b) Isolated Δ-(*S$_a$*,*S$_a$*)-(*R*)-**Fe1Aux**. c) Decomposition of Λ-(*R$_a$*,*R$_a$*)-(*R*)-**Fe1Aux** to **Fe1-C$_2$**. d) Isolated Λ-(*R$_a$*,*R$_a$*)-(*S*)-**Fe1Aux.**

5 Auxiliary Cleavage

General Procedure F: Cleavage of Salox auxiliary

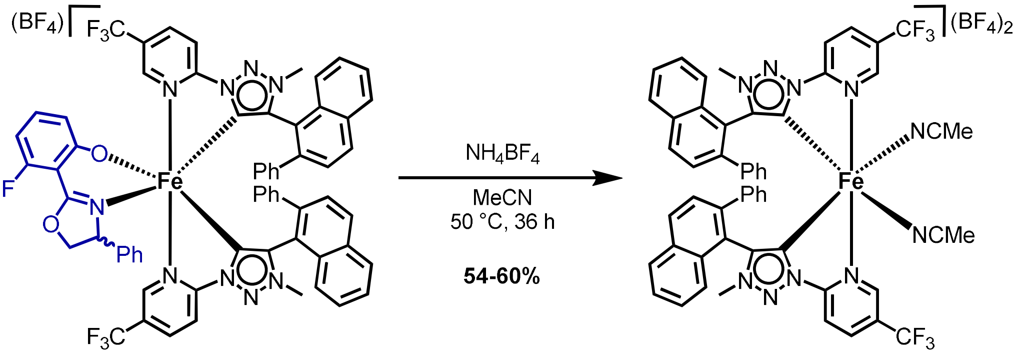

Λ–(R_a,R_a)-(S)-**Fe1Aux**
or
Δ–(S_a,S_a)-(R)-**Fe1Aux**

Λ–(R_a,R_a)-**Fe1-C₂**
or
Δ–(S_a,S_a)-**Fe1-C₂**

An oven dried SCHLENK tube was charged with Δ-(S_a,S_a)-(R)-**Fe1Aux** or Λ-(R_a,R_a)-(S)-**Fe1Aux** (1.00 equiv.) and NH_4BF_4 (10 equiv.) and evacuated and flushed with nitrogen. Then the solids were dissolved in MeCN (0.025 *M*) and heated to 50 °C for 36 h until full conversion was confirmed via ^{19}F NMR. The solvents were removed under reduced pressure and the residues were redissolved in CH_2Cl_2/MeCN 20:1 and filtered over Celite. The solvents from the filtrate were removed under reduced pressure. Upon redissolving in minimal amount of CH_2Cl_2/MeCN 20:1 the MeCN-complexes were precipitated by addition of Et_2O, filtered over Celite and washed with Et_2O. The residues could then be washed down with MeCN to obtain analytically and enantiomerically pure Δ-(S_a,S_a)-**Fe1-C$_2$** or Λ-(R_a,R_a)-**Fe1-C$_2$** as red solid.

Determination of enantiopurity of chiral Δ-(S_a,S_a)-Fe1-C_2

Method: Re-installation of the chiral auxiliary (*R*)-Salox to Δ-(S_a,S_a)-**Fe1-C_2** was used to obtain the respective auxiliary complex Δ-(S_a,S_a)-(*R*)-**Fe1Aux** and analysed by ^{19}F NMR with 1024 scans. Comparison with the crude ^{19}F NMR from the initial coordination to *rac*-**Fe1-C_2** (Figure S11a) was used as a measure for the enantiomeric ratio of purified Δ-(S_a,S_a)-**Fe1-C_2.** (Figure S11b)

Procedure: Following general procedure E, Δ-(S_a,S_a)-**Fe1-C_2** (1.50 mg,12.8 µmol, 1.00 equiv.) was reacted with (*R*)-Salox (0.35 mg, 14.3 µmol,1.05 equiv.) in CD_2Cl_2 (0.4 mL) in presence of Et_3N (0.27 µL, 19.2 µmol, 1.50 equiv.) in an NMR tube at room temperature for 16 hours.

Observation: Only formation of Δ-(S_a,S_a)-(*R*)-**Fe1Aux** (blue dots) and no formation of the diastereomer Λ-(R_a,R_a)-(*R*)-**Fe1Aux** (red dots) was detected (Figure S11b).

Result: We conclude, that Δ-(S_a,S_a)-(*R*)-**Fe1Aux** was obtained with an *e.r.* > 99:1.

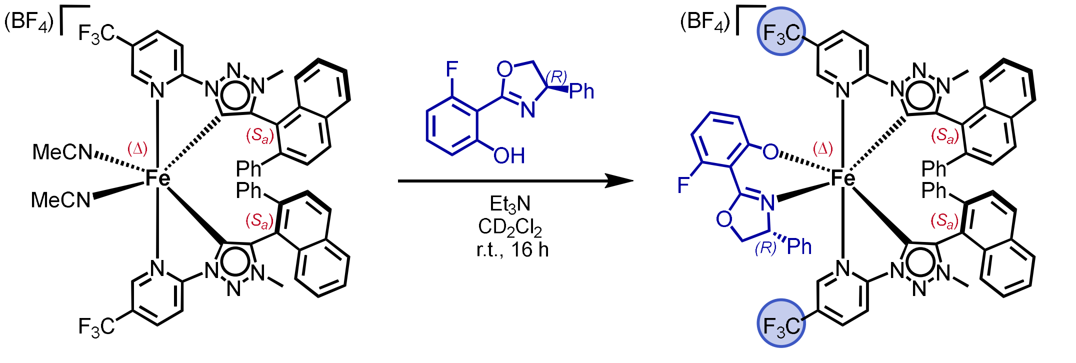

Δ–(S_a,S_a)-**Fe1-C_2** Δ–(S_a,S_a)-(*R*)-**Fe1Aux**

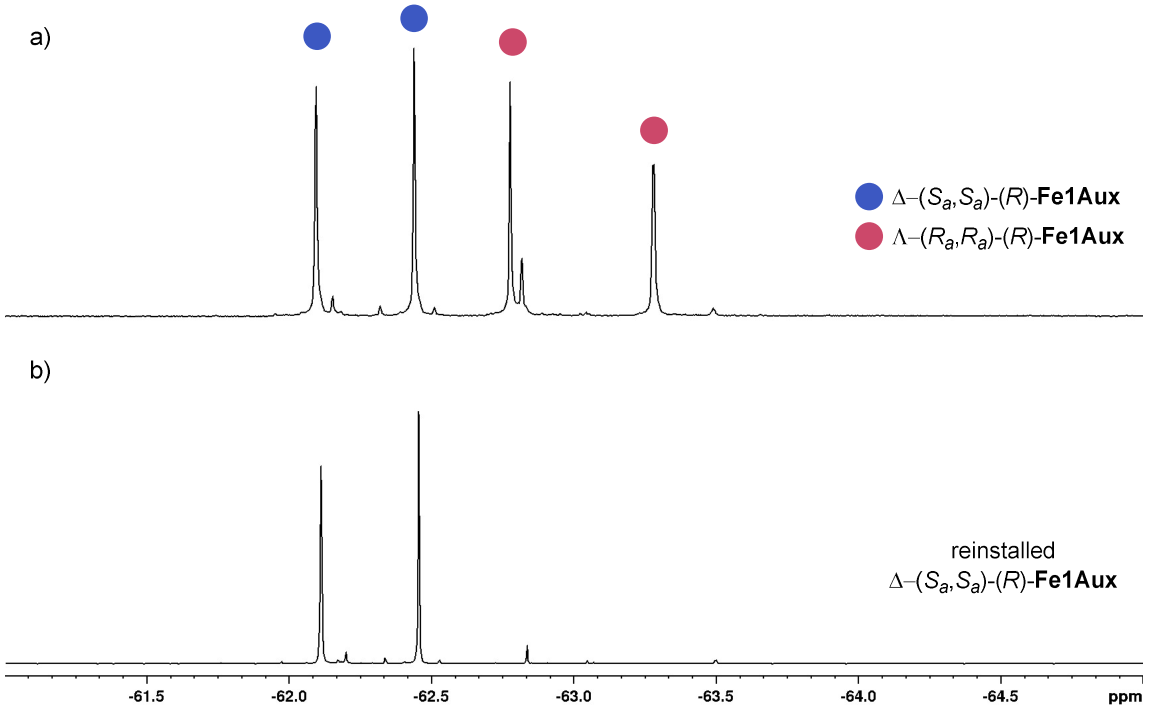

Figure S11: ^{19}F NMR (CD_2Cl_2, 300 K) spectra. a) Crude of the reaction from *rac*-**Fe1-C_2** with (*R*)-Salox. b) Isolated Δ-(S_a,S_a)-(*R*)-**Fe1Aux** after reinstallation of (*R*)-Salox to Δ-(S_a,S_a)-**Fe1-C_2**.

6 Configurational Stability of Ligand 1

Method: Ligand **1** was separated into its enantiomers (R_a)-**1** and (S_a)-**1** by chiral HPLC. After isolation, the sample was reinjected at known time-points and the ee was determined. Decay of ee was used as a measure for the half-life of enantiomerisation and the rotational barrier.

Procedure: Ligand **1** and was dissolved in MeCN and the enantiomers separated by preparative HPLC, the fractions collected and immediately frozen in liquid nitrogen before they were lyophilized overnight. Conditions: Daicel Chiralpak IB column (250 × 10 mm) on an Agilent 1260 Series HPLC System using acetonitrile/water + 0.1 % TFA employing a gradient of 40:60 → 60:40 (*v:v*) in 30 min. as the mobile phase with a flow rate of 1.5 mL/min. The column temperature was 20 °C and UV-absorption was measured at 254 nm.

Upon full removal of the solvents, the enantioenriched sample of **1** was redissolved in HPLC-grade MeCN and the decay of ee was measured via chiral HPLC. Conditions: Daicel Chiralpak IB N-5 column (250 × 4.6 mm) on an Agilent 1260 Series HPLC System using acetonitrile/water + 0.1 % TFA 40:60 → 60:40 (*v:v*) in 30 min. as the mobile phase with a flow rate of 1.5 mL/min. The column temperature was 20 °C and UV-absorption was measured at 254 nm.

Result: The data was analysed by Prism GraphPad (Version 8.0.1). Nonlinear regression of the decay of ee over time gave a half-life $t_{1/2}$ = 277.8 min and a rate constant for enantiomerisation k_{ent} = 2.495*10^{-3} min^{-1} (Figure S12). Applying the EYRING equation gave the energetic barrier to rotation $\Delta G^{\#}$ = 23.4 kcal/mol.[11,12]

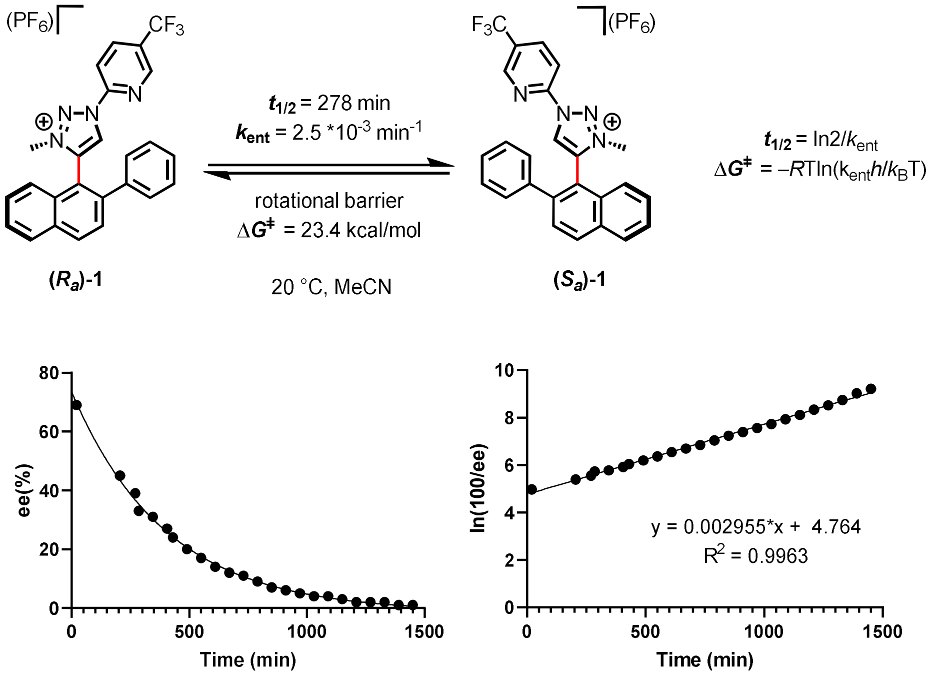

Figure S12: Enantiomerisation of **1** and associated thermodynamic parameters. Rotational barrier ($\Delta G^{\ddagger}$) as Gibbs free energy difference, ideal gas constant R = 8.314472 J/(mol·K), rate constant k_{ent} = 4.1586·10^{-5} s^{-1}, Planck constant h = 6.62606876·10^{-34} J·s, Boltzmann constant k_B = 1.3806503·10^{-23} J/K, absolute Temperature T = 298.15 K.

7 Enantioselective Ring Contraction of Isoxazoles to 2*H*-azirines

General procedure G: Asymmetric catalysis

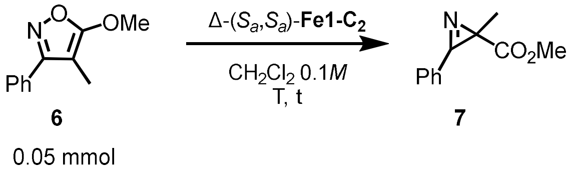

An oven dried SCHLENK tube was charged with substrate **6** (10.0 mg, 0.05 mmol), evacuated and flushed with nitrogen. Subsequently a freshly prepared solution of catalyst Δ-(S_a,S_a)-**Fe1-C$_2$** (0.1–1.5 mol%) in distilled CH_2Cl_2 (0.5 mL, 0.1 *M*) was added under positive N_2-pressure. (If indicated temperature < room temperature, the addition was carried out while the reaction vessel was frozen in liquid nitrogen). The mixture was stirred at the indicated temperature and time. For reactions involving temperatures below room temperature, the reaction was quenched by addition of Silica (50 mg/mL solvent) and subsequent stirring for 15 minutes before 2,2'-bipyridine (4.1 mg, 0.5 equiv.) was added. The reaction was then allowed to warm up to room temperature. Afterwards, the reaction was diluted with EtOAc and filtered over a short plug of silica gel to remove the catalyst. The crude product was further purified by column chromatography on silica gel (*n*-pentane/EtOAc) to yield pure 2*H*-azirine **7**.

Characterisation

Following general procedure G, 2*H*-azirine **7** was obtained as a colourless oil after column chromatography on silica gel (*n*-pentane/EtOAc 10:1). Enantiomeric excess was evaluated by HPLC analysis on a chiral stationary phase of the isolated product. HPLC conditions: Daicel Chiralcel® OJ-H column, 250 x 4.6 mm, absorbance at 254 nm, *n*-hexane/*i*PrOH 80:20, isocratic flow, flow rate 1.0 mL/min, 25 °C, t_r (minor) = 8.76 min, t_r (major) = 11.68 min.

TLC (*n*-pentane/EtOAc 5:1)**:** R_f = 0.35 **^{1}H NMR** (300 MHz, $CDCl_3$): δ (ppm) = 7.84 (d, *J* = 7.3 Hz, 2H), 7.66–7.54 (m, 3H), 3.68 (s, 3H), 1.63 (s, 3H). **^{13}C NMR** (75 MHz, $CDCl_3$): δ (ppm) = 173.67, 163.72, 133.77, 130.26 (2C), 129.49 (2C), 122.69, 52.59, 35.57, 17.88.

Analytical data of **7** are in agreement with published data.[2] The absolute configuration of the product was determined by comparison of the HPLC traces with the literature.[2]

8 Characterisation of Fe(II)-Complexes

rac-**Fe1-C$_2$**-PF$_6$

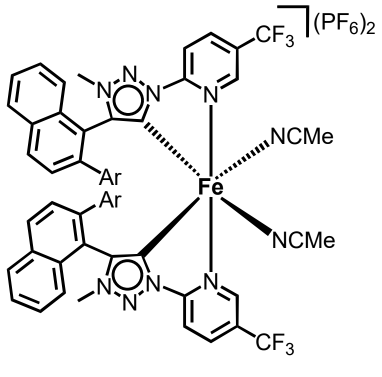

rac-**Fe1-C$_2$**
Ar = Ph

Following general procedure D and Transmetalation Method B, *rac*-**Fe1-C$_2$**-PF$_6$ (66.8 mg, 0.058 mmol, 28%) was obtained as a red solid from the corresponding ligand **1** (218 mg, 0.42 mmol). Solvent volume of reaction: 17 mL MeCN.

Notes: The indicated yield refers to pure isolated material after a first run of column chromatography. Also obtained was *rac*-**Fe1-C$_1$**-PF$_6$ and mixed fractions, alongside decomposed material in the form of free ligand.

TLC (CH_2Cl_2/CD_3CN 6:1)**:** R_f = 0.35. **MP**: 180 °C (decomp.). **^{1}H NMR** (500 MHz, CD_3CN): δ (ppm) = 8.35 (s, 2H), 8.01 (d, *J* = 8.6 Hz, 2H), 7.95 (d, *J* = 8.16 Hz, 2H), 7.67 (dd, *J* = 8.6, 2.1 Hz, 2H), 7.65–7.60 (m, 4H), 7.44–7.36 (m, 10H), 7.27 (ddd, *J* = 8.3, 6.8, 1.3 Hz, 2H), 7.15 (d, *J* = 8.6 Hz, 2H), 6.73 (d, *J* = 8.5 Hz, 2H), 3.59 (s, 6H). **^{19}F NMR** (282 MHz, CD_3CN): δ (ppm) = –62.35 (s, CF_3), –72.94 (d, $^1J_{P,F}$ = 706.48 Hz, PF_6^-). **^{13}C NMR** (125 MHz, CD_3CN): δ (ppm) = 186.24 (2C), 156.48 (2C), 150.93 (q, $^3J_{C,F}$ = 4.4 Hz), 148.93 (2C), 142.19 (2C), 140.13 (2C), 137.06 (q, $^3J_{C,F}$ = 3.4 Hz), 133.41 (2C), 132.72 (2C), 132.55 (2C), 130.06 (4C), 129.81 (4C), 129.62 (2C), 129.15 (2C), 129.01 (2C), 128.88 (2C), 128.10 (2C), 126.48 (q, $^2J_{C,F}$ = 34.4 Hz), 124.33 (2C), 122.79 (q, $^1J_{C,F}$ = 272.4 Hz, Ar-*C*F_3), 120.46 (2C), 113.00 (2C), 38.52 (2C). **IR** (neat): $\tilde{\nu}$ (cm^{-1}) = 3060 (w), 2916 (w), 1619 (w), 1593 (w), 1495 (w), 1446 (w), 1425 (w), 1322 (m), 1300 (w), 1248 (w), 1173 (w), 1138 (w), 1052 (m), 1031 (w), 991 (w), 921 (w), 867 (w), 829 (w), 766 (m), 755 (w), 701 (m), 652 (w), 630 (w), 579 (w), 547 (w), 519 (w), 492 (w), 441 (w). **HRMS** ESI (+); *m/z* calcd. for $C_{50}H_{34}F_6FeN_8$ [M–2 CH_3CN]$^{2+}$: 458.1075, found: 458.1078.

rac-**Fe1-C$_1$**-BF_4

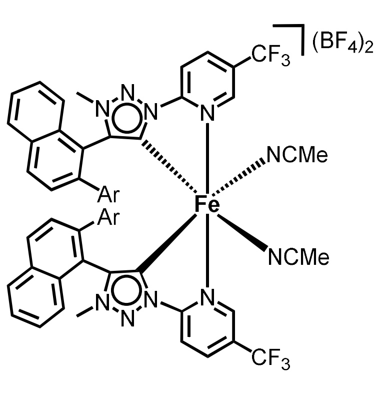
rac-**Fe1-C$_1$**
Ar = Ph

Following general procedure D and Transmetalation Method B, *rac*-**Fe1-C$_1$**-BF_4 (65.1 mg, 0.56 mmol, 27%) was obtained as a red solid from the corresponding ligand **1** (218 mg, 0.42 mmol). Solvent volume of reaction: 17 mL MeCN.

Notes: The indicated yield refers to pure isolated material after a first run of column chromatography. Also obtained was *rac*-**Fe1-C$_2$**-BF_4 and mixed fractions, alongside decomposed material in the form of free ligand.

TLC (CH_2Cl_2/CD_3CN 4:1)**:** R_f = 0.32. **MP**: 170 °C (decomp.). **^{1}H NMR** (500 MHz, CD_3CN): δ (ppm) = 8.28 (d, *J* = 8.7 Hz, 1H), 8.20 (s, 1H), 8.10 (d, *J* = 8.7 Hz, 1H), 8.07 (d, *J* = 8.0 Hz, 1H), 8.00 (d, *J* = 8.7 Hz, 1H), 7.93 (d, *J* = 8.8 Hz, 1H), 7.87 (d, *J* = 8.2 Hz, 1H), 7.72-7.67 (m, 2H), 7.63-7.60 (m, 3H), 7.55 (d, *J* = 8.6 Hz, 1H), 7.39-7.34 (m, 4H), 7.35-7.29 (m, 5H), 7.19 (d, *J* = 8.8 Hz, 1H), 7.15 (t, *J* = 7.7 Hz, 1H), 7.09-7.06 (m, 3H), 5.34 (d, *J* = 8.6 Hz, 1H), 4.20 (s, 3H), 3.32 (s, 3H).**^{19}F NMR** (282 MHz, CD_3CN): δ (ppm) = –62.37 (s, CF_3), –63.28 (s, CF_3), –151.79 ($^{10}BF_4^-$), –151.85 ($^{11}BF_4^-$). **^{13}C NMR** (125 MHz, CD_3CN): δ (ppm) = 187.37, 183.93, 156.92, 156.39, 150.99 (q, ^3J = 4.4 Hz), 150.76 (q, $^3J_{C,F}$ = 5.0 Hz), 149.68, 148.28, 141.94, 141.90, 140.18, 140.09, 137.97 (q, $^3J_{C,F}$ = 3.1 Hz), 137.07 (q, $^3J_{C,F}$ = 3.4 Hz), 133.64, 133.40, 133.20, 132.56, 132.41, 132.32, 132.28, 131.81, 130.31, 130.21 (2C), 130.08 (2C), 129.84 (2C), 129.35 (2C), 129.27, 129.07, 129.04, 128.93, 128.83, 128.24, 128.12, 128.03, 126.41 (q, $^2J_{C,F}$ = 34.3 Hz), 126.28 (q, $^2J_{C,F}$ = 34.6 Hz), 125.01, 122.69 (q, $^1J_{C,F}$ = 272.5 Hz), 122.49 (q, $^1J_{C,F}$ = 272.5 Hz), 120.56, 120.42, 114.69, 112.60, 38.94, 37.91. **IR** (neat): $\tilde{\nu}$ (cm $^{-1}$) = 3083 (w), 1619 (w), 1592 (w), 1495 (w), 1446 (w), 1425 (w), 1405 (w), 1323 (s), 1301 (w), 1248 (w), 1174 (w), 1138 (w), 1053 (s), 1031 (w), 992 (w), 921 (w), 830 (m), 768 (m), 756 (w), 702 (m), 652 (w), 629 (w), 581 (w), 546 (w), 520 (w), 493 (w), 443 (w). **HRMS** ESI (+); *m/z* calcd. for $C_{50}H_{34}F_6FeN_8$ [M–2 CH_3CN]$^{2+}$: 458.1075, found: 458.1068.

rac-**Fe2-C₂**-BF_4

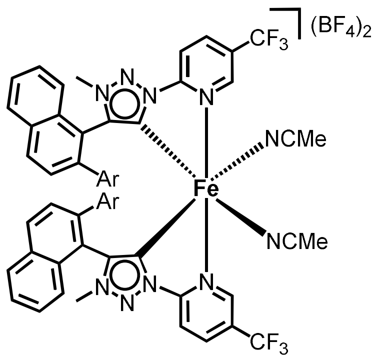

rac-**Fe2-C₂**
Ar = 3,5-(*t*Bu)₂-Ph

Following general procedure D and Transmetalation Method B, *rac*-**Fe2-C₂**-BF_4 (68.1 mg, 0.049 mmol, 56%) was obtained as a red solid from the corresponding ligand **2** (112 mg, 0.18 mmol). Solvent volume of reaction: 7.5 mL MeCN.

Notes: The indicated yield refers to pure isolated material after a first run of column chromatography. Also obtained was *rac*-**Fe2-C₁**-BF_4 and mixed fractions, alongside decomposed material in the form of free ligand.

TLC (CH_2Cl_2/CD_3CN 4:1)**:** R_f = 0.34. **MP**: 177 °C (decomp.). **^{1}H NMR** (500 MHz, CD_3CN): δ (ppm) = 8.08 (s, 2H), 8.05 (d, *J* = 8.3 Hz, 2H), 8.01 (d, *J* = 8.8 Hz, 2H), 7.77 (t, *J* = 7.6 Hz, 2H), 7.72 (d, *J* = 8.7 Hz, 2H), 7.68–7.61 (m, 4H), 7.42 (s, 2H), 6.98 (s, 6H), 6.56 (d, *J* = 8.4 Hz, 2H), 4.07 (s, 6H), 1.20 (s, 36H). **^{19}F NMR** (282 MHz, CD_3CN): δ (ppm) = –61.75 (s, CF_3), –151.77 ($^{10}BF_4^-$), –151.82 ($^{11}BF_4^-$). **^{13}C NMR** (125 MHz, CD_3CN): δ (ppm) = 185.26 (2C), 155.93 (2C), 152.44 (4C), 151.43 (q, $^3J_{C,F}$ = 4.6 Hz, 2C), 149.31 (2C), 142.11 (2C), 139.42 (2C), 137.74 (q, $^3J_{C,F}$ = 3.2 Hz, 2C), 133.35 (2C), 132.71 (2C), 130.24 (2C), 130.17 (2C), 129.90 (2C), 129.69 (2C), 128.24 (2C), 127.07 (q, $^2J_{C,F}$ = 34.4 Hz, 2C), 124.28 (4C), 123.95 (2C), 123.29 (2C), 123.08 (q, $^1J_{C,F}$ = 272.7 Hz, 2C), 119.22 (2C), 112.21 (2C), 38.81 (2C), 35.70 (4C), 31.50 (12C). **IR** (neat): $\tilde{\nu}$ (cm $^{-1}$) = 3063 (w), 2956 (w), 2870 (w), 1616 (w), 1592 (w), 1492 (w), 1426 (w), 1397 (w), 1365 (w), 1324 (s), 1299 (w), 1247 (w), 1215 (w), 1177 (w), 1126 (w), 1076 (w), 1053 (s), 993 (w), 923 (w), 865 (w), 849 (w), 824 (m), 794 (w), 753 (w), 714 (w), 703 (w), 657 (w), 634 (w), 520 (w), 494 (w), 463 (w), 442 (w), 422 (w). **HRMS** ESI (+); *m/z* calcd. for $C_{66}H_{66}F_6FeN_8$ [M–2 CH_3CN]$^{2+}$: 570.2327, found: 570.2332.

rac-**Fe2-C$_1$**-BF_4

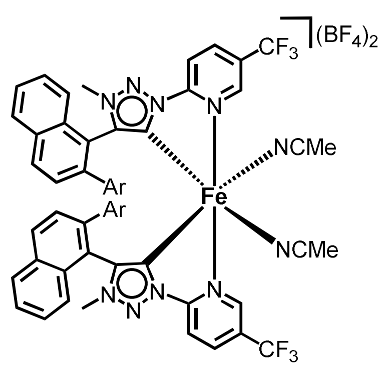

rac-**Fe2-C$_1$**
Ar = 3,5-(*t*Bu)$_2$-Ph

Following general procedure D and Transmetalation Method A, *rac*-**Fe2-C$_1$**-BF_4 (10.0 mg, 0.014 mmol, 12%) was obtained as a red solid from the corresponding ligand **2** (75.0 mg, 0.11 mmol). Solvent volume of reaction: 5 mL MeCN.

Notes: The indicated yield refers to pure isolated material after a first run of column chromatography. Also obtained was *rac*-**Fe2-C$_2$**-BF_4 and mixed fractions, alongside decomposed material in the form of free ligand.

TLC (CH_2Cl_2/CD_3CN 4:1)**:** R_f = 0.33. **MP**: 173 °C (decomp.). **^{1}H NMR** (500 MHz, CD_3CN): δ (ppm) = 8.19 (dd, *J* = 8.4, 1.9 Hz, 1H), 8.07 (d, *J* = 8.5 Hz, 1H), 8.04 (d, *J* = 8.4 Hz, 1H), 8.00 (d, *J* = 8.7 Hz, 1H), 7.99 (d, *J* = 8.5 Hz, 1H) 7.98 (s, 1H), 7.91 (d, *J* = 8.2 Hz, 1H), 7.73 (d, *J* = 8.6 Hz, 1H), 7.69–7.56 (m, 5H), 7.48 (d, *J* = 8.8 Hz, 1H), 7.47 (t, *J* = 1.7 Hz, 1H), 7.41 (d, *J* = 8.7 Hz, 1H), 7.39 (t, *J* = 1.8 Hz, 1H), 7.30 (ddd, *J* = 8.3, 6.9, 1.3 Hz, 1H), 7.07–7.01 (m, 3H), 6.84 (d, *J* = 1.8 Hz, 2H), 6.02 (dd, *J* = 8.3, 1.0 Hz, 1H), 4.20 (s, 3H), 3.08 (s, 3H), 1.35 (s, 18H), 1.11 (s, 18H). **^{19}F NMR** (565 MHz, CD_3CN): δ (ppm) = –62.24 (s, CF_3), –63.17 (s, CF_3), –151.74 ($^{10}BF_4^-$), –151.79 ($^{11}BF_4^-$). **^{13}C NMR** (126 MHz, CD_3CN): δ (ppm) = 187.87, 185.05, 156.77, 156.65, 152.55 (2C), 152.42 (2C), 151.32 (q, $^3J_{C,F}$ = 4.8 Hz), 150.93 (q, $^3J_{C,F}$ = 4.6 Hz), 149.98, 148.12, 143.30, 141.55, 139.52, 138.65, 137.98 (q, $^3J_{C,F}$ = 2.7 Hz), 136.68 (q, $^3J_{C,F}$ = 3.3 Hz), 134.01, 133.31, 133.19, 132.73, 132.65, 132.64, 131.76, 131.25, 130.25, 130.06, 129.72 (2C), 129.41, 128.92, 127.95, 127.90, 126.78 (q, $^2J_{C,F}$ = 34.4 Hz), 126.19 (q, $^2J_{C,F}$ = 34.4 Hz), 125.21, 124.89, 124.59 (2C), 124.35, 123.76 (2C), 122.78 (q, *J* = $^1J_{C,F}$ = 272.7 Hz), 122.62, 122.44 (q, $^1J_{C,F}$ = 272.7 Hz), 120.00, 119.09, 114.05, 114.03, 39.00, 37.95, 35.79, 35.46, 31.75 (6C), 31.37 (6C). **IR** (neat): $\tilde{\nu}$ (cm $^{-1}$) = 3077 (w), 2959 (w), 2870 (w), 1665 (w), 1619 (w), 1592 (w), 1489 (w), 1424 (w), 1396 (w), 1364 (w), 1323 (s), 1299 (w), 1267 (w), 1247 (w), 1173 (w), 1137 (w), 1054 (s), 994 (w), 922 (w), 898 (w), 869 (w), 827 (m), 794 (w), 754 (w), 733 (w), 718 (w), 702 (m), 656 (w), 635 (w), 520 (w), 498 (w), 483 (w), 465 (w), 444 (w), 431 (w). **HRMS** ESI (+); *m/z* calcd. for $C_{66}H_{66}F_6FeN_8$ [M–2 CH_3CN]$^{2+}$: 570.2327, found: 570.2330.

Δ-(S_a,S_a)-(R)-**Fe1Aux**-BF_4 and Λ-(R_a,R_a)-(S)-**Fe1Aux**-BF_4

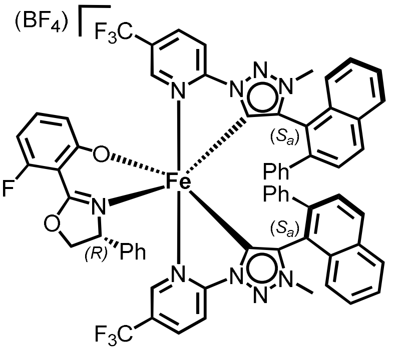

Δ–(S_a,S_a)-(R)-**Fe1Aux**

Following General Procedure E, Δ-(S_a,S_a)-(R)-**Fe1Aux**-BF_4 (19.6 mg, 15.6 µmol, 46 %) was obtained as a green solid from the corresponding Complex *rac*-**Fe1-C₂**-BF_4 (40 mg, 34 µmol).

Λ-(R_a,R_a)-(S)-**Fe1Aux**-BF_4 (3.0 mg, 2.4 µmol, 37 %) was obtained in analogous fashion as a green solid from the corresponding complex *rac*-**Fe1-C₂**-BF_4 (8.0 mg, 6.4 µmol).

The enantiomers show the same NMR signals and mirror image behaviour in CD-spectroscopy.

Analytical data of Δ-(S_a,S_a)-(R)-**Fe1Aux**-BF_4:

TLC (CH_2Cl_2/CD_3CN 10:1)**:**R_f = 0.35. **MP**: 204 °C (decomp.). **^{1}H NMR** (500 MHz, $(CD_3)_2CO$): δ (ppm) = 8.48 (s, 1H), 8.16 (s, 1H), 8.05 (d, J = 8.7 Hz, 1H), 8.03–7.95 (m, 3H), 7.87–7.72 (m, 7H), 7.71–7.64 (m, 1H), 7.65 (d, J = 9.0 Hz, 1H), 7.61 (ddd, J = 8.1, 6.9, 1.2 Hz, 1H), 7.58–7.50 (m, 2H), 7.51–7.40 (m, 3H), 7.36 (t, J = 7.4 Hz, 1H), 7.31–7.24 (m, 2H), 7.16 (d, J = 8.6 Hz, 1H), 6.95–6.88 (m, 1H), 6.82 (t, J = 7.3 Hz, 1H), 6.73 (br s, 2H), 6.67 (d, J = 8.4 Hz, 1H), 6.56 (d, J = 8.6 Hz, 1H), 6.48 (d, J = 8.7 Hz, 1H), 6.33 (d, J = 8.5 Hz, 1H), 6.06 (br s, 2H), 5.96 (dd, J = 11.9, 7.9 Hz, 1H), 4.63 (s, 3H), 4.38 (t, J = 9.4 Hz, 1H), 3.76 (dd, J = 9.3, 3.9 Hz, 1H), 3.45 (s, 3H), 3.35 (dd, J = 9.5, 3.9 Hz, 1H). **^{19}F NMR** (282 MHz, $(CD_3)_2CO$): δ (ppm) = –62.30 (s, CF_3), –62.62 (s, CF_3), –108.00 (s, ArF), –151.72 ($^{10}BF_4^-$), –152.77 ($^{11}BF_4^-$). **^{13}C NMR** (126 MHz, $(CD_3)_2CO$): δ (ppm) = 192.71, 192.65, 176.23 (d, $^3J_{C,F}$ = 3.4 Hz, Ar-F), 166.16 (d, $^3J_{C,F}$ = 3.3 Hz, Ar-F), 163.59 (d, $^1J_{C,F}$ = 258.8 Hz, Ar-F), 158.18, 157.49, 150.46 (q, $^3J_{C,F}$ = 4.7 Hz, Ar-CF_3), 149.74, 149.05 (q, $^3J_{C,F}$ = 4.8 Hz, Ar-CF_3), 147.60, 142.72, 142.18, 141.90, 141.49, 140.49, 135.01 (q, $^3J_{C,F}$ = 3.5 Hz, Ar-CF_3), 134.05 (q, $^3J_{C,F}$ = 3.8 Hz, Ar-CF_3), 133.95 (d, $^3J_{C,F}$ = 14.3 Hz, Ar-F), 133.66, 133.49, 133.43, 132.97, 132.38, 132.12, 130.94 (2C), 130.82 (2C), 129.90 (2C), 129.85, 129.75, 129.71 (2C), 129.53, 129.42, 129.23 (2C), 129.11, 128.87, 128.68, 128.56 (2C), 127.86, 127.77, 126.41 (2C), 125.12 (q, $^2J_{C,F}$ = 30.5 Hz, Ar-CF_3), 124.90 (q, $^2J_{C,F}$ = 33.7 Hz, Ar-CF_3), 124.28, 123.85, 123.50 (q, $^1J_{C,F}$ = 275.3 Hz, Ar-CF_3), 123.30 (q, $^1J_{C,F}$ = 270.2 Ar-CF_3 121.74, 121.20, 120.09 (d, $^4J_{C,F}$ = 3.1 Hz, Ar-F), 112.60, 111.21, 101.51 (d, $^2J_{C,F}$ = 7.2 Hz, Ar-F), 100.23 (d, $^2J_{C,F}$ = 22.8 Hz, Ar-F), 76.49, 71.73, 39.25, 37.52. **IR** (neat): $\tilde{\nu}$ (cm $^{-1}$) = 3060 (w), 2959 (w), 2923 (m), 2852 (w), 1724 (w), 1677 (w), 1618 (m), 1582 (w), 1534 (w), 1493 (w), 1448 (m), 1422 (w), 1385 (w), 1320 (s), 1294 (w), 1260 (w), 1231 (w), 1171 (w), 1136 (w), 1099 (w), 1076 (w), 1057 (m), 1041 (w), 985 (w), 947 (w), 924 (w), 865 (w), 827 (w), 794 (w), 768 (w), 701 (m), 651 (w), 630 (w), 580 (w), 532 (w), 493 (w), 410 (w). **HRMS** ESI (+); m/z calcd. for $C_{65}H_{45}F_7FeN_9O_2$ $[M]^+$: 1172.2930, found: 1172.2906.

Δ-(S_a,S_a)-(R)-**Fe1Aux**-BF_4

CD (CH_2Cl_2): Λ, nm ($\Delta\varepsilon$, $M^{-1}cm^{-1}$) 256.5 (−216), 296 (+48), 352 (−20), 393.5 (−9), 411.5 (−11), 461 (−2), 521 (−10).

Λ-(R_a,R_a)-(S)-**Fe1Aux**-BF_4

CD (CH_2Cl_2): Λ, nm ($\Delta\varepsilon$, $M^{-1}cm^{-1}$) 255.5 (+219), 297 (−49), 354.5 (+21), 393.5 (+9), 415.5 (+12), 458 (+3), 521.5 (+11).

Δ-(S_a,S_a)-**Fe1**-BF_4

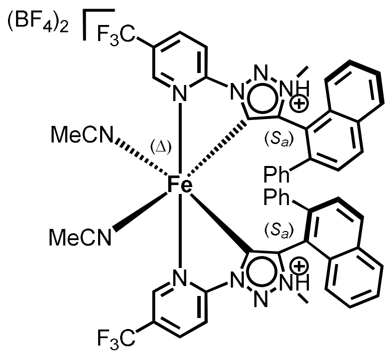

Δ–(S_a,S_a)-**Fe1-C$_2$**

Following general procedure F, Δ-(S_a,S_a)-**Fe1**-BF_4 (12.0 mg, 10.2 µmol, 65 %) was obtained as a red solid from the corresponding Δ-(S_a,S_a)-(R)-**Fe1Aux**-BF_4 (19.6 mg, 15.6 µmol).

NMR-data matches with *rac*-**Fe1-C$_2$.**

CD (CH_3CN, 0.25 mM): Λ, nm ($\Delta\varepsilon$, $M^{-1}cm^{-1}$) 213 (−111), 234 (−20), 242.5 (+2), 257 (−131), 296.6 (+37), 333 (−2), 395 (−8), 442 (−12), 490 (+10).

Λ-(R_a,R_a)-**Fe1**-BF_4

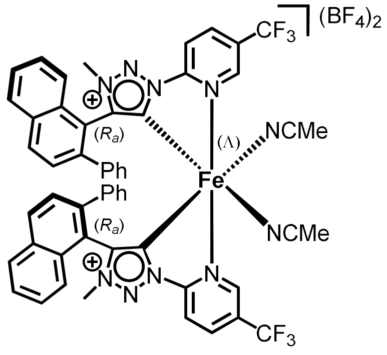

Λ–(R_a,R_a)-**Fe1-C$_2$**

Following general procedure F, Λ-(R_a,R_a)-**Fe1**-BF_4 (0.50 mg, 0.43 µmol, 54 %) was obtained as a red solid from the corresponding Λ-(R_a,R_a)-(S)-**Fe1Aux**-BF_4 (1.0 mg, 0.79 µmol).

NMR-data matches with *rac*-**Fe1-C$_2$.**

CD (CH_3CN, 0.25 mM): Λ, nm ($\Delta\varepsilon$, $M^{-1}cm^{-1}$) 214.5 (+104), 233 (+17), 242.5 (+4), 257 (+130), 295.6 (−38), 333.5 (+2), 395 (+6), 441 (+13), 490 (−6).

9 NMR-Spectra

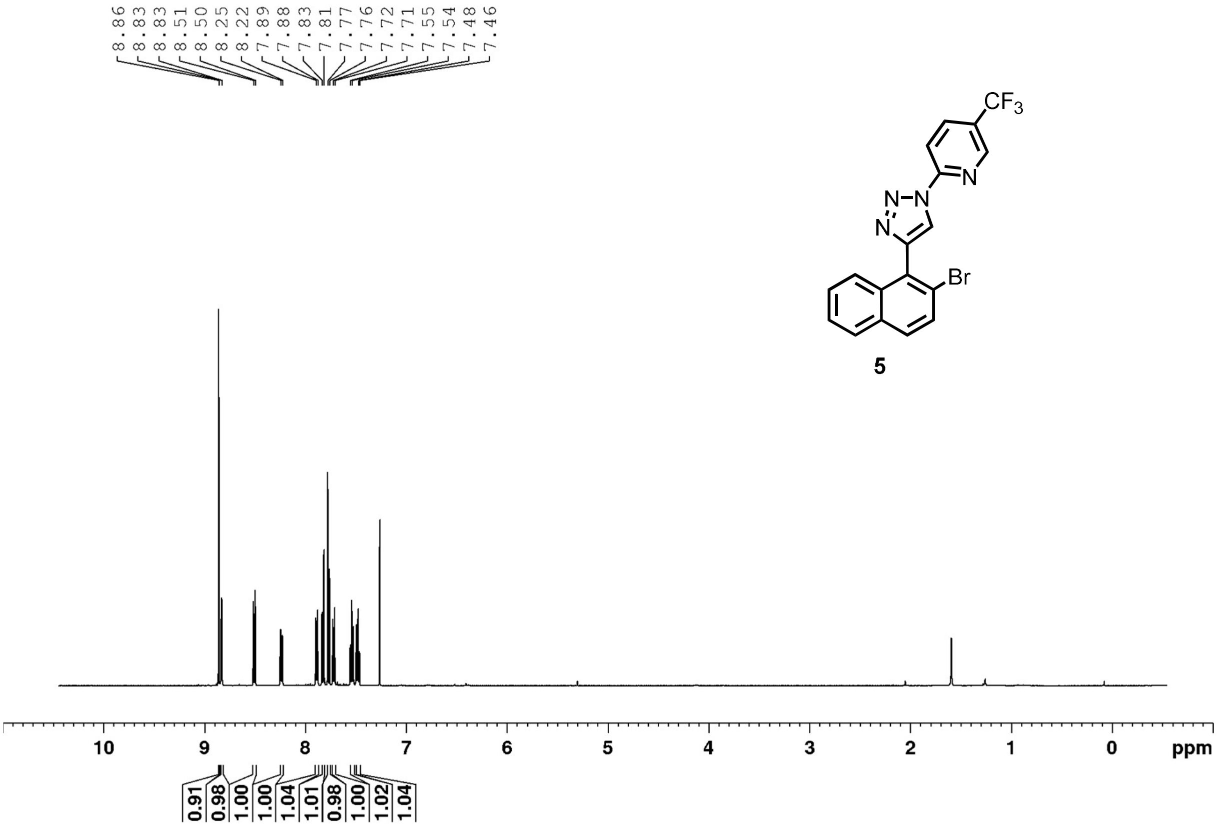

Figure S13 ^{1}H NMR spectrum of **5** (500 MHz, $CDCl_3$, 300 K).

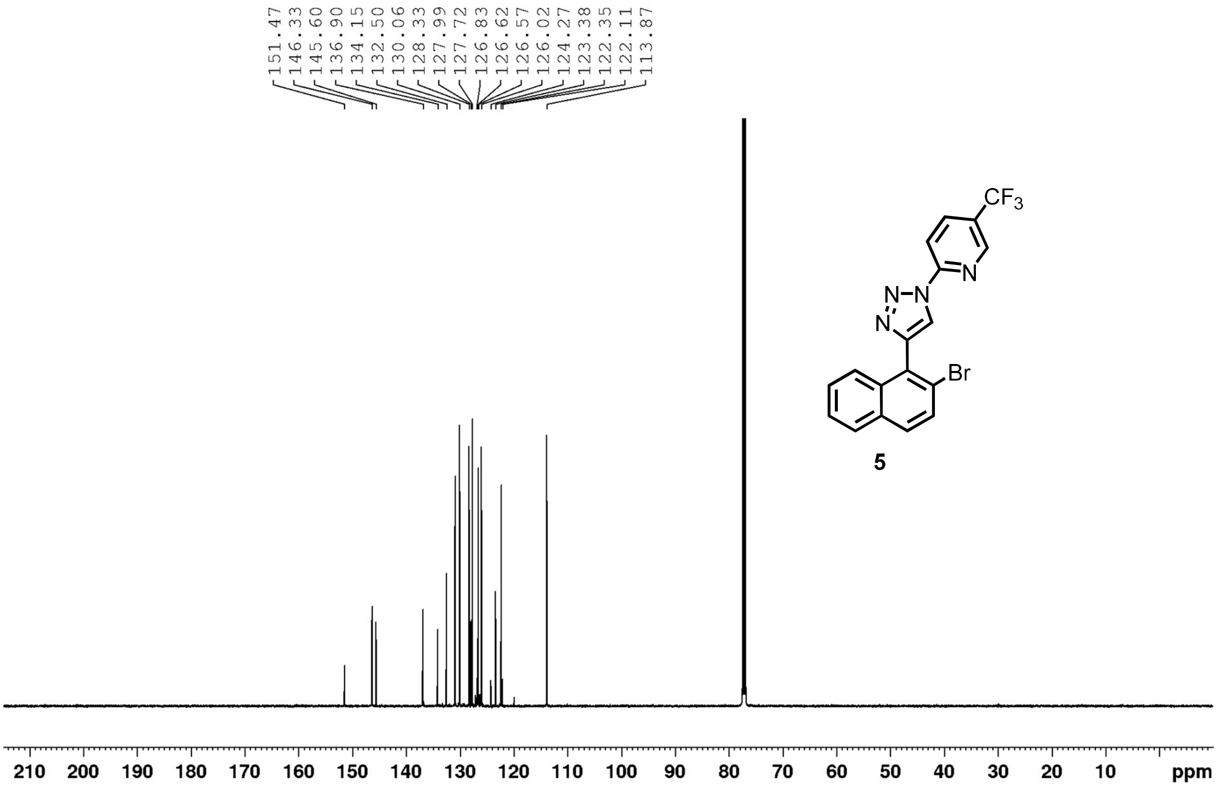

Figure S14: ^{13}C NMR spectrum of **5** (125 MHz, $CDCl_3$, 300 K).

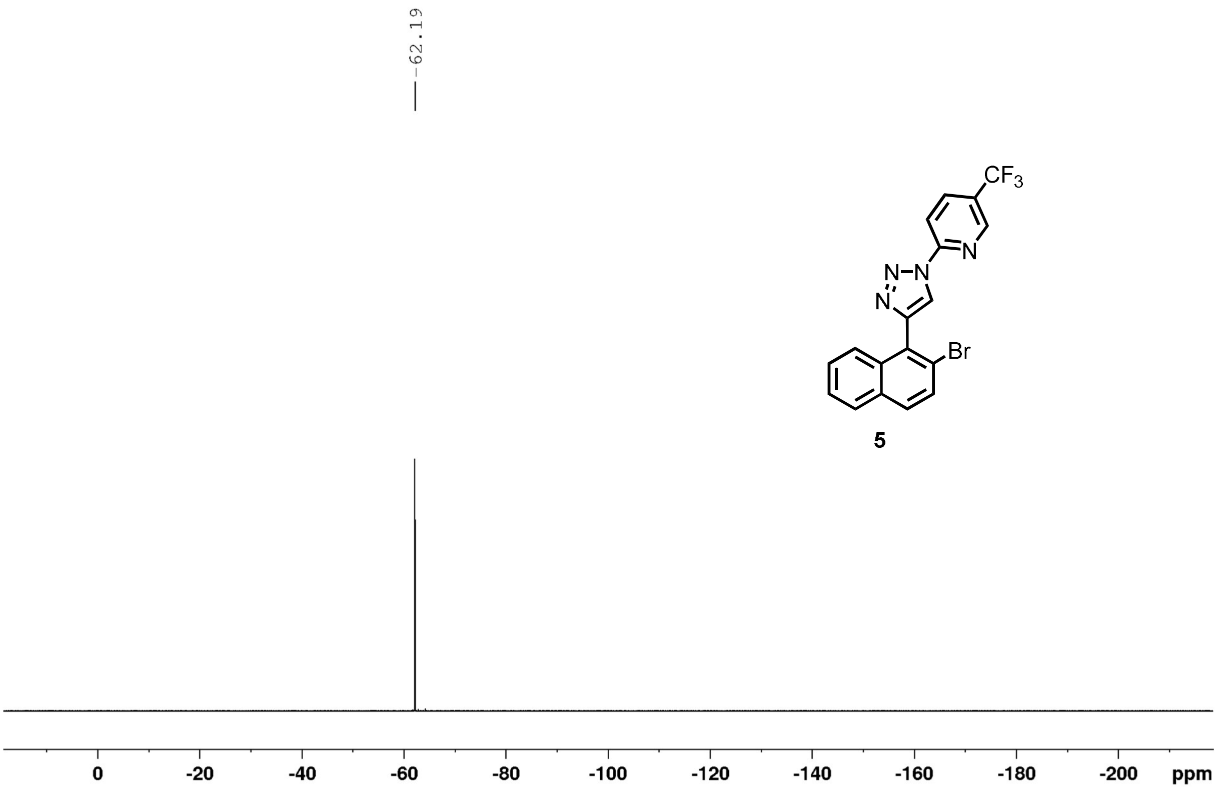

Figure S15: ^{19}F NMR spectrum of **5** (282 MHz, $CDCl_3$, 300 K).

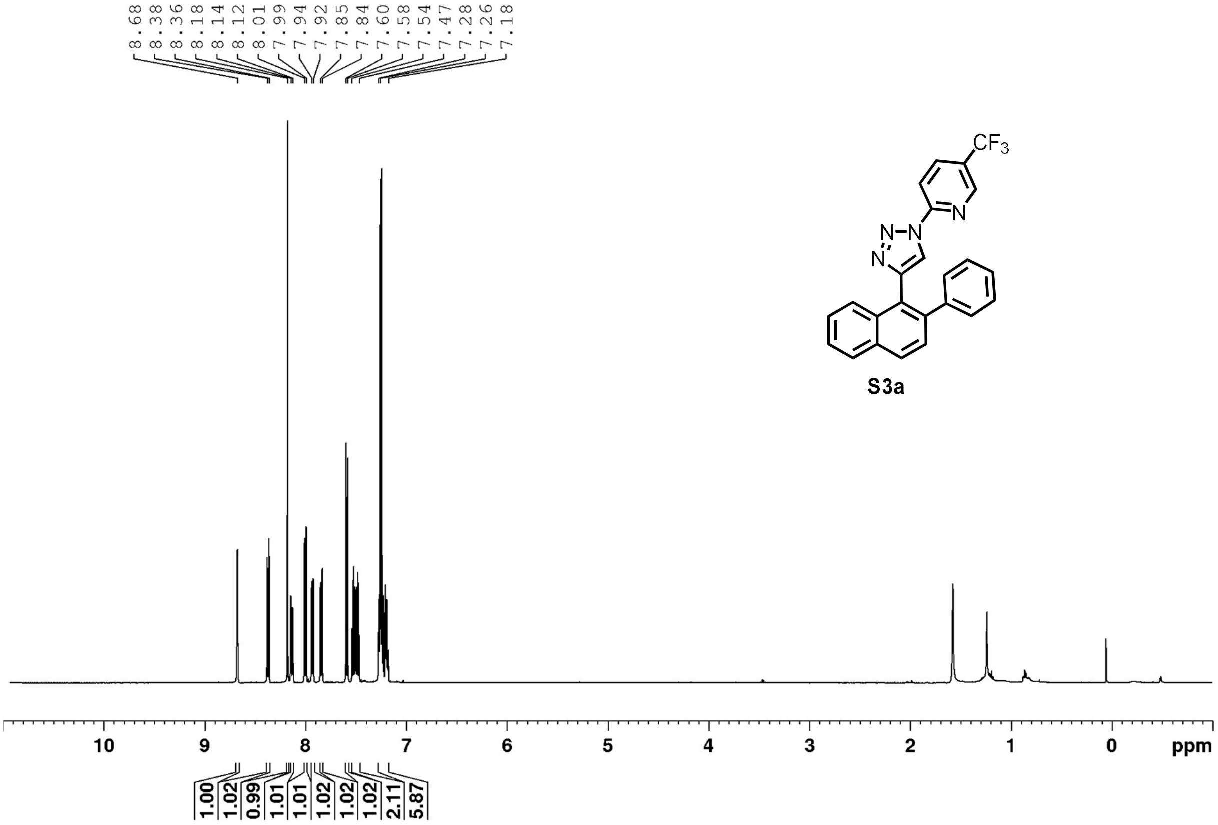

Figure S16: ^{1}H NMR spectrum of **S3a** (500 MHz, $CDCl_3$, 300 K).

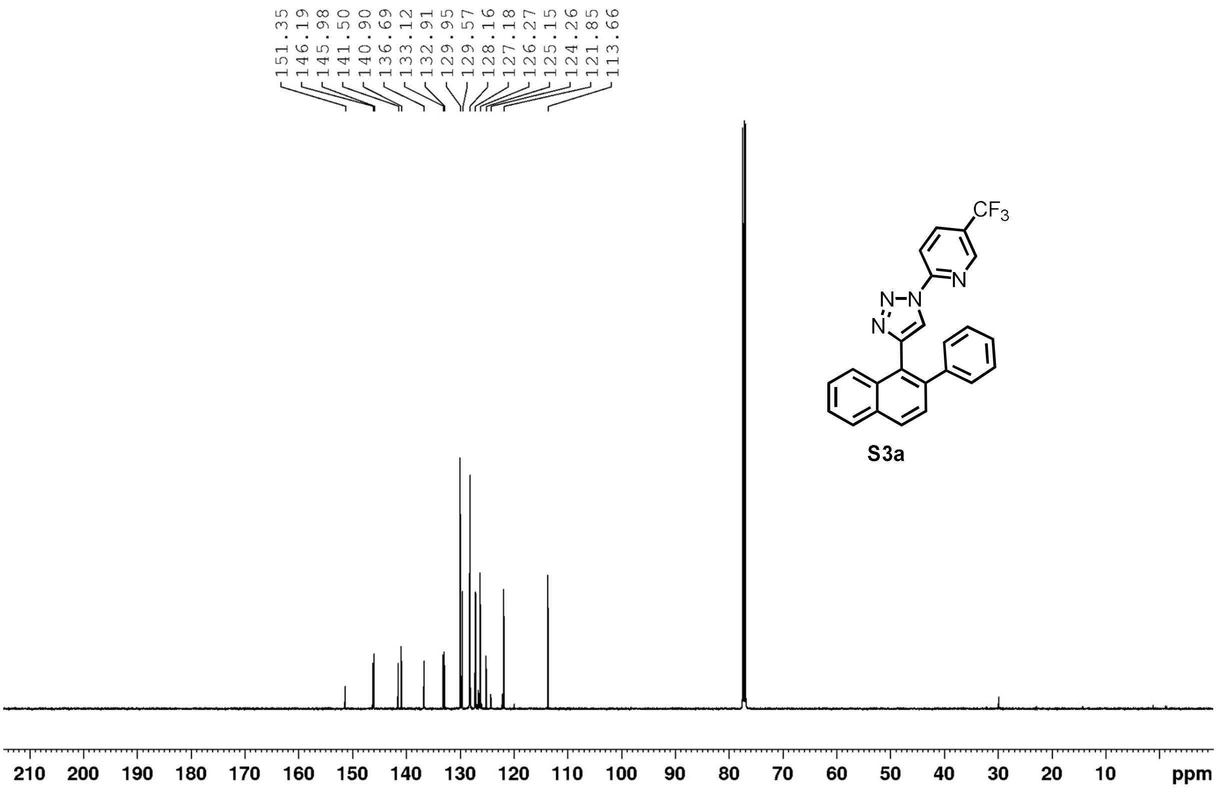

Figure S17: ^{13}C NMR spectrum of **S3a** (125 MHz, $CDCl_3$, 300 K).

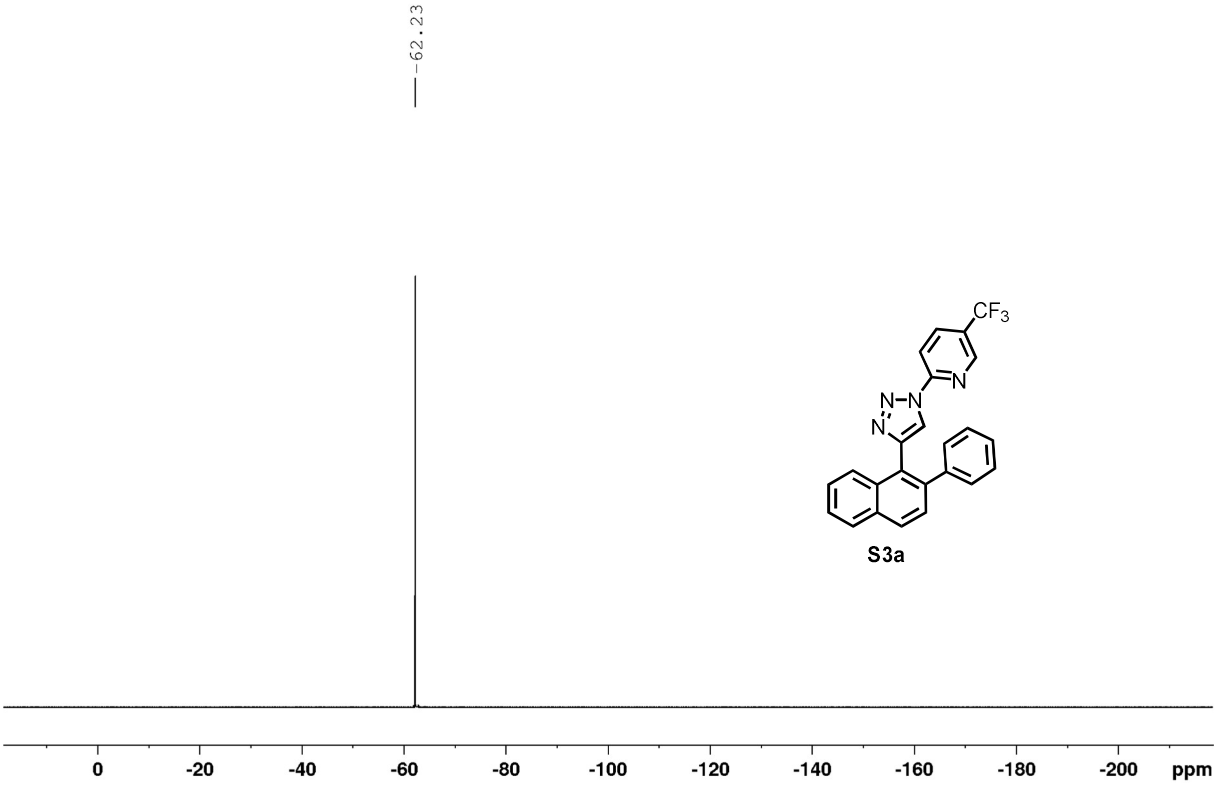

Figure S18: ^{19}F NMR spectrum of **S3a** (282 MHz, $CDCl_3$, 300 K).

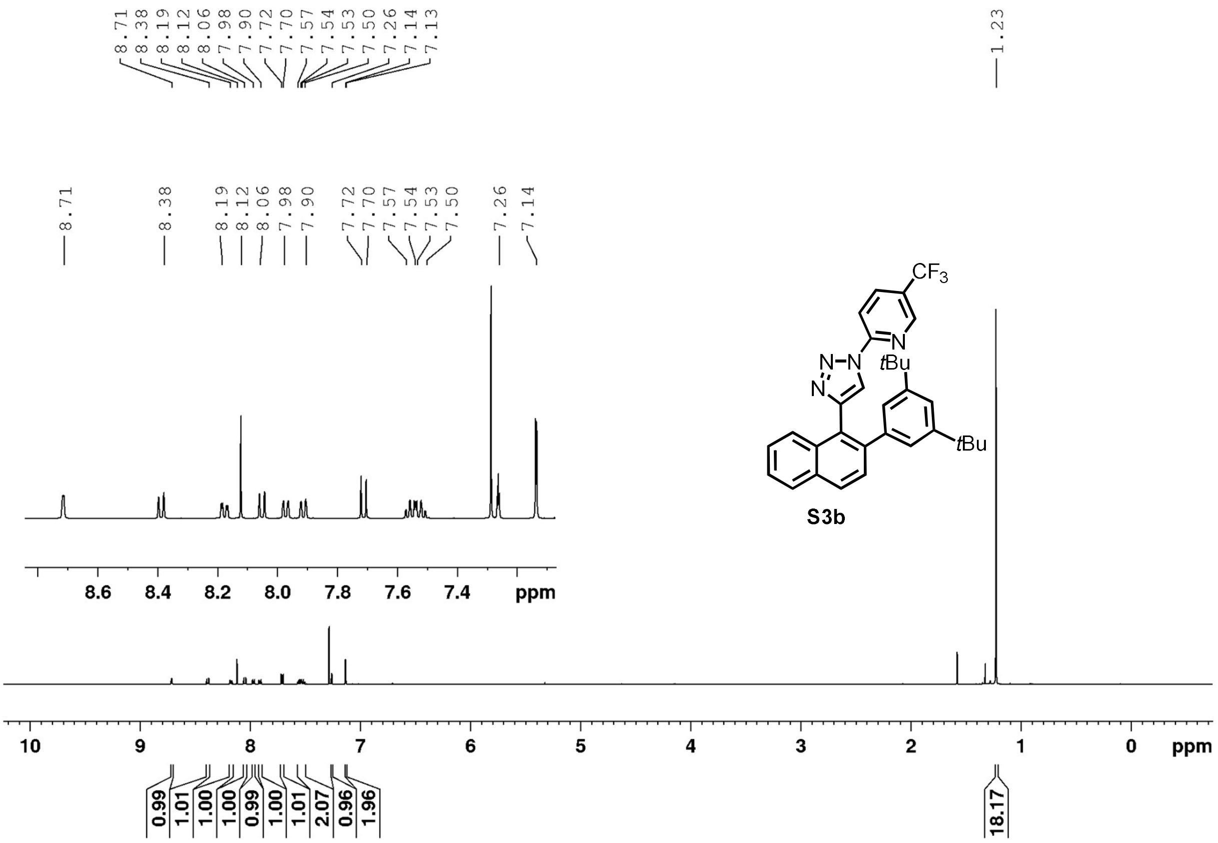

Figure S19: ^{1}H NMR spectrum of **S3a** (500 MHz, $CDCl_3$, 300 K).

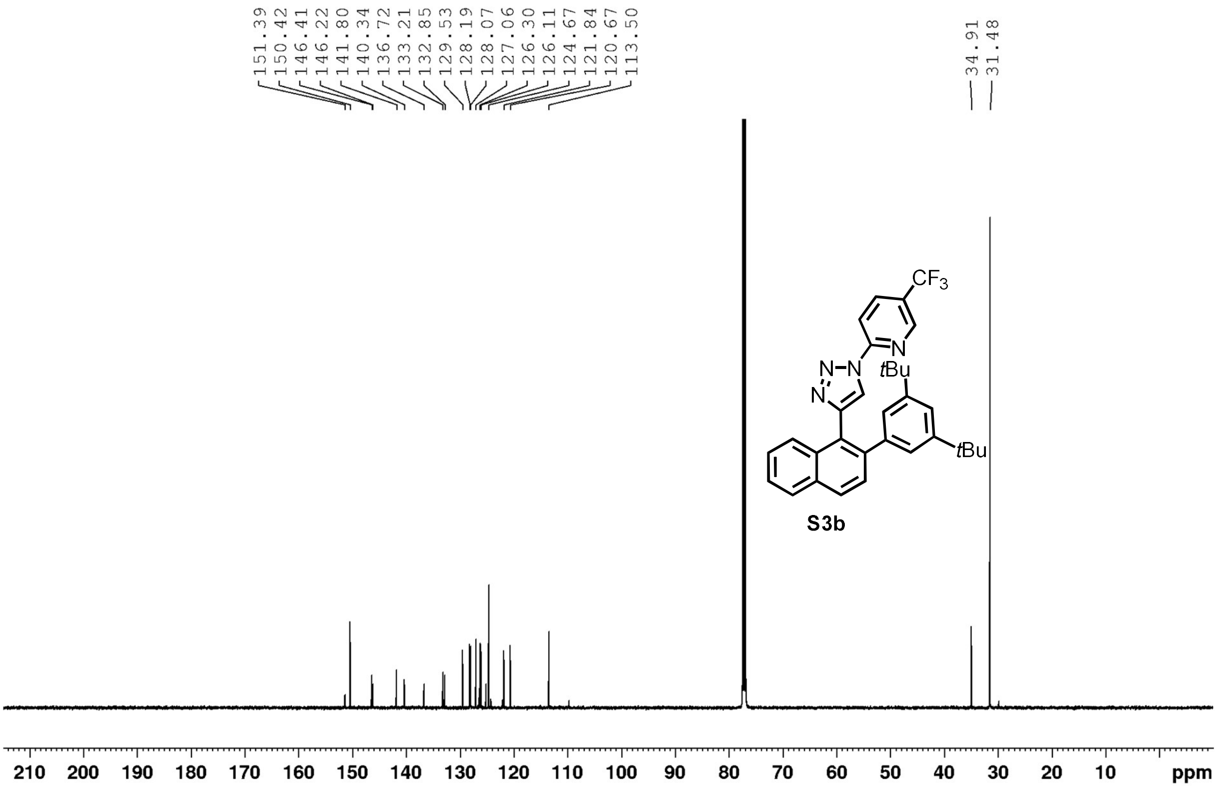

Figure S20: ^{13}C NMR spectrum of **S3b** (125 MHz, $CDCl_3$, 300 K).

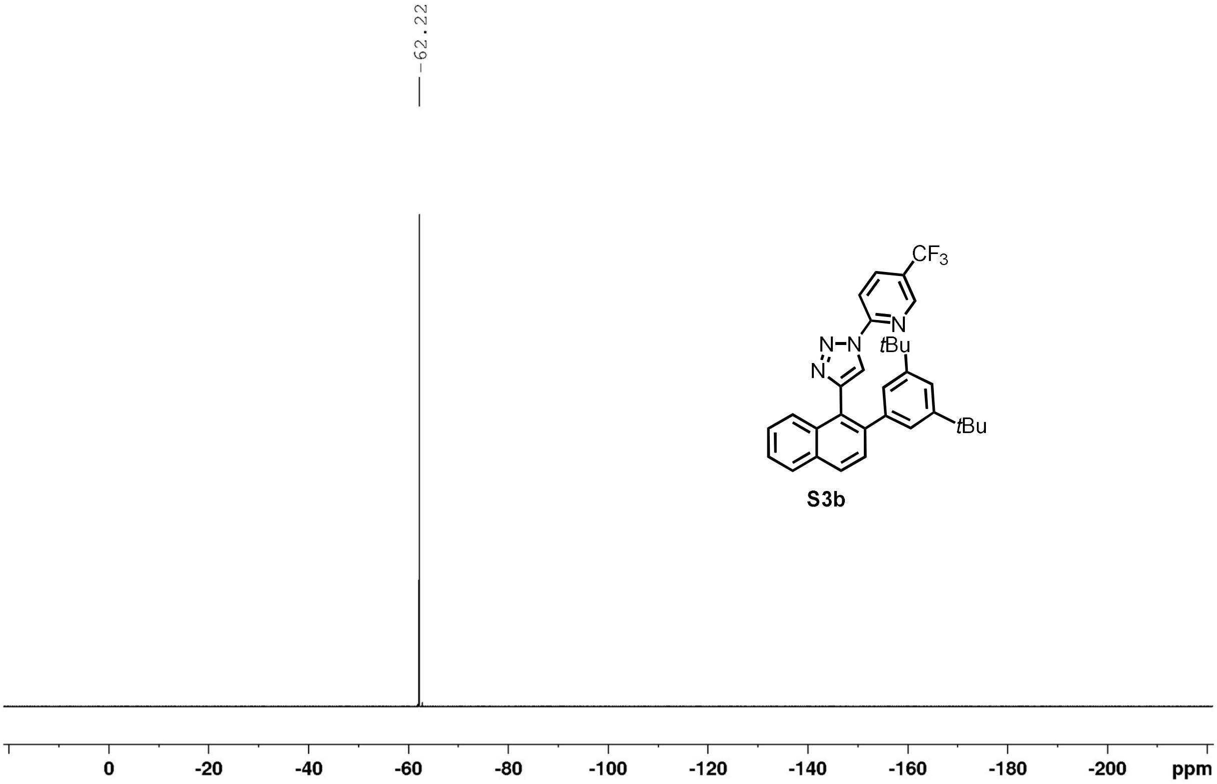

Figure S21: ^{19}F NMR spectrum of **S3b** (282 MHz, $CDCl_3$, 300 K).

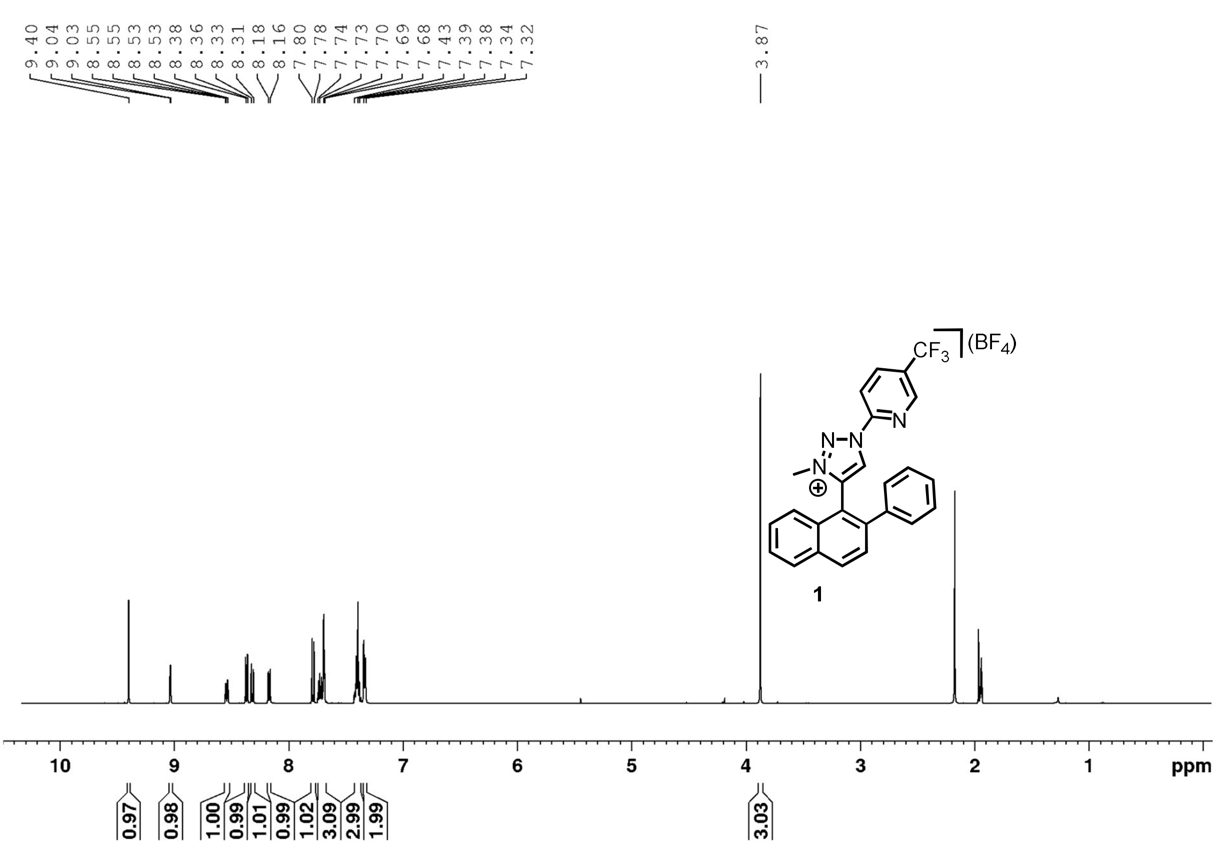

Figure S22: ^{1}H NMR spectrum of **1** (600 MHz, CD_3CN, 300 K).

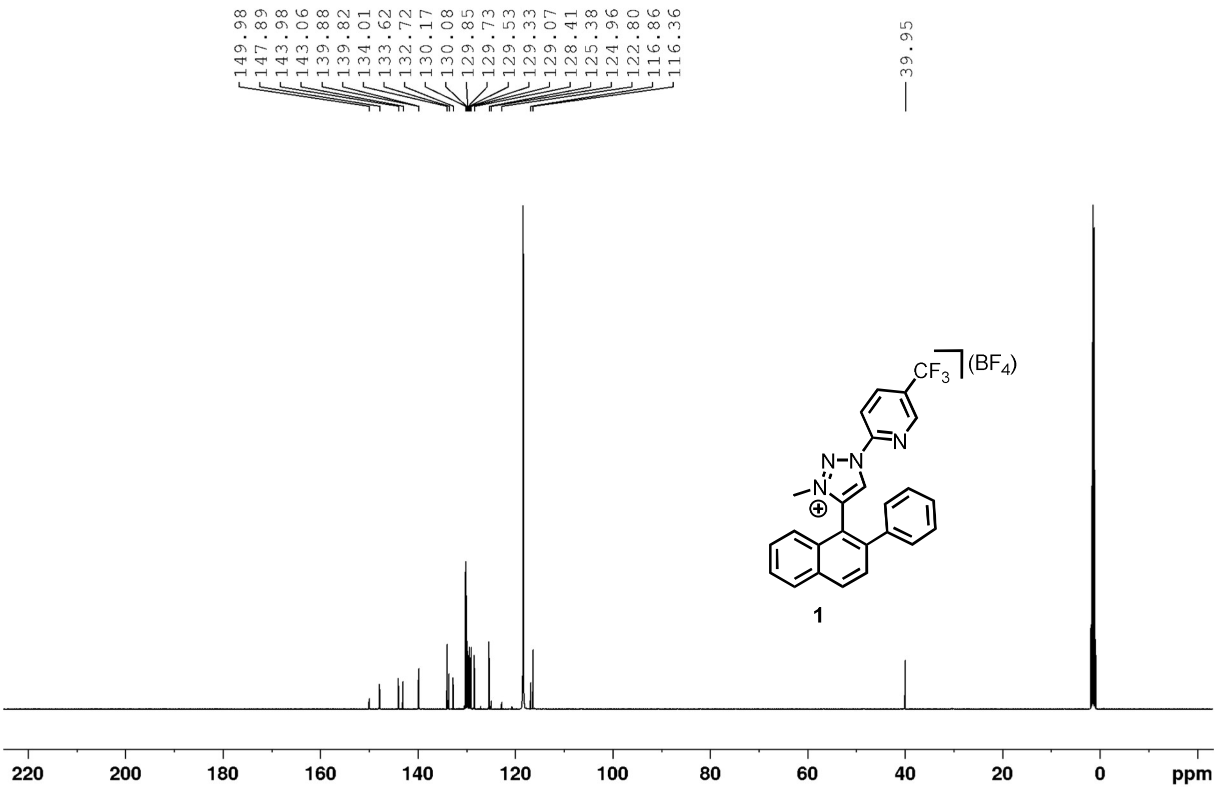

Figure S23: ^{13}C NMR spectrum of **1** (125 MHz, CD_3CN, 300 K).

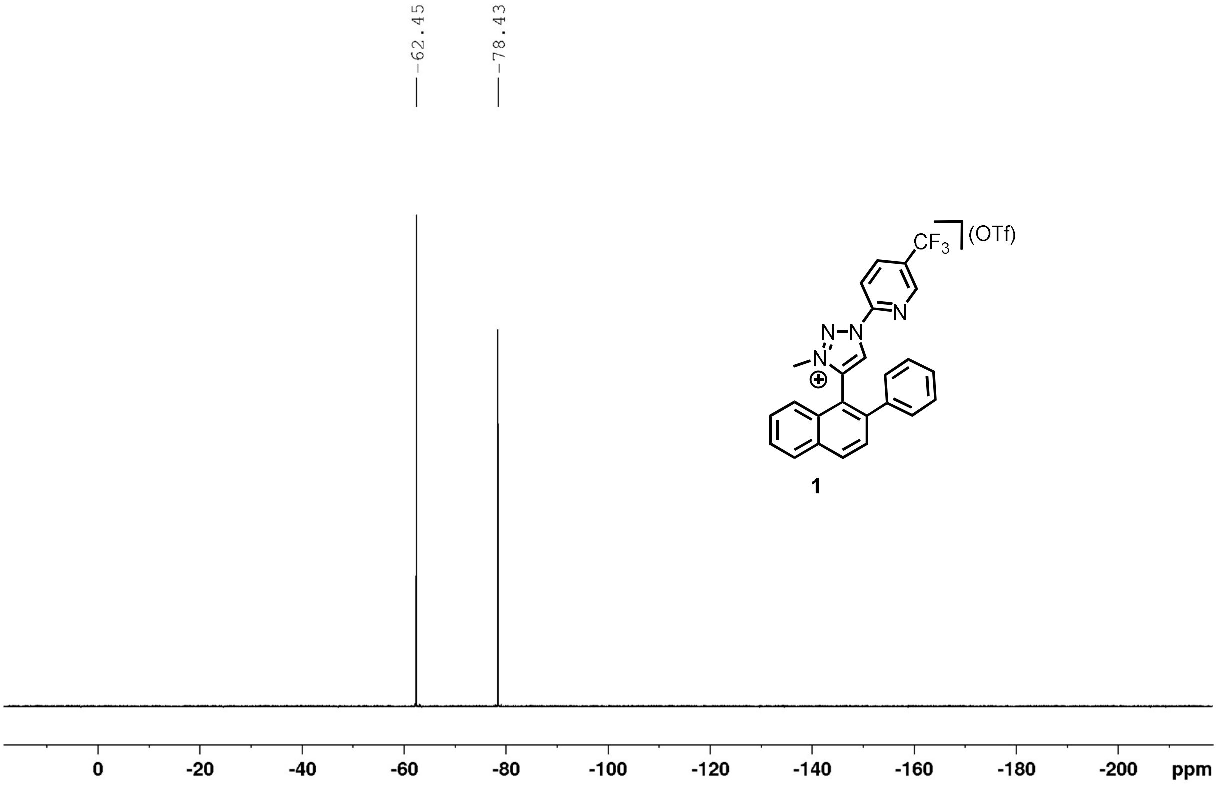

Figure S24: ^{19}F NMR spectrum of **1** (282 MHz, $CDCl_3$, 300 K).

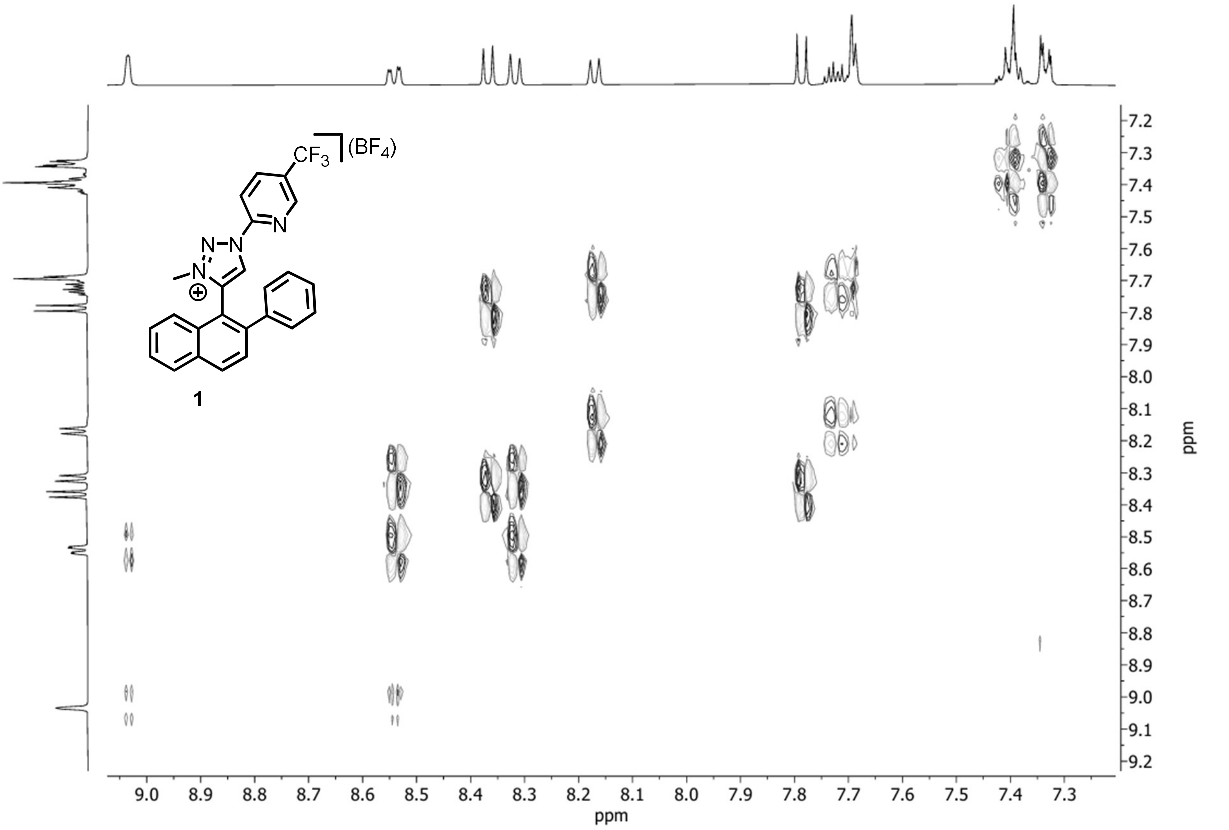

Figure S 25: DQF-COSY NMR spectrum of **1** (500 MHz, CD_3CN, 300 K).

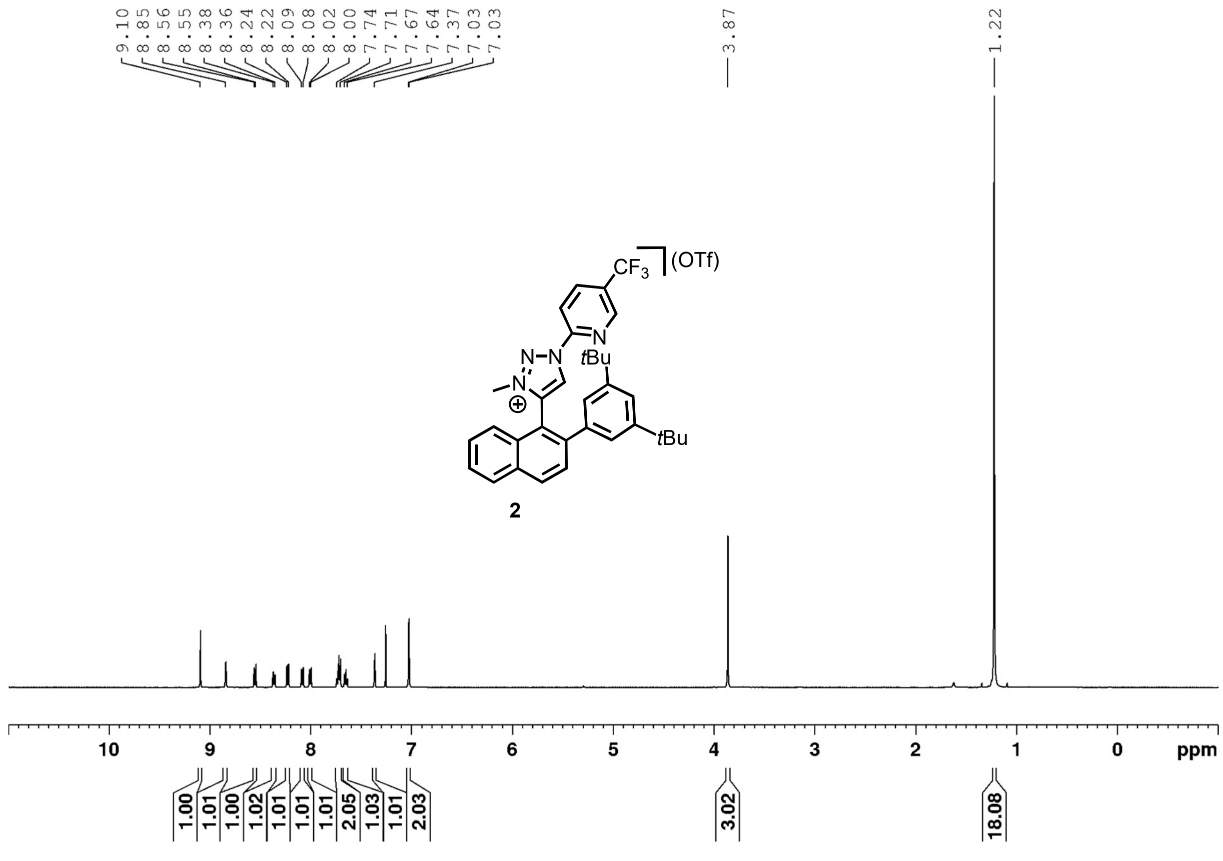

Figure S26: ^1H NMR spectrum of **2** (500 MHz, $CDCl_3$, 300 K).

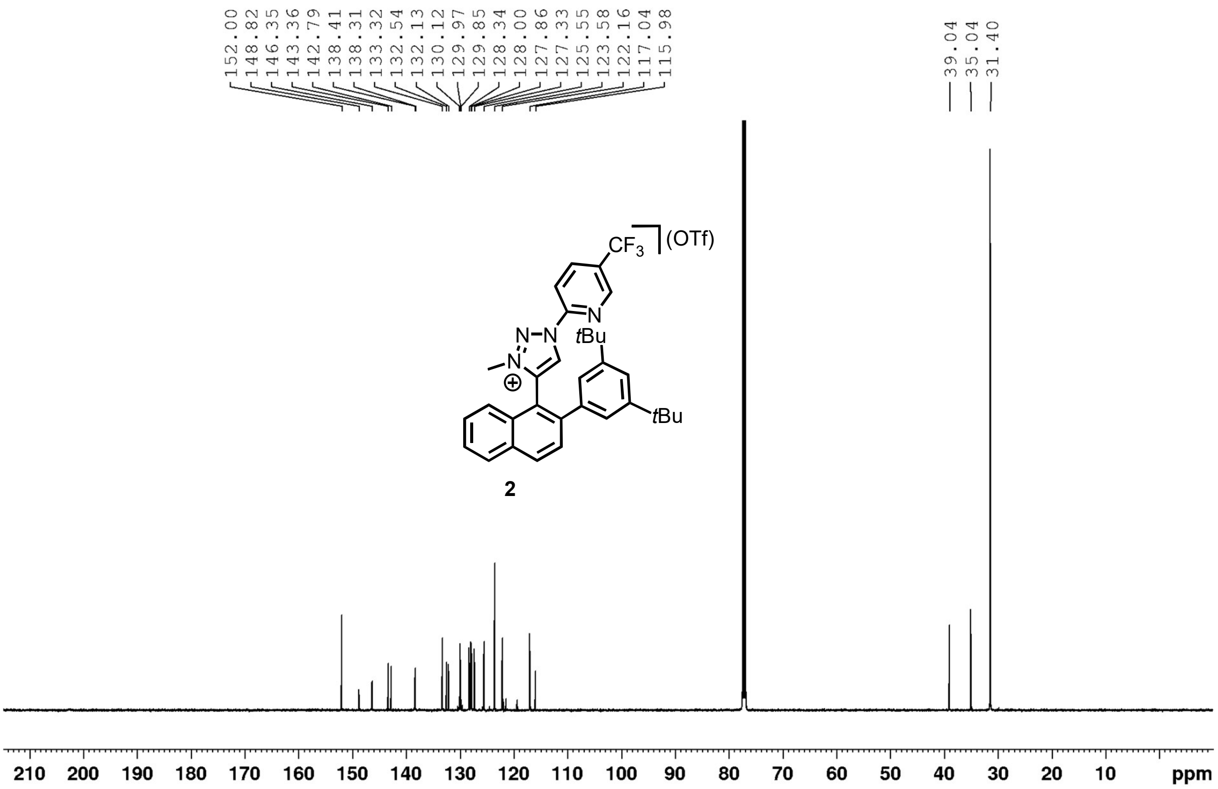

Figure S27: ^{13}C NMR spectrum of **2** (150 MHz, $CDCl_3$, 300 K).

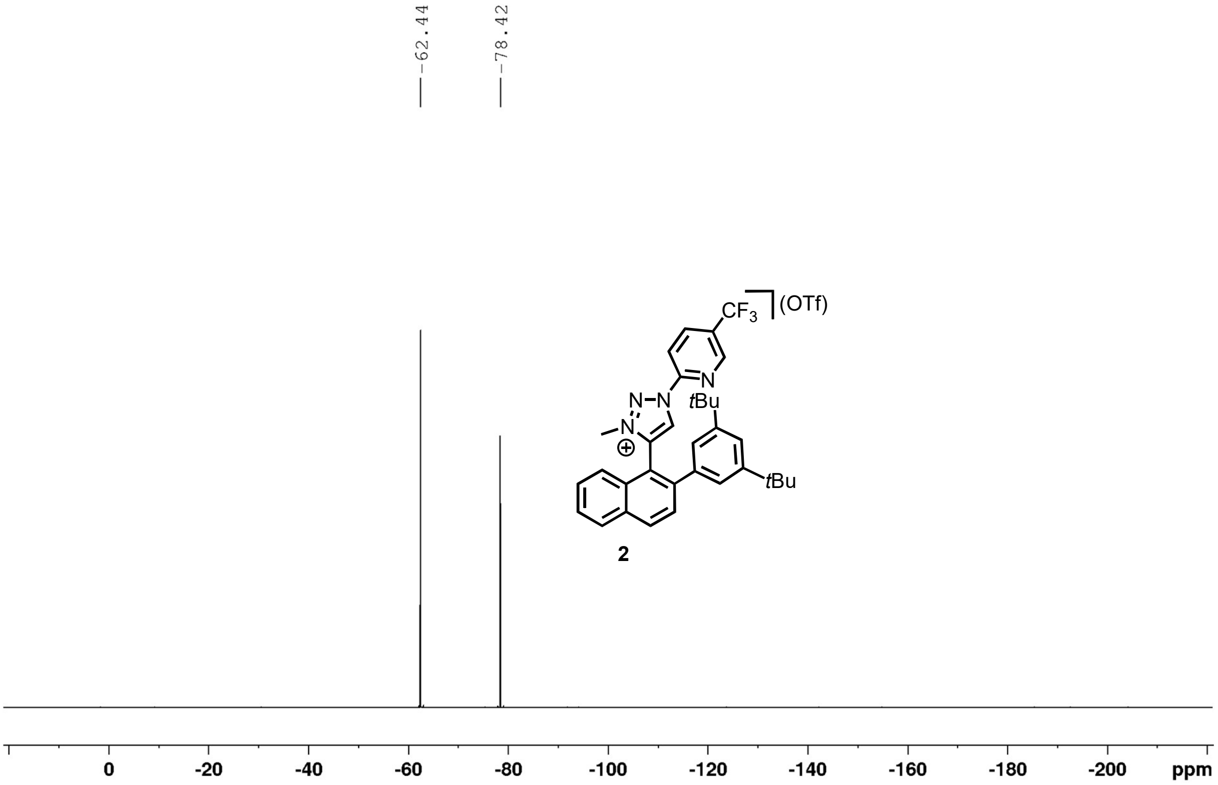

Figure S28: ^{19}F NMR spectrum of **2** (282 MHz, $CDCl_3$, 300 K).

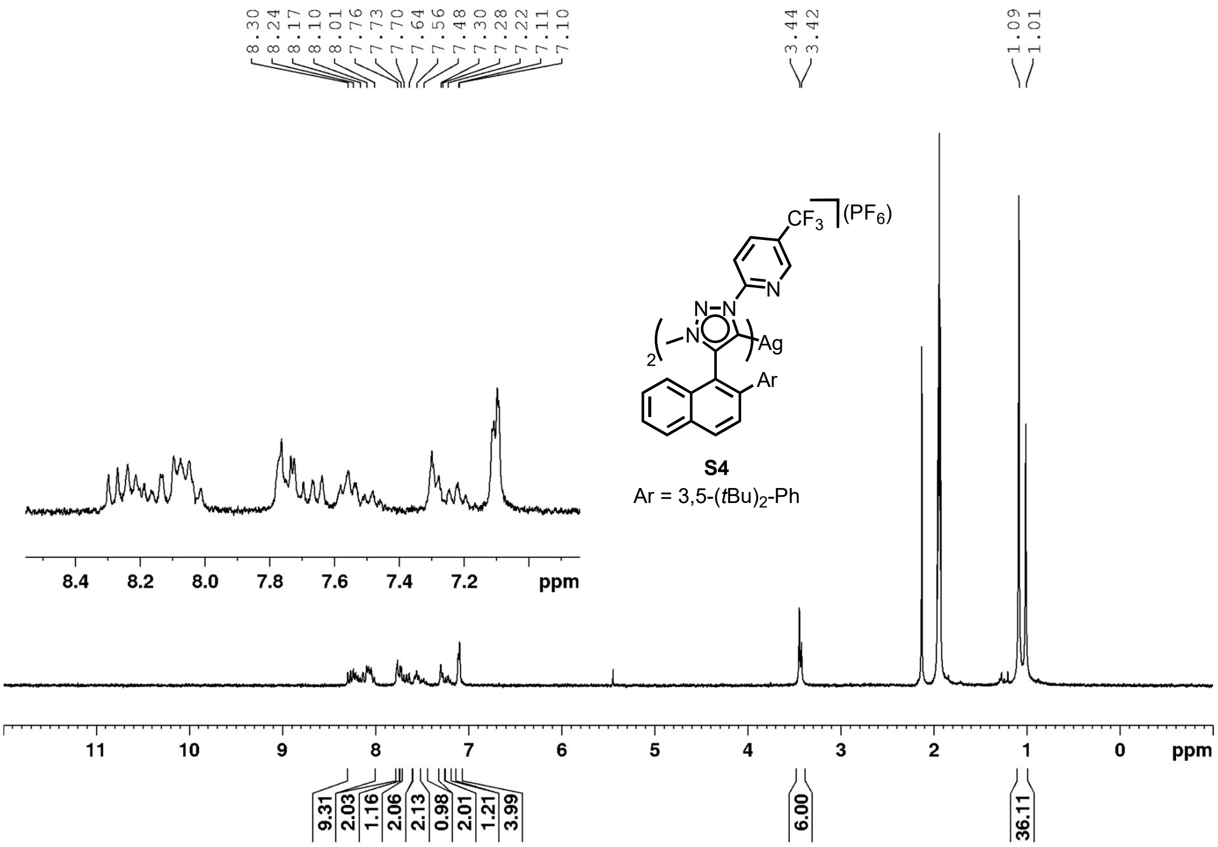

Figure S29: ^{1}H NMR spectrum of **S4** (300 MHz, CD_3CN, 300 K).

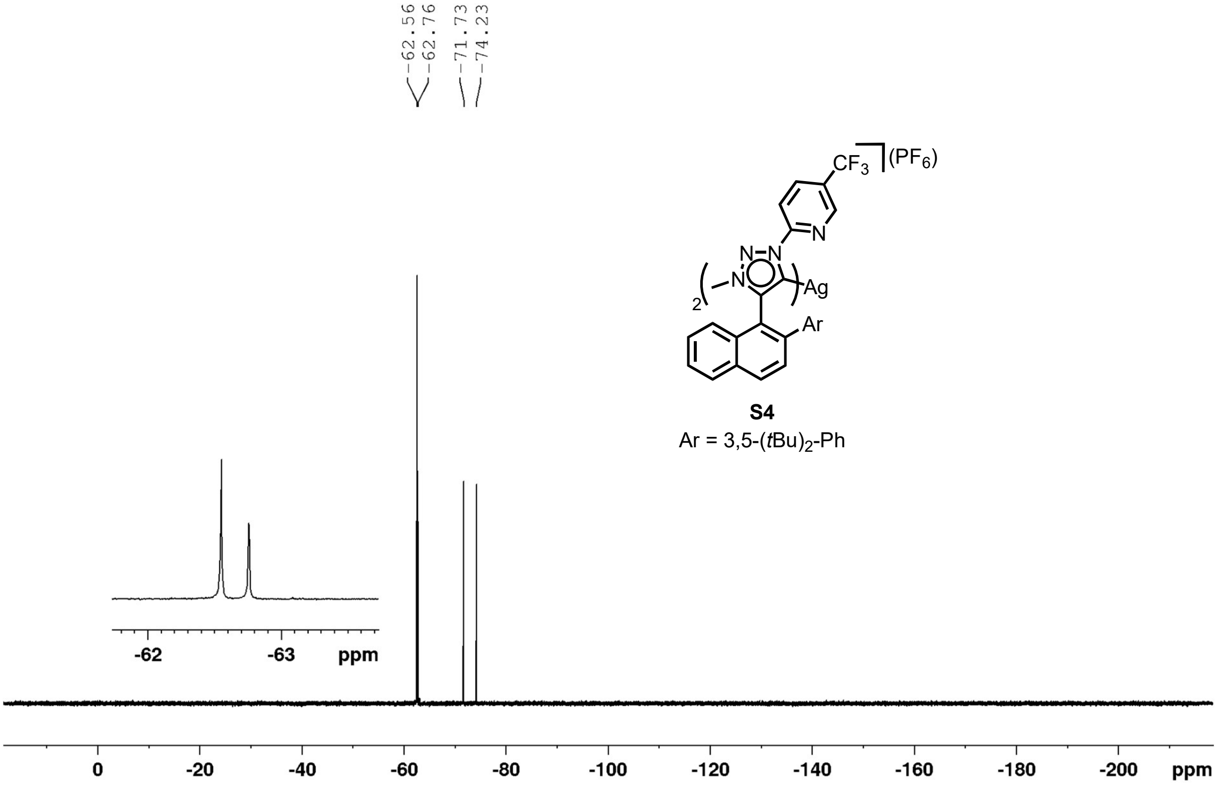

Figure S30: ^{19}F NMR spectrum of **S4** (282 MHz, CD_3CN, 300 K).

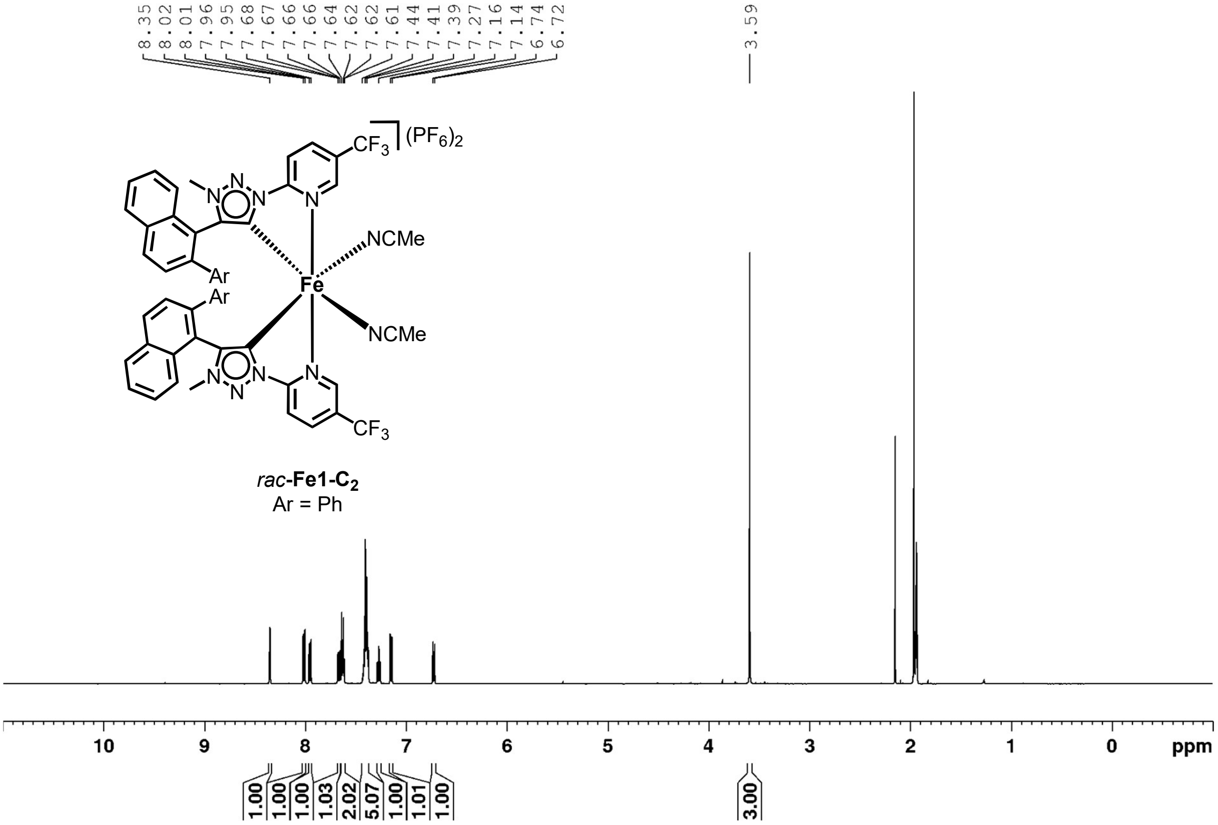

Figure S31: ^{1}H NMR spectrum of *rac*-**Fe1-C$_2$**-PF_6 (500 MHz, CD_3CN, 300 K).

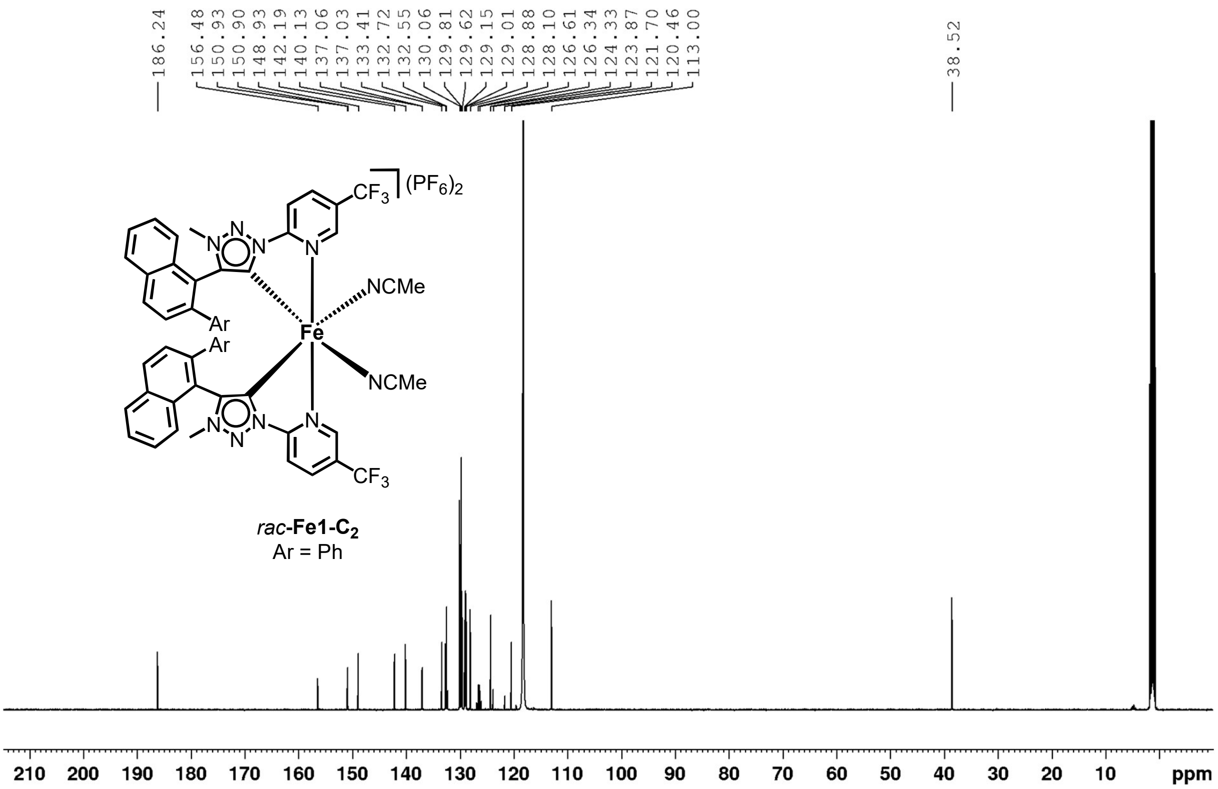

Figure S32: ^{13}C NMR spectrum of *rac*-**Fe1-C$_2$**-PF_6 (125 MHz, CD_3CN, 300 K).

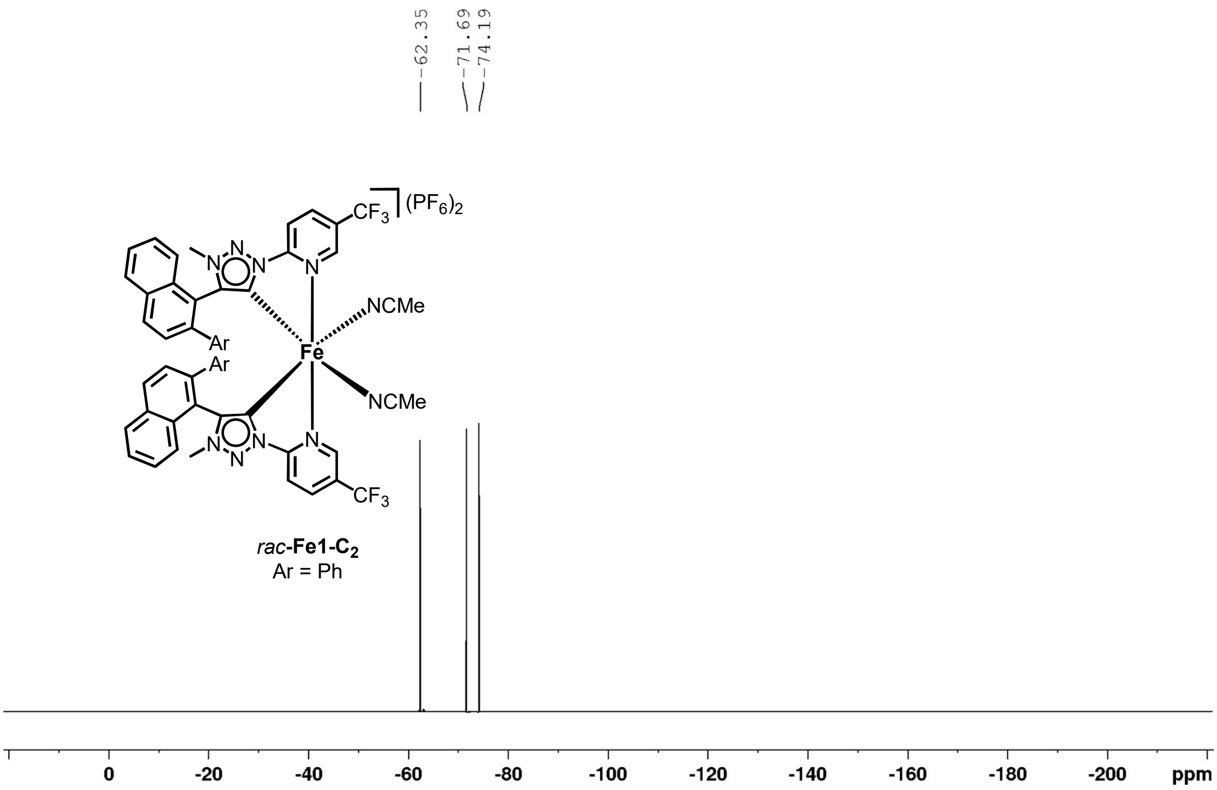

Figure S33: ^{19}F NMR spectrum of *rac*-**Fe1-C$_2$**-PF_6 (282 MHz, CD_3CN, 300 K).

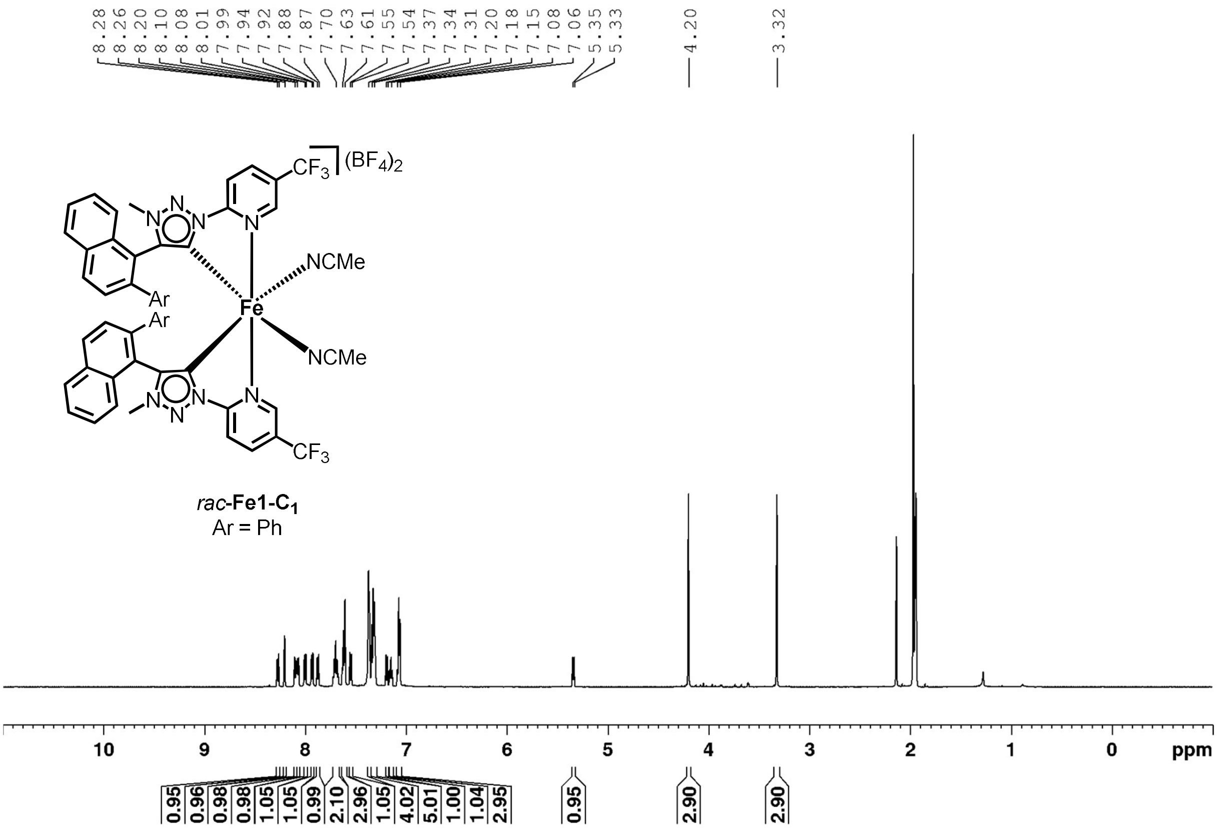

Figure S34: ^{1}H NMR spectrum of *rac*-**Fe1-C$_1$**-BF_4 (600 MHz, CD_3CN, 300 K).

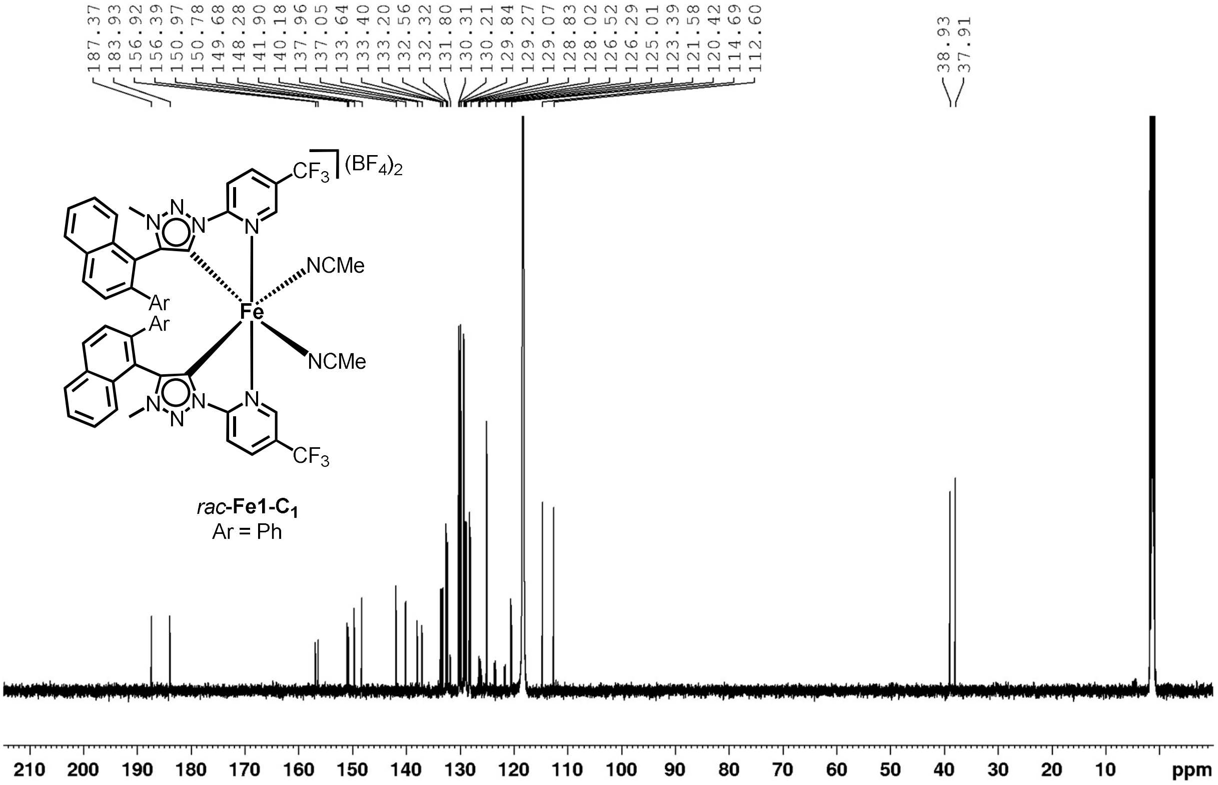

Figure S35: ^{13}C NMR spectrum of *rac*-**Fe1-C$_1$**-BF_4 (250 MHz, CD_3CN, 300 K).

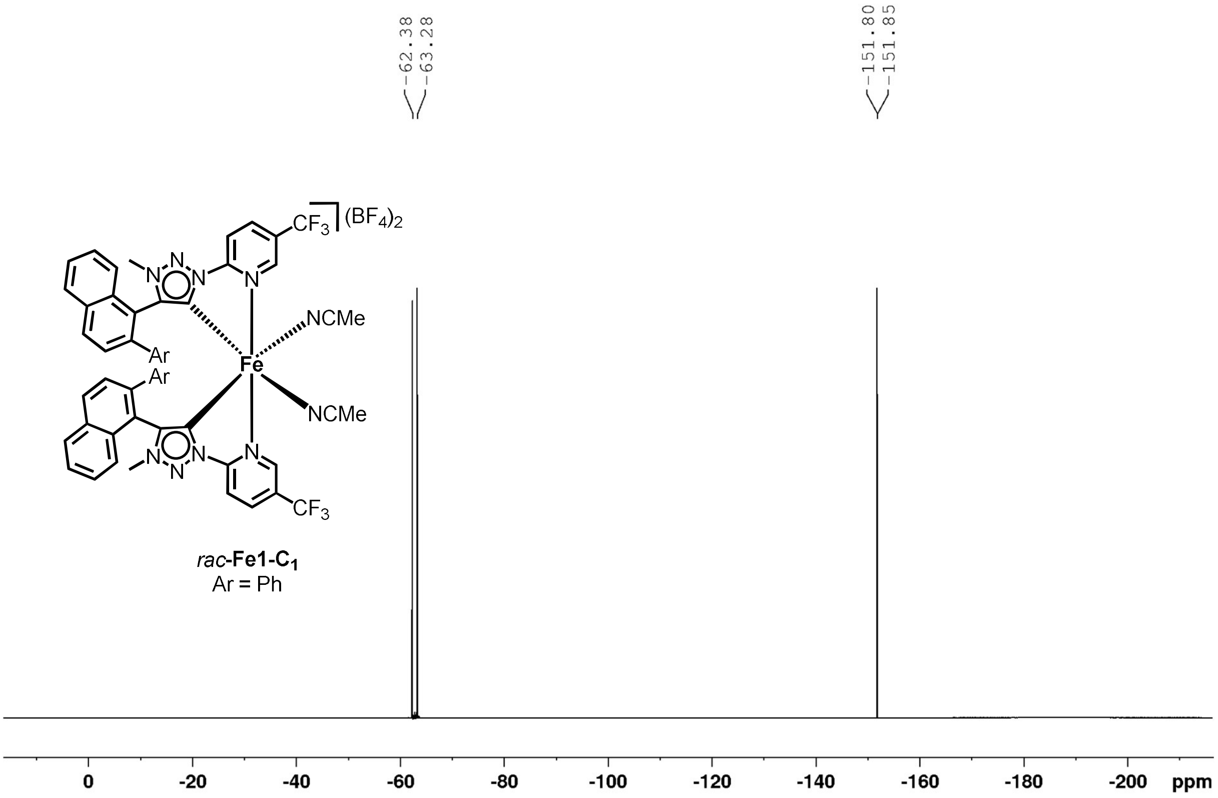

Figure S36: ^{19}F NMR spectrum of *rac*-**Fe1-C$_1$**-BF_4 (564 MHz, CD_3CN, 300 K).

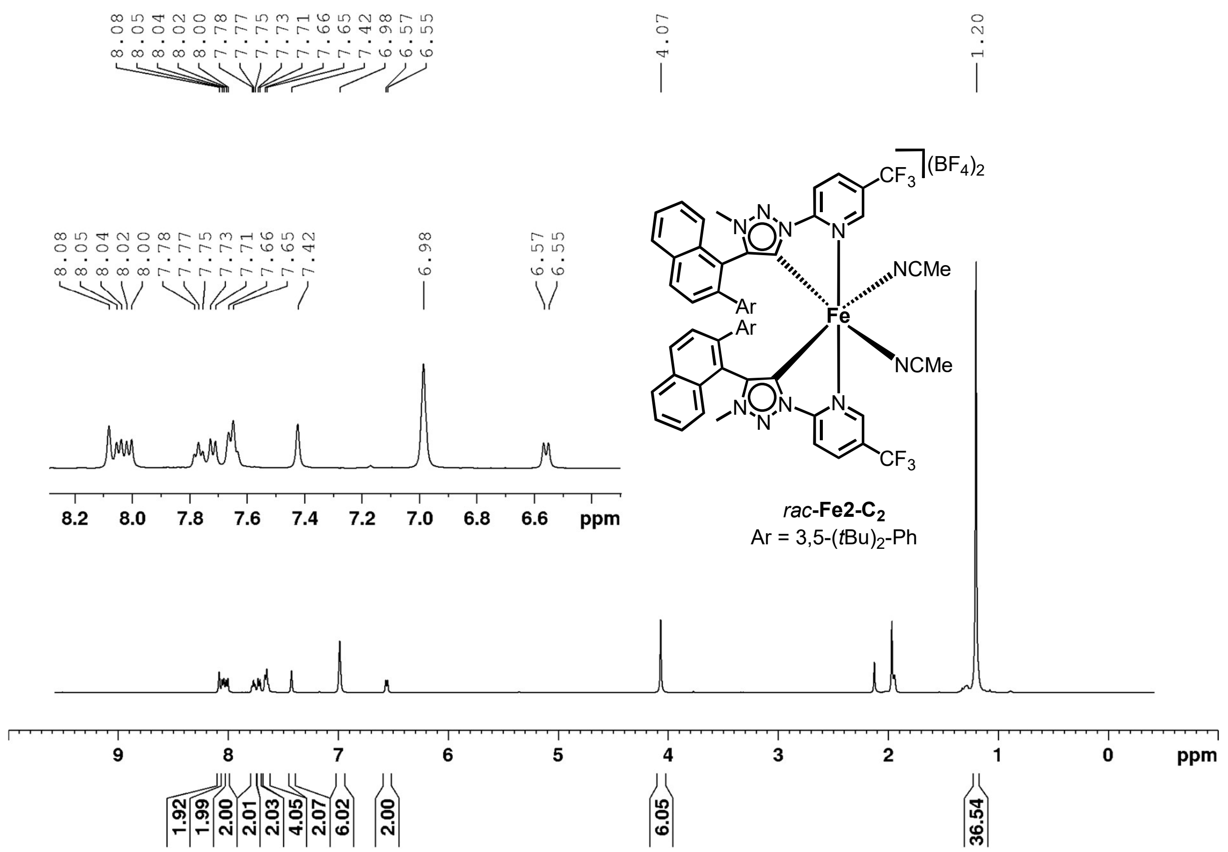

Figure S37: ^{1}H NMR spectrum of *rac*-**Fe2-C**$_2$-BF_4 (500 MHz, CD_3CN, 300 K).

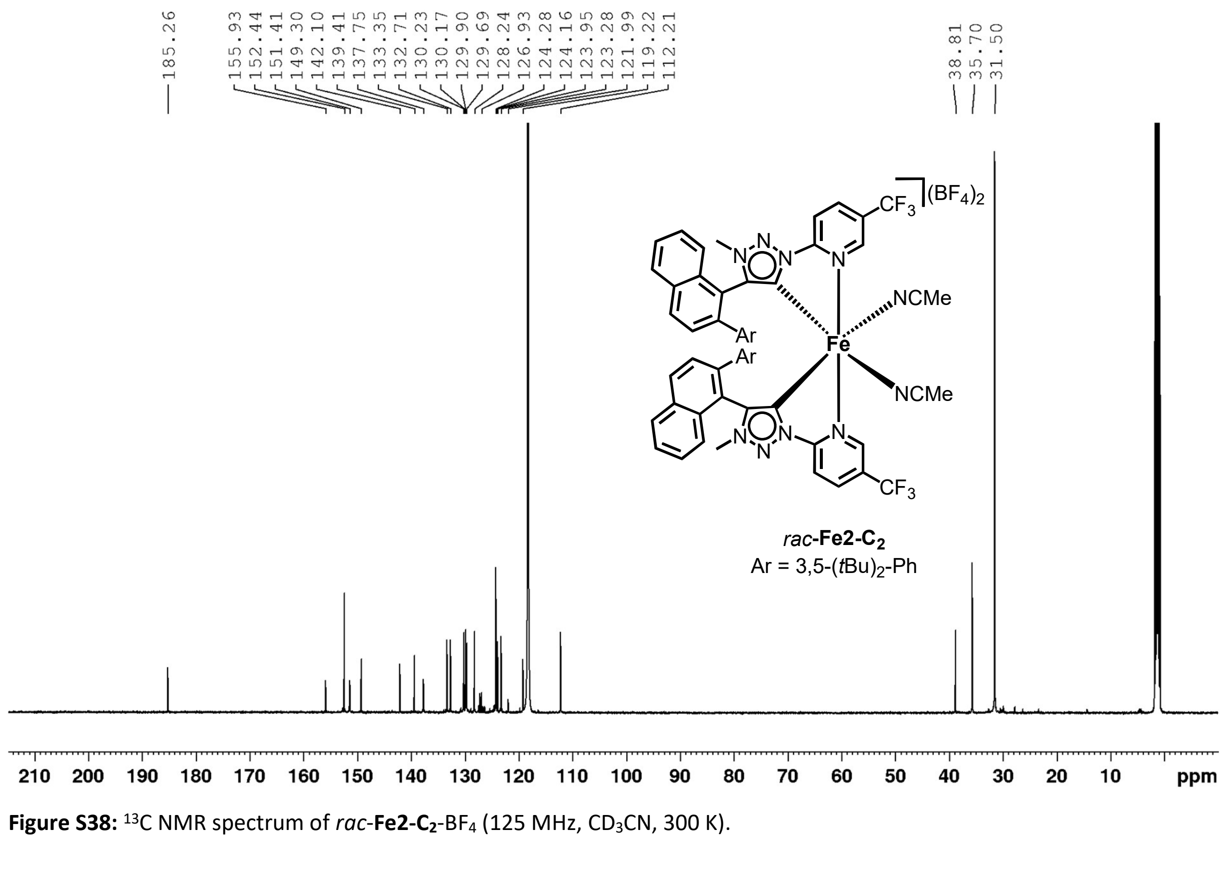

Figure S38: ^{13}C NMR spectrum of *rac*-**Fe2-C**$_2$-BF_4 (125 MHz, CD_3CN, 300 K).

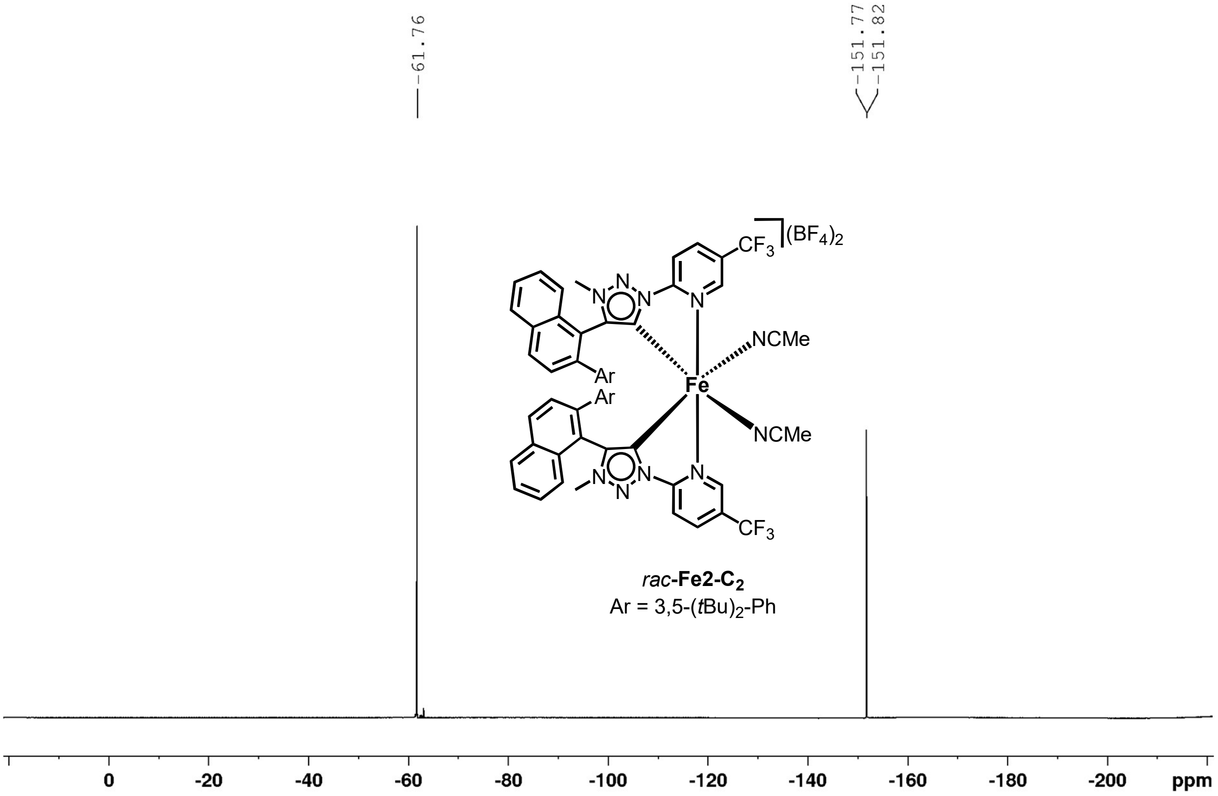

Figure S39: ^{19}F NMR spectrum of *rac*-**Fe2-C$_2$**-BF_4 (282 MHz, CD_3CN, 300 K).

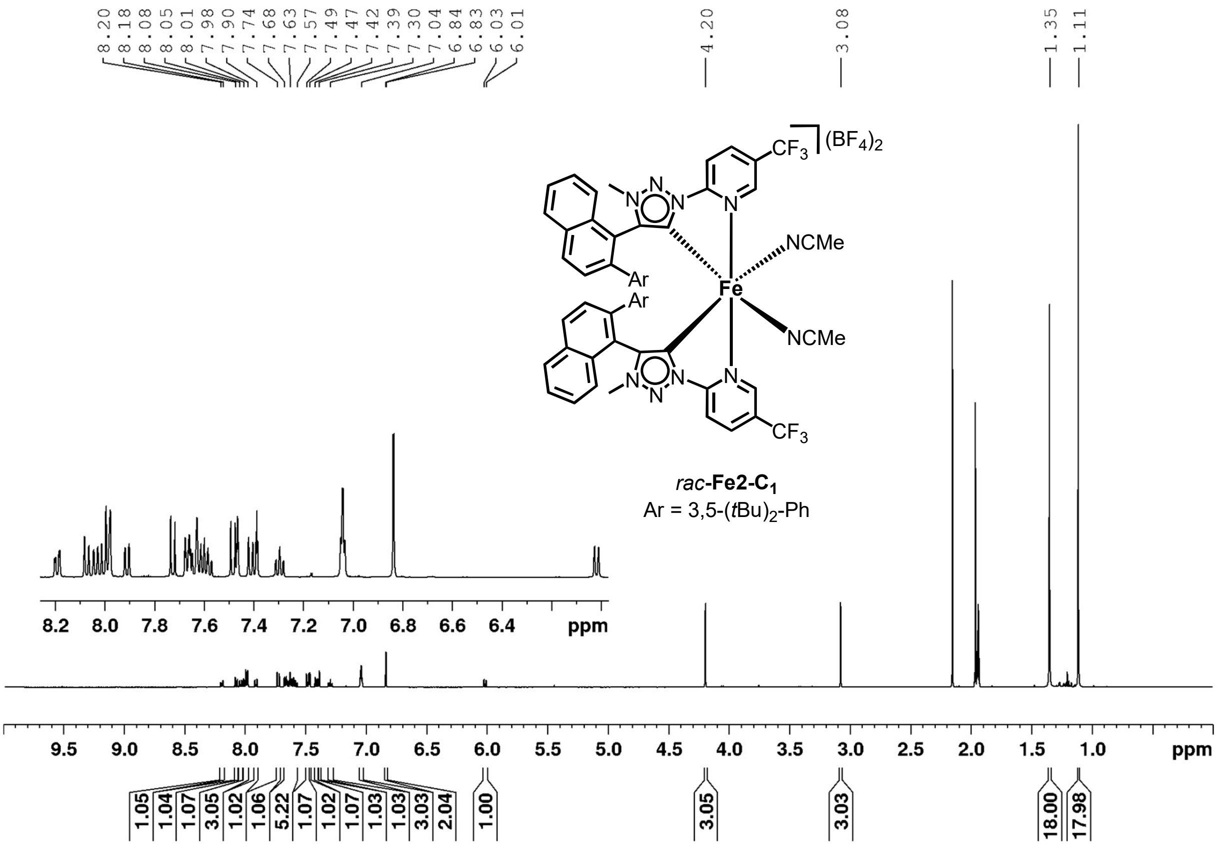

Figure S40: ^{1}H NMR spectrum of *rac*-**Fe2-C$_1$**-BF_4 (500 MHz, CD_3CN, 300 K).

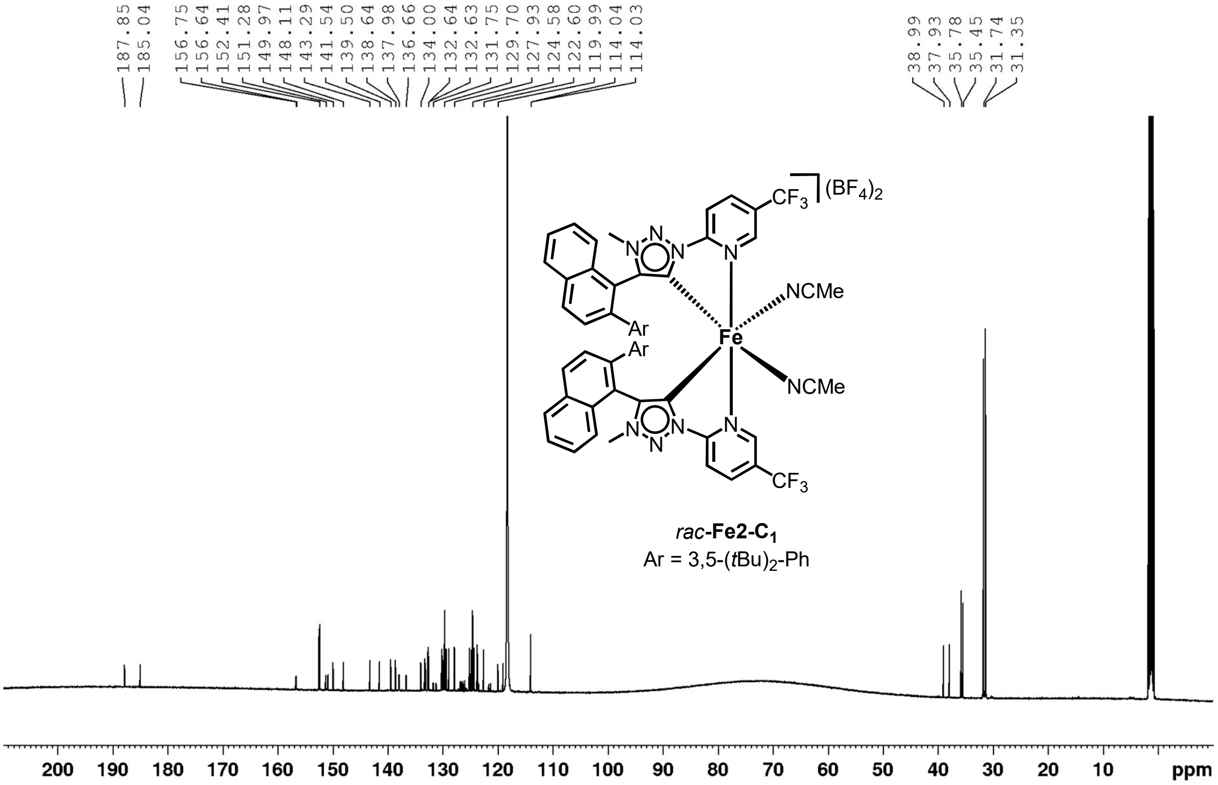

Figure S41: ^{13}C NMR spectrum of *rac*-**Fe2-C$_1$**-BF_4 (126 MHz, CD_3CN, 300 K).

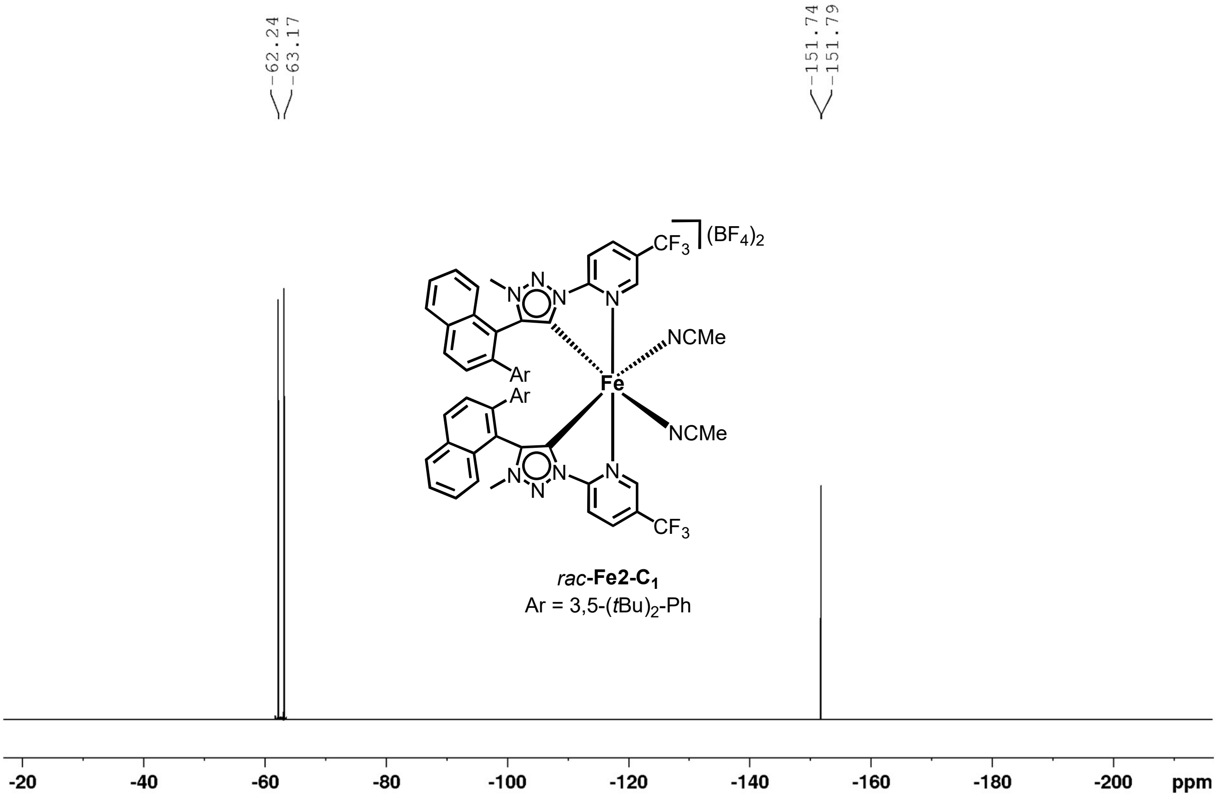

Figure S42: ^{19}F NMR spectrum of *rac*-**Fe2-C$_1$**-BF_4 (564 MHz, CD_3CN, 300 K).

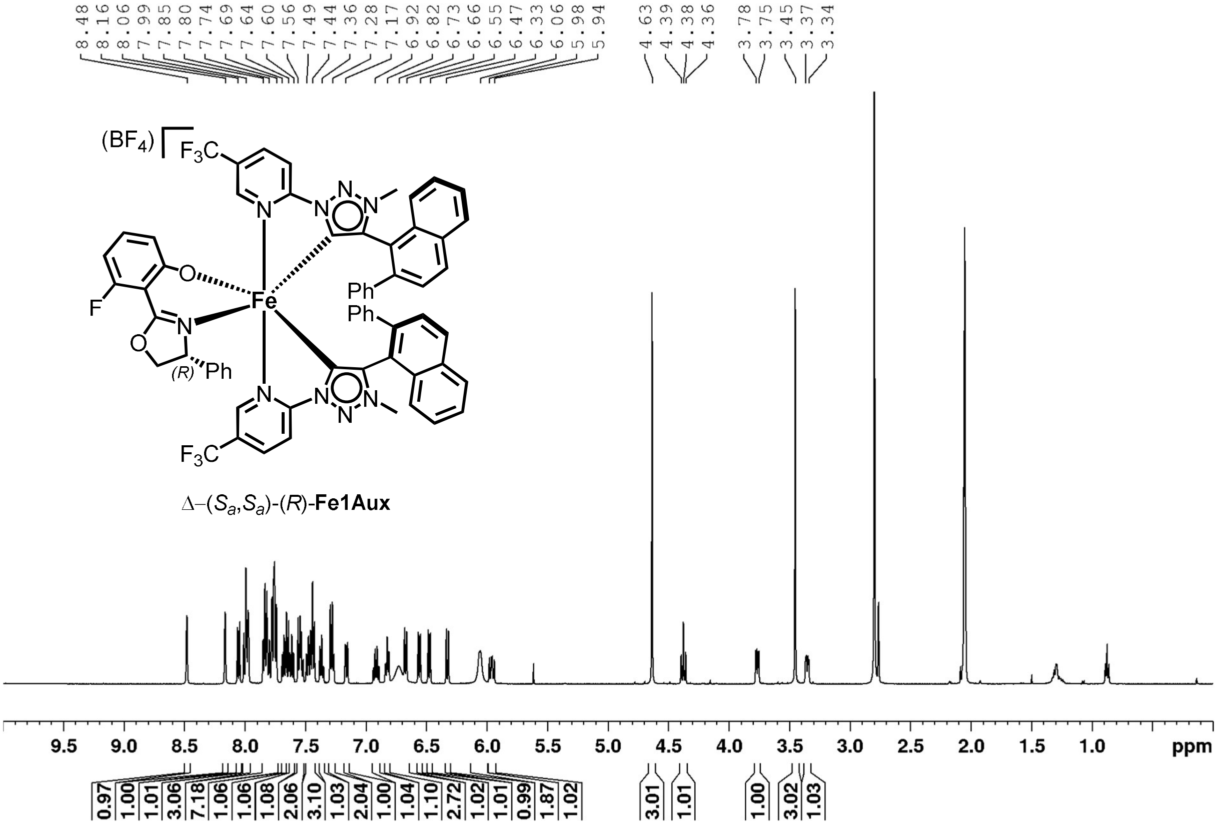

Figure S43: ^{1}H NMR spectrum of Δ-(S_a,S_a)-(R)-**Fe1Aux**-BF_4 (500 MHz, $(CD_3)_2CO$, 300 K).

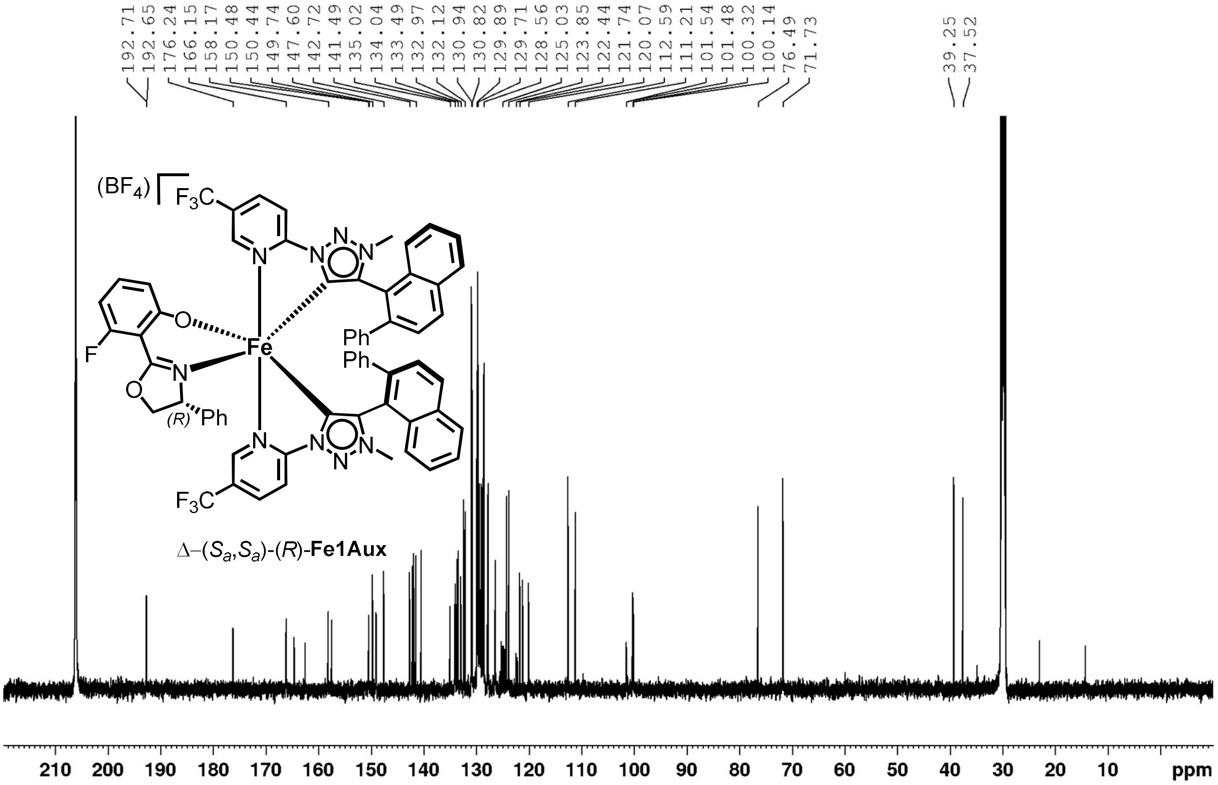

Figure S44: ^{13}C NMR spectrum of Δ-(S_a,S_a)-(R)-**Fe1Aux**-BF_4 (126 MHz, $(CD_3)_2CO$, 300 K).

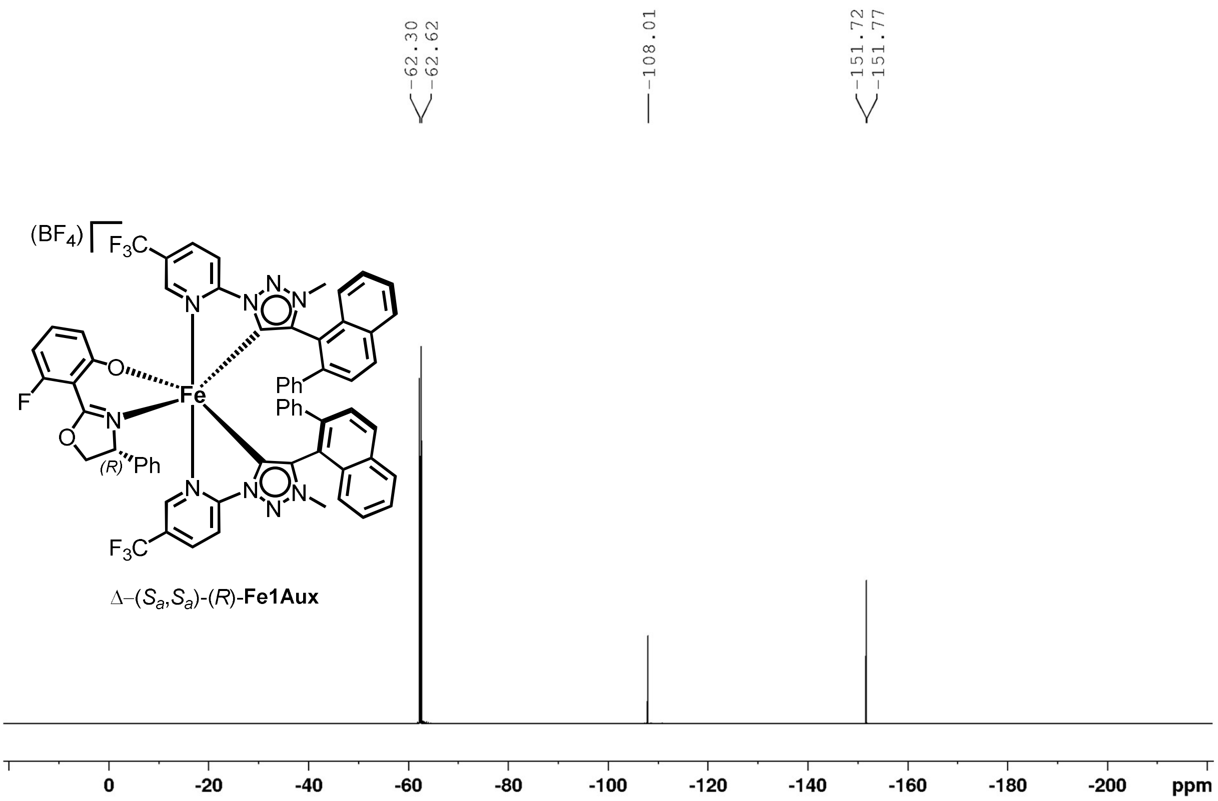

Figure S45: ^{19}F NMR spectrum of Δ-(S_a,S_a)-(R)-**Fe1Aux**-BF_4 (282 MHz, $(CD_3)_2CO$, 300 K).

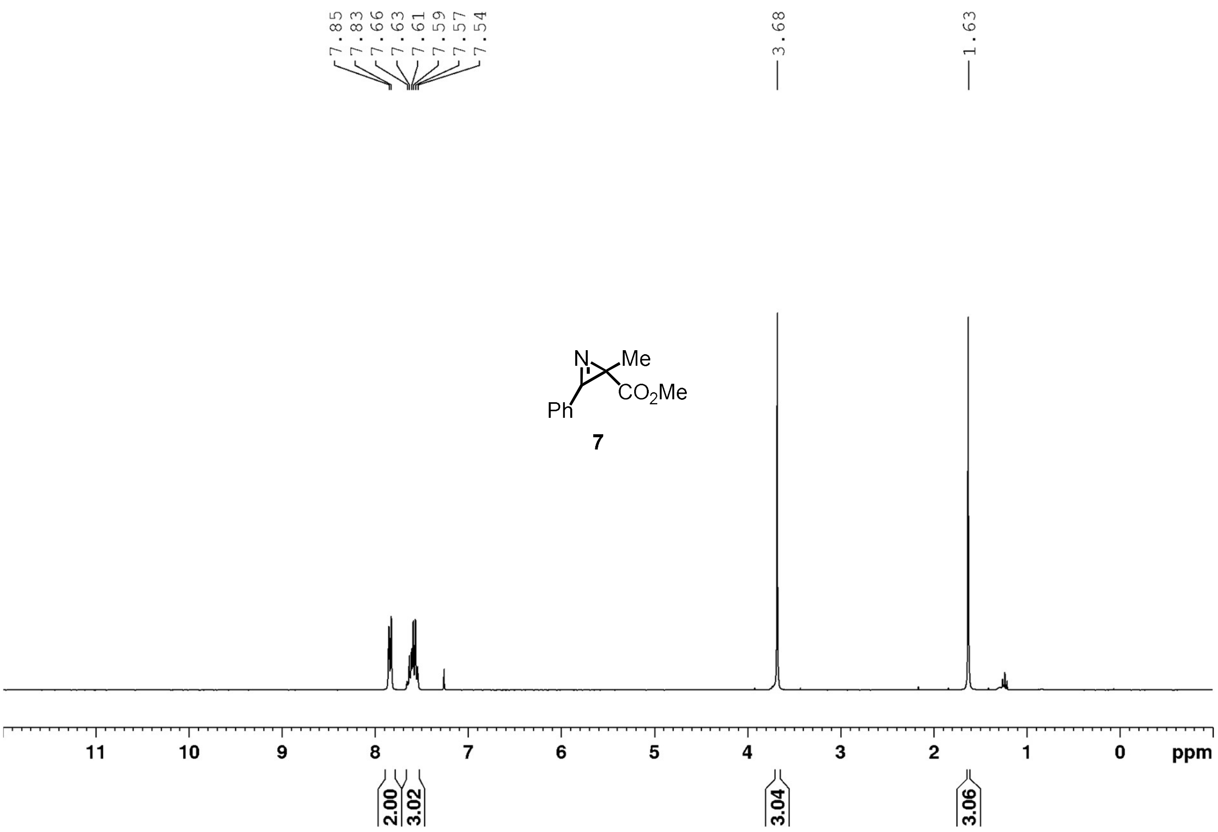

Figure S46: ^{1}H NMR spectrum of **7** (300 MHz, CD_2Cl_2, 300 K).

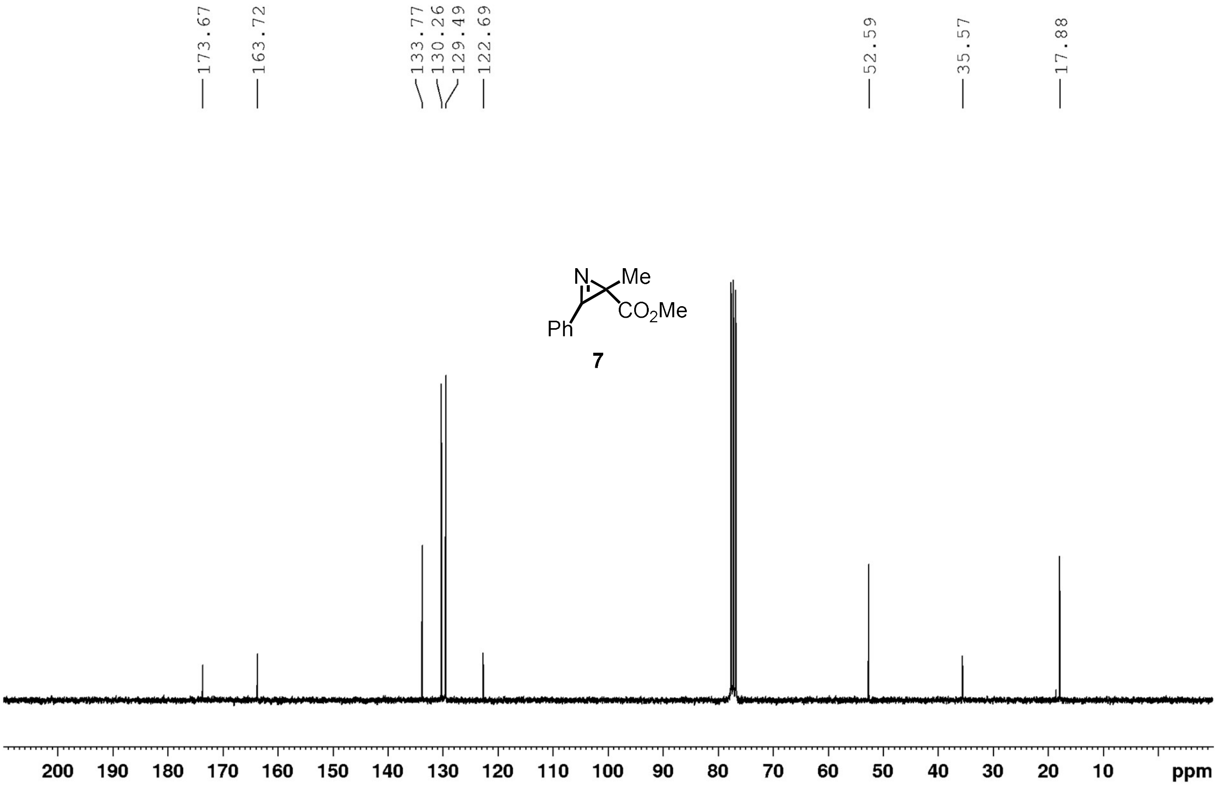

Figure S47: ^{13}C NMR spectrum of **7** (75 MHz, CD_2Cl_2, 300 K).

10 Chiral HPLC Traces

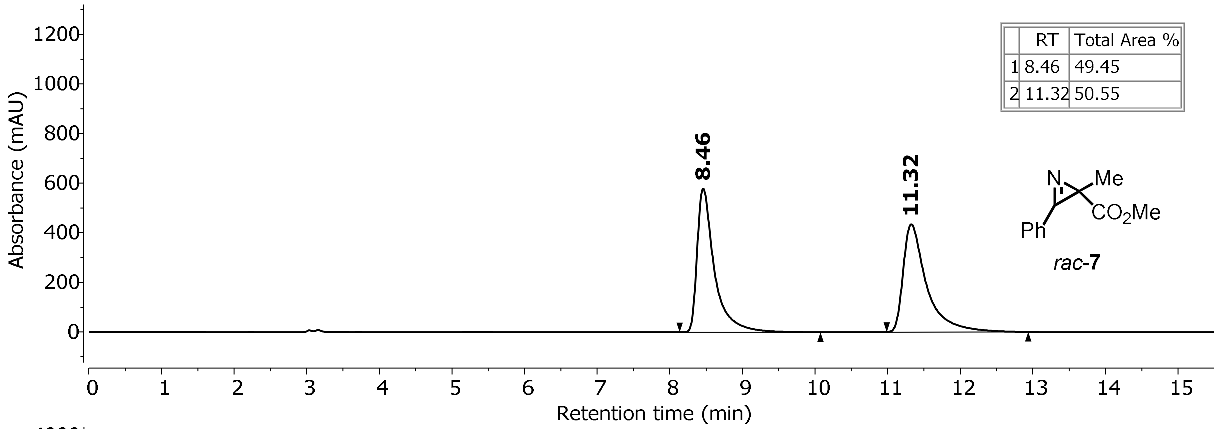

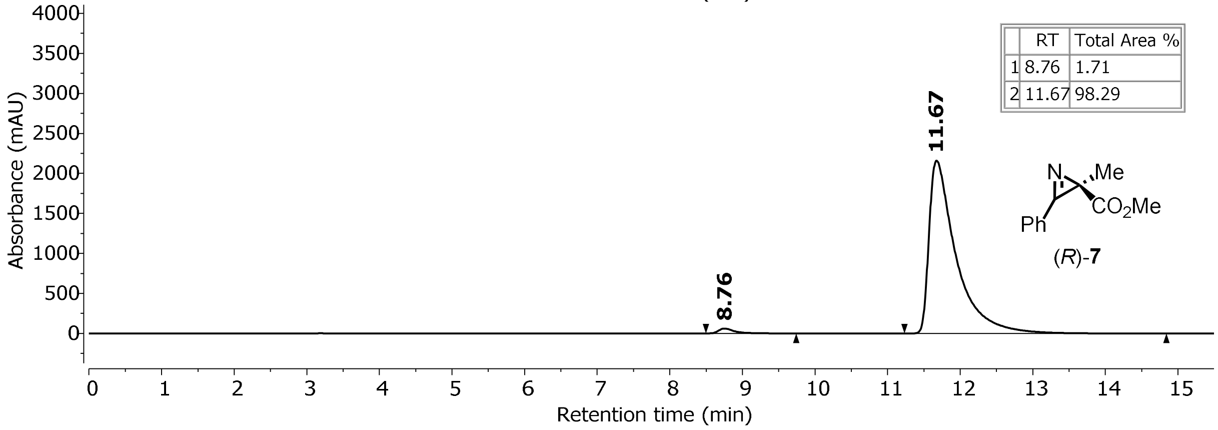

Figure S48: HPLC chromatogram of top: *rac*-**7** and bottom: (*R*)-**7** with 96.6% ee.

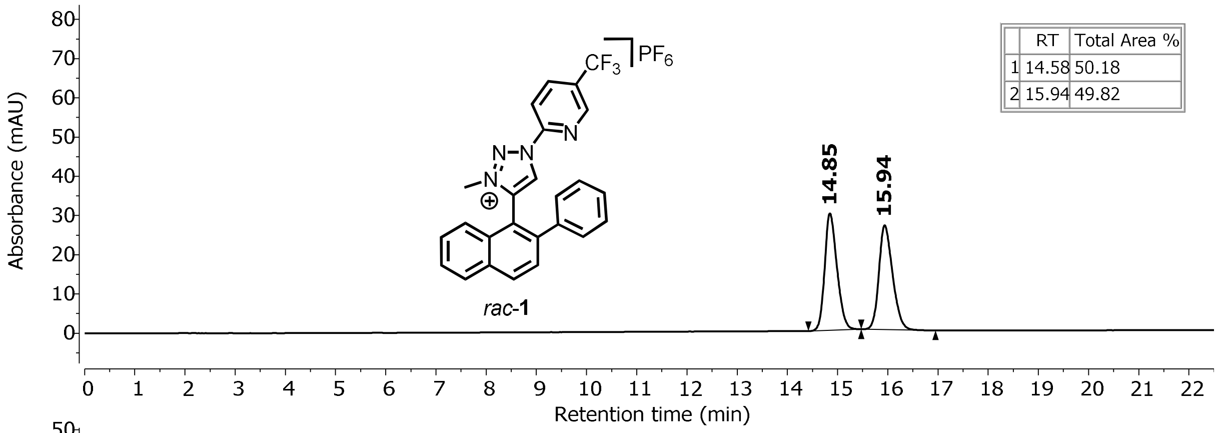

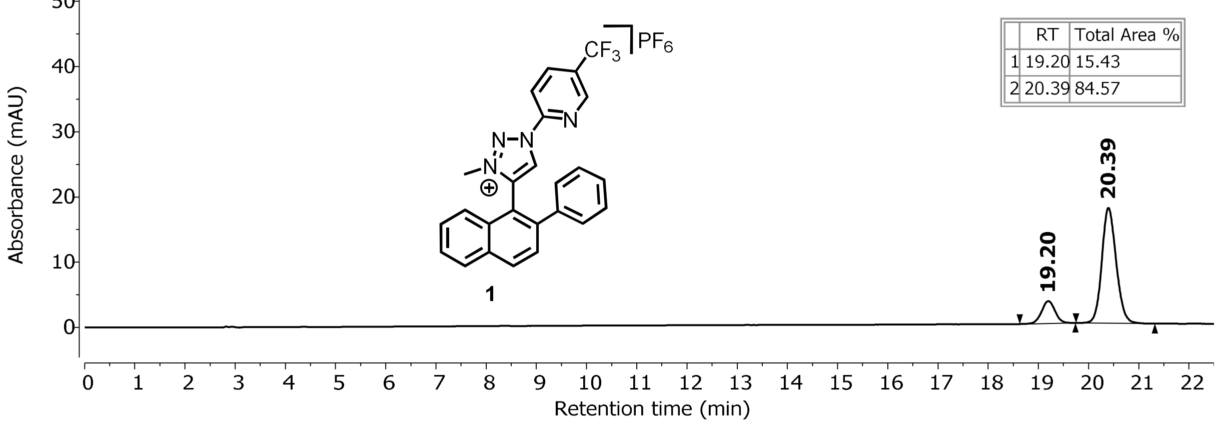

Figure S49: HPLC chromatogram of top: *rac*-**1** and bottom: decay measurement of 1 after 20 min; 69% ee.

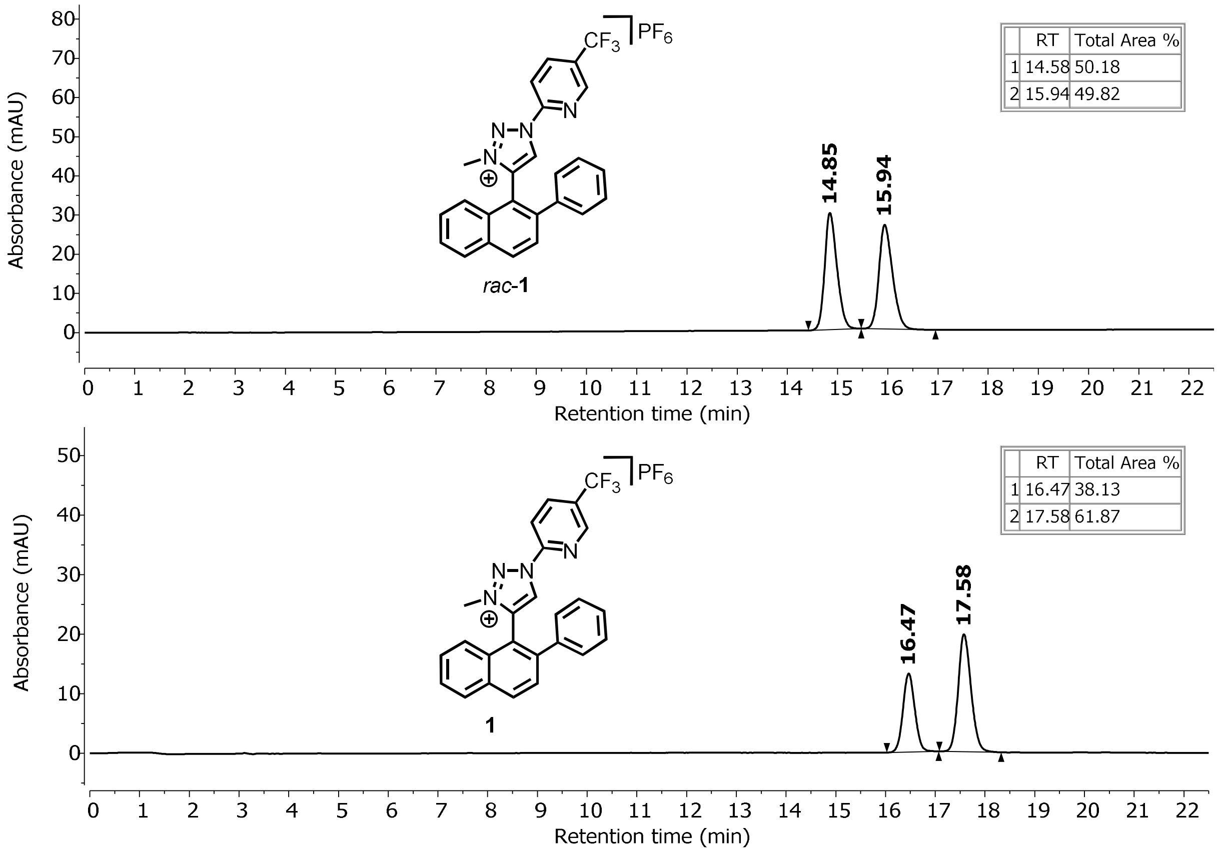

Figure S50: HPLC chromatogram of <u>top</u>: *rac*-**1** and <u>bottom</u>: decay measurement of 1 after 430 min; 24% ee.

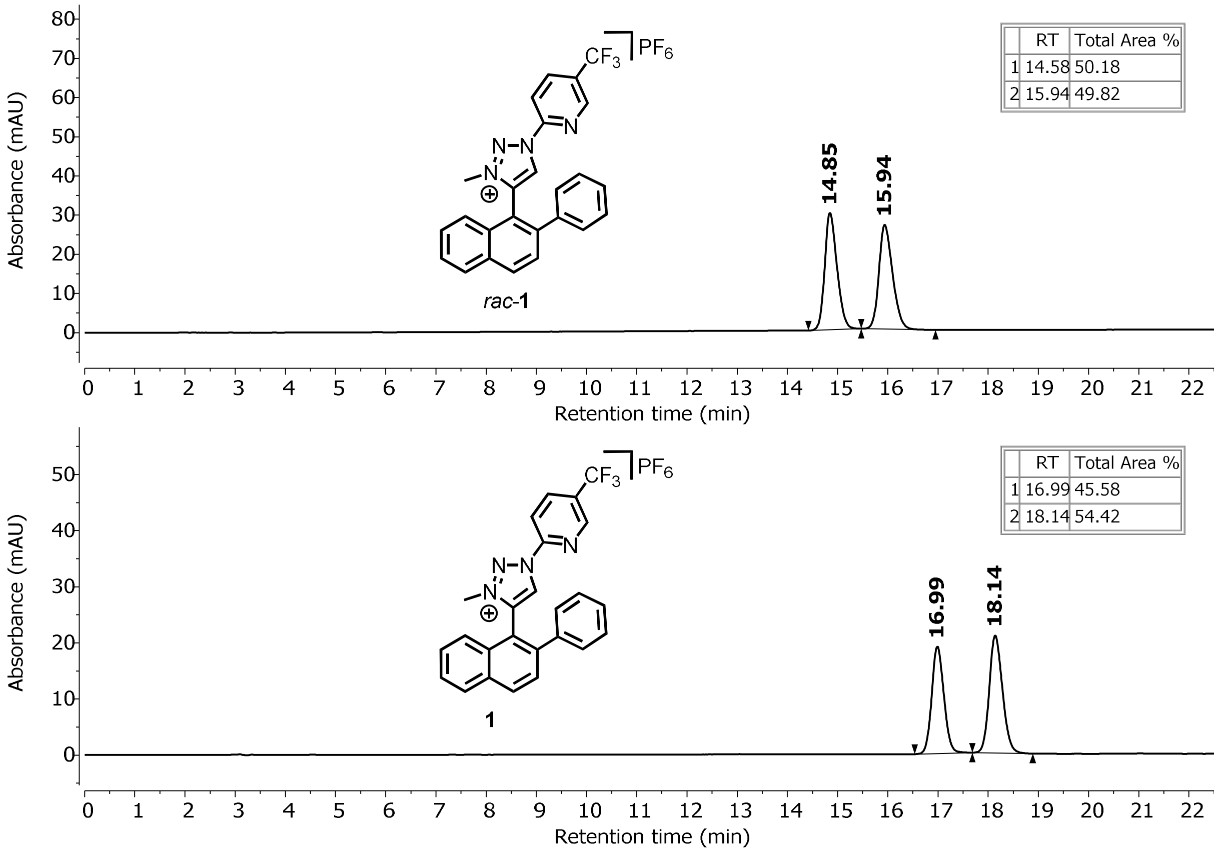

Figure S51: HPLC chromatogram of <u>top</u>: *rac*-**1** and <u>bottom</u>: decay measurement of 1 after 790 min; 8.8% ee.

11 CD-Spectra

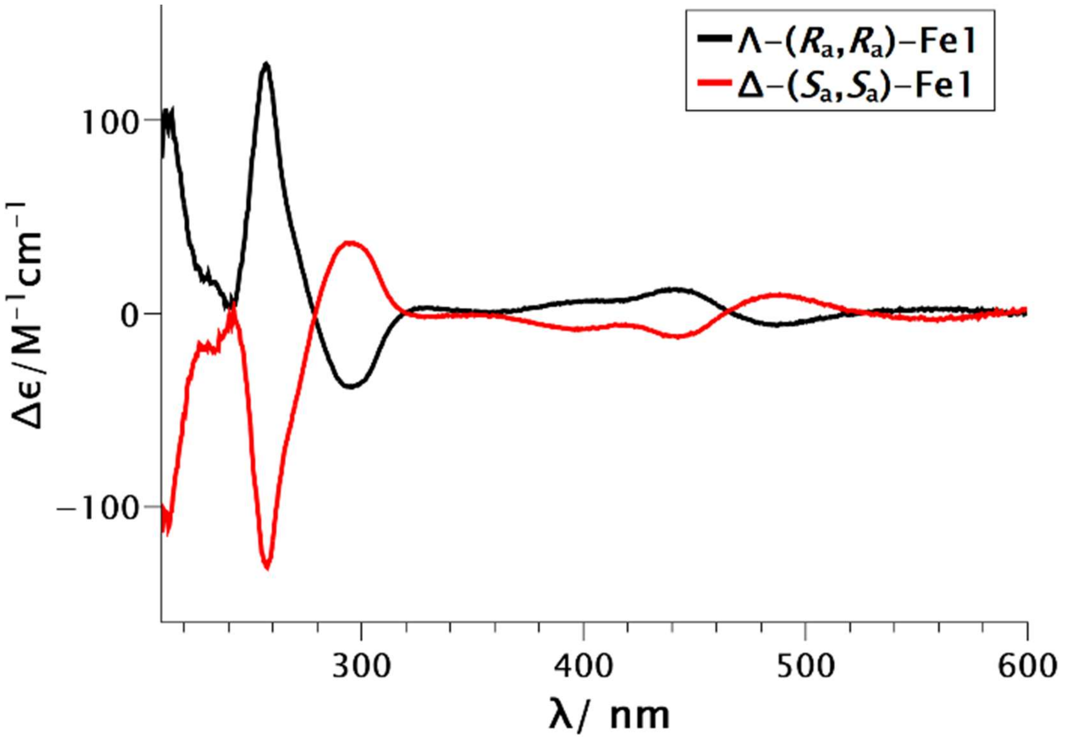

Figure S52: CD-spectra of Λ-(R_a,R_a)-**Fe1** and Δ-(S_a,S_a)-**Fe1** in MeCN (0.25 mM).

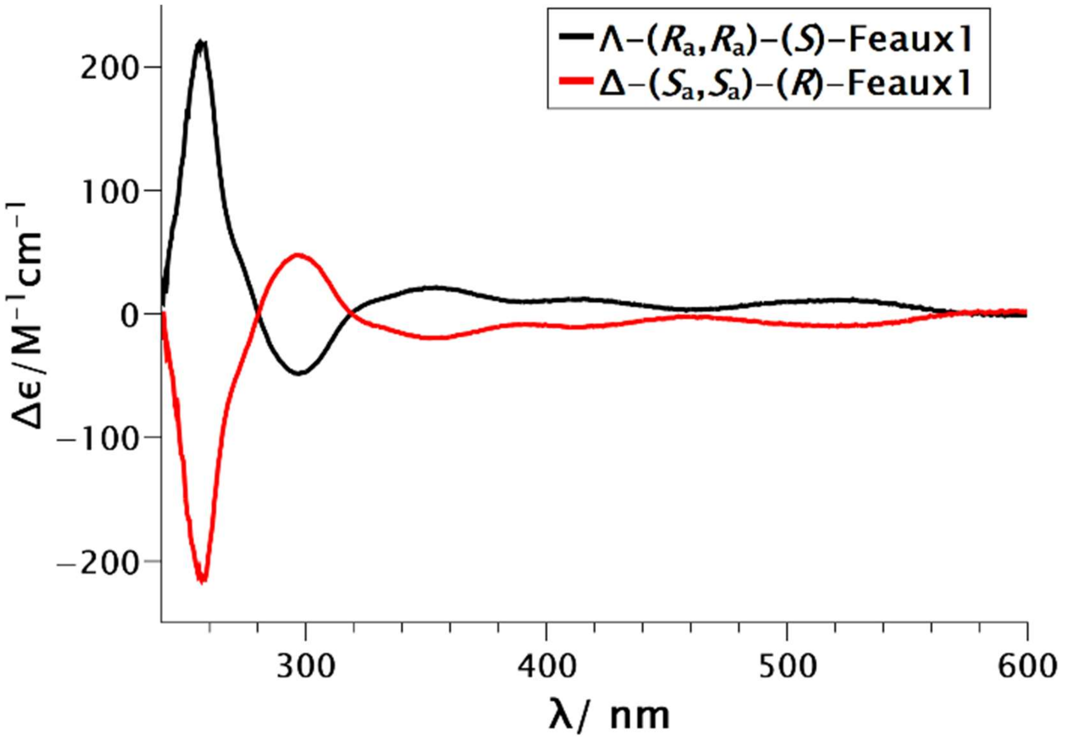

Figure S53: CD-spectra of Λ-(R_a,R_a)-(S)-**Fe1Aux** and Δ-(S_a,S_a)-(R)-**Fe1Aux** in CH_2Cl_2 (0.25 mM).

12 Structure elucidation of C_2-Isomer and C_1-Isomer for Fe1 and Fe2

12.1 Relation of Possible Isomers

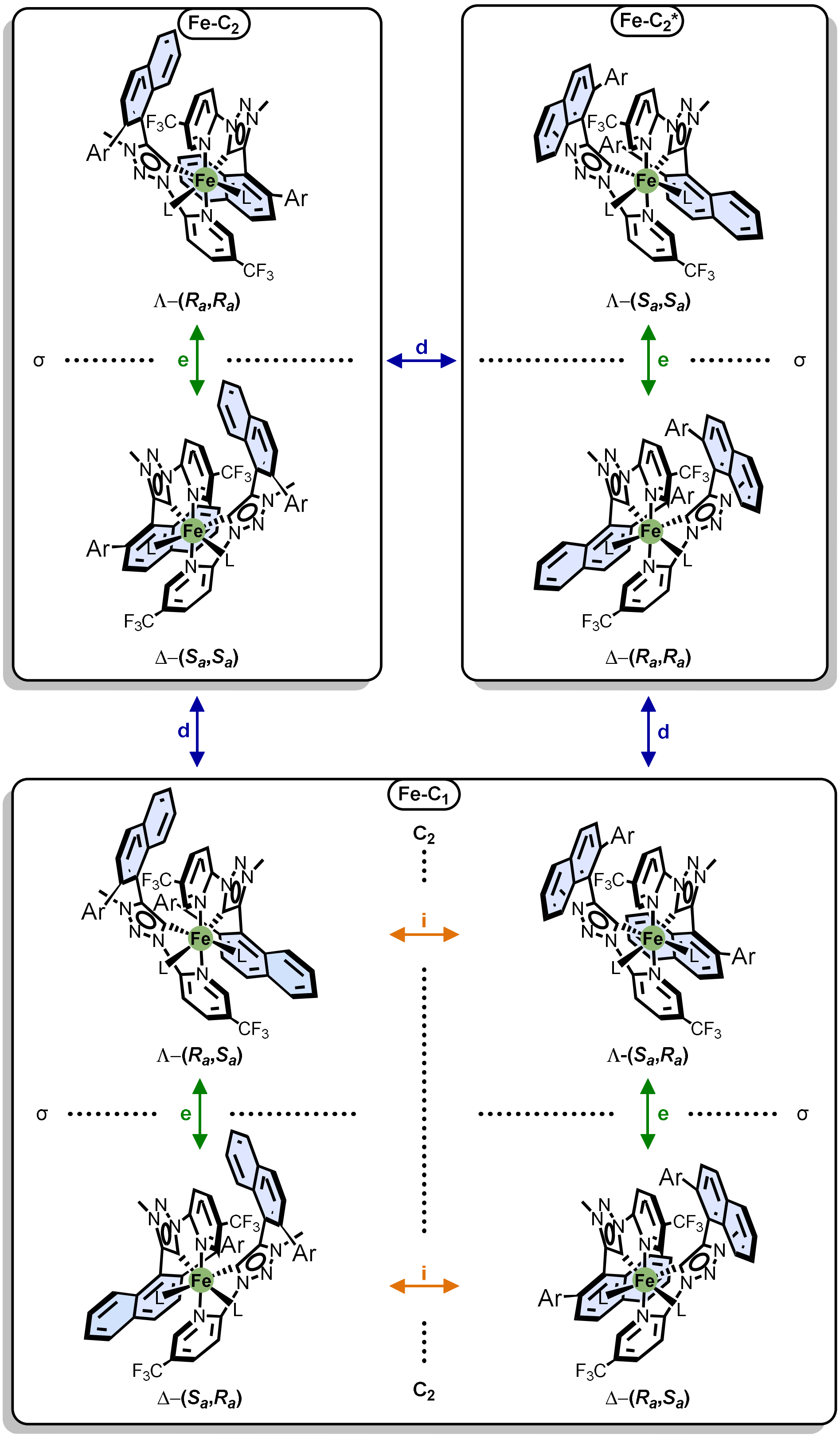

Figure S54: Relation of the possible isomer of **Fe1** and **Fe2**. Anions are omitted for clarity. e: Enantiomers. d: Diastereomers. i: Identical molecule.

12.2 Single Crystal X-Ray Diffraction

Crystal-growth

Single crystals of *rac*-**Fe1-C**$_1$ and *rac*-**Fe2-C**$_2$. suitable for X-ray diffraction were obtained by slow diffusion from a solution of the respective compound (2 mg) in CH_3CN, which was layered with diethyl ether at room temperature in an NMR tube. Crystals were obtained after 3-4 days.

X-ray diffraction

***rac*-Fe1-C$_1$**

A suitable crystal of $C_{54}H_{40}F_6FeN_{10} \cdot 2\ PF_6$ was selected under inert oil and mounted using a MiTeGen loop. Intensity data of the crystal were recorded with a STADIVARI diffractometer (Stoe & Cie). The diffractometer was operated with Mo-Kα radiation (0.71073 Å, microfocus source) and equipped with a Dectris PILATUS 300K detector. Evaluation, integration and reduction of the diffraction data was carried out using the X-Area software suite.[15] Multi-scan and numerical absorption corrections were applied with the LANA and X-RED32 modules of the X-Area software suite.[16,17] The structure was solved using dual-space methods (SHELXT-2018/2) and refined against F^2 (SHELXL-2019/1 using ShelXle interface).[18-20] All non-hydrogen atoms were refined with anisotropic displacement parameters. One PF_6^- anion and one CF_3 group were refined disordered using the DSR plugin[21] in the ShelXle software. The hydrogen atoms were refined using the "riding model" approach with isotropic displacement parameters 1.2 times (1.5 times for the methyl groups) of that of the preceding carbon atom. CCDC 2400043 contains the supplementary crystallographic data for this paper. These data can be obtained free of charge from the Cambridge Crystallographic Data Centre via www.ccdc.cam.ac.uk/structures.

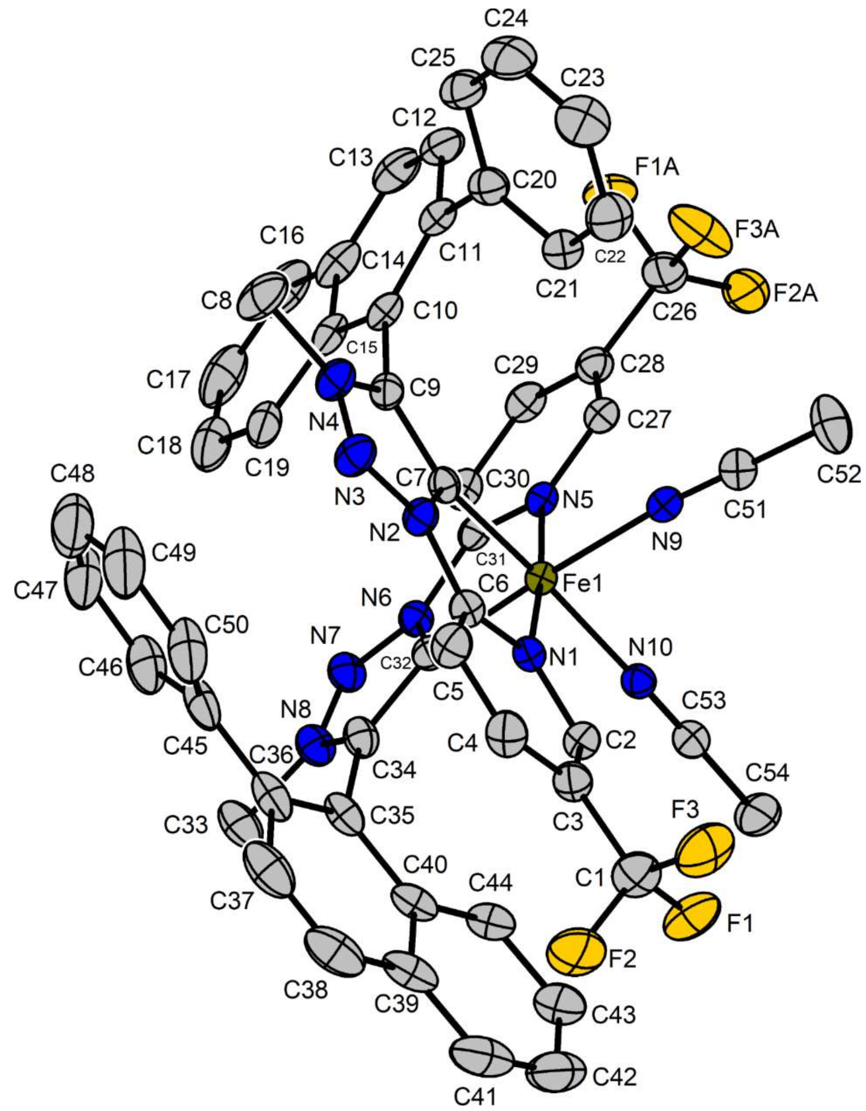

Figure S55: Crystal structure of *rac*-**Fe1-C₁**. Only one enantiomer is shown. The PF_6^- anions and the hydrogen atoms are not shown. The disorder of the CF_3 group is not shown. Displacement ellipsoids are shown at 50 % probability level at 100 K.

Table S6: Selected crystallographic data and details of the structure determination for $C_{54}H_{40}F_6FeN_{10}\cdot 2\ PF_6$: *rac*-**Fe1-C₁**.

Identification code	HBB56
Empirical formula	$C_{54}H_{40}F_{18}FeN_{10}P_2$
Molar mass / g·mol^{-1}	1288.75
Space group (No.)	$P2_1/n$ (14)
a / Å	19.8794(4)
b / Å	13.1256(2)
c / Å	24.0253(5)
β / °	98.267(2)
V / Å^3	6203.8(2)
Z	4
$\rho_{calc.}$ / g·cm^{-3}	1.380
μ / mm^{-1}	0.392
Color	red

Crystal habitus	block
Crystal size / mm^3	0.325 x 0.196 x 0.144
T / K	100
Λ / Å	0.71073 (Mo-K$_\alpha$)
ϑ range / °	2.311 to 28.000
Range of Miller indices	$-26 \leq h \leq 26$
	$-15 \leq k \leq 17$
	$-31 \leq l \leq 31$
Absorption correction	multi-scan and numerical
T_{min}, T_{max}	0.8810, 0.9454
R_{int}, R_σ	0.0620, 0.0457
Completeness of the data set	0.999
No. of measured reflections	91557
No. of independent reflections	14971
No. of parameters	862
No. of restraints	597
S (all data)	1.070
$R(F)$ ($I \geq 2\sigma(I)$, all data)	0.0416, 0.0693
$wR(F^2)$ ($I \geq 2\sigma(I)$, all data)	0.1001, 0.1088
Extinction coefficient	not refined
$\Delta\rho_{max}$, $\Delta\rho_{min}$ / e·Å^{-3}	0.359, –0.256

rac-**Fe1-C$_1$**

A suitable crystal of $C_{70}H_{72}F_6FeN_{10} \cdot 2\ PF_6 \cdot CH_3CN$ was selected under inert oil and mounted using a MiTeGen loop. Intensity data of the crystal were recorded with a STADIVARI diffractometer (Stoe & Cie). The diffractometer was operated with Cu-Kα radiation (1.54186 Å, microfocus source) and equipped with a Dectris PILATUS 300K detector. Evaluation, integration and reduction of the diffraction data was carried out using the X-Area software suite.[15] Multi-scan and numerical absorption corrections were applied with the LANA and X-RED32 modules of the X-Area software suite.[16,17]The structure was solved using dual-space methods (SHELXT-2018/2) and refined against F^2 (SHELXL-2019/1 using ShelXle interface).[18-20] All non-hydrogen atoms were refined with anisotropic displacement parameters. The hydrogen atoms were refined using the “riding model” approach with isotropic displacement parameters 1.2 times (1.5 times for the methyl groups) of that of the preceding carbon atom. CF_3 and *tert*-butyl groups as well as both PF_6^- anions were refined disordered using the DSR plugin.[21] The residual electron density belonging to disordered solvent molecules was eliminated using the SQUEEZE algorithm in the PLATON software.[22,23] CCDC 2400042 contains the supplementary crystallographic data for this paper. These data can be obtained free of charge from the Cambridge Crystallographic Data Centre via www.ccdc.cam.ac.uk/structures.

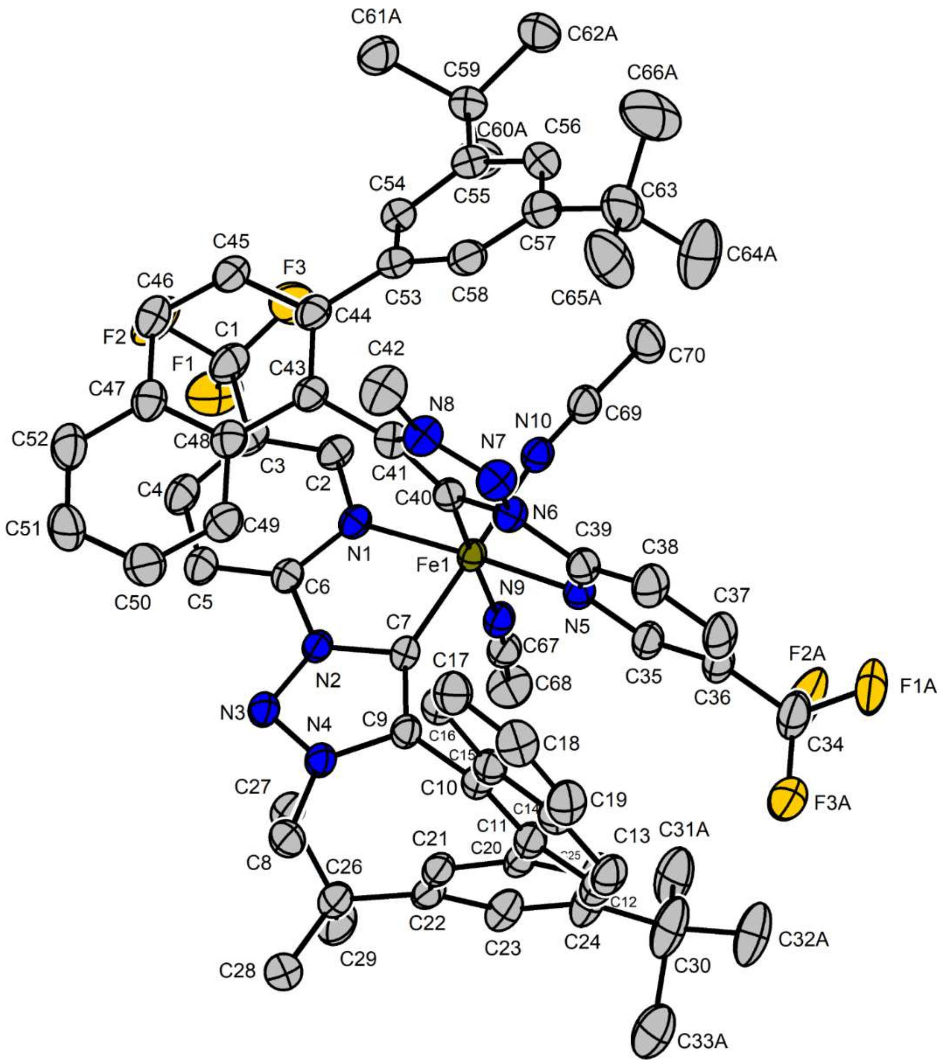

Figure S56: Crystal structure of *rac*-**Fe2-C$_2$**. Only one enantiomer is shown. The PF_6^- anions, the acetonitrile solvent molecule and the hydrogen atoms are not shown. No disorder is shown. Displacement ellipsoids are shown at 50 % probability level at 100 K.

Table S7: Selected crystallographic data and details of the structure determination for $C_{70}H_{72}F_6FeN_{10} \cdot 2\ PF_6 \cdot CH_3CN$: *rac*-**Fe2-C$_2$**.

Identification code	MBB72C2rac
Empirical formula	$C_{72}H_{75}F_{18}FeN_{11}P_2$
Molar mass / g·mol^{-1}	1554.21
Space group (No.)	$P\bar{1}$ (2)
a / Å	12.86620(10)
b / Å	15.8429(2)
c / Å	20.5929(2)
α / °	70.3280(10)
β / °	85.4340(10)
γ / °	76.2540(10)
V / Å^3	3839.41(7)
Z	2
$\rho_{calc.}$ / g·cm^{-3}	1.344

μ / mm^{-1}	2.769
Color	red
Crystal habitus	needle
Crystal size / mm^3	0.164 x 0.072 x 0.034
T / K	100
Λ / Å	1.54186 (Cu-K$_\alpha$)
ϑ range / °	3.040 to 77.316
Range of Miller indices	$-15 \leq h \leq 16$
	$-19 \leq k \leq 12$
	$-26 \leq l \leq 25$
Absorption correction	multi-scan and numerical
T_{min}, T_{max}	0.6370, 0.9107
R_{int}, R_{σ}	0.0411, 0.0421
Completeness of the data set	0.993
No. of measured reflections	186465
No. of independent reflections	15884
No. of parameters	1269
No. of restraints	2272
S (all data)	0.994
$R(F)$ ($I \geq 2\sigma(I)$, all data)	0.0345, 0.0419
$wR(F^2)$ ($I \geq 2\sigma(I)$, all data)	0.0887, 0.0906
Extinction coefficient	0.00031(4)
$\Delta\rho_{max}$, $\Delta\rho_{min}$ / e·Å^{-3}	0.290, –0.326

13 References

1 S. Roy, H. Khatua, S. K. Das and B. Chattopadhyay, *Angew. Chem. Int. Ed.*, 2019, **58**, 11439–11443

2 P.S. Steinlandt, M. Hemming, X. Xie, S. I. Ivlev and E. Meggers, *Chem. Eur. J.* 2023, **29**, e202300267.

3 Y. Zhang, K. Shibatomi and H. Yamamoto, *Synlett*, 2005, 2837–2842.

4 A. Ageshina, G. A. Chesnokov, M. A. Topchiy, I. V. Alabugin, M. S. Nechaev and A. F. Asachenko, *Org. Biomol. Chem.*, 2019, **17**, 4523–4534.

5 A. Jančařík, J. Rybáček, K. Cocq, J. Vacek Chocholoušová, J. Vacek, R. Pohl, L. Bednárová, P. Fiedler, I. Císařová, I. G. Stará and I. Starý, *Angew. Chem. Int. Ed.*, 2013, **52**, 9970–9975.

6 V. Akhmetov, M. Feofanov, D. I. Sharapa and K. Amsharov, *J. Am. Chem. Soc*, 2021, **143**, 15420–15426.

7 M. Karras, J. Holec, L. Bednárová, R. Pohl, B. Schmidt, I. G. Stará and I. Starý, *J. Org. Chem.,* 2018, **83**, 5523–5538.

8 A. Bolje, D. Urankar and J. Košmrlj, *Eur. J. Org. Chem.*, 2014, 8167-8181.

9 X. Nie, C.-X. Ye, S. I. Ivlev and E. Meggers, *Angew. Chem. Int. Ed.*, 2022, **61**, e202211971.

10 A. Bolje and J. Košmrlj, *Org. Lett.*, 2013, **15**, 5084–5087.

11 F. Meloni, W. D. Brittain, L. Male, C. S. Le Duff, B. R. Buckley, A. G. Leach and J. S. Fossey, *Tetrahedron Chem*, 2022, **1**, 100004.

12 T. Li, C. Mou, P. Qi, X. Peng, S. Jiang, G. Hao, W. Xue, S. Yang, L. Hao, Y. R. Chi and Z. Jin, *Angew. Chem. Int. Ed*, 2021, **60**, 9362–9367.

13 Stefan Berger and Siegmar Braun, *200 and More NMR Experiments, a Practical Course*, Wiley-VCH, Weinheim, 2004.

14 F. Han, P. H. Choi, C.-X. Ye, Y. Grell, X. Xie, S. I. Ivlev, S. Chen and E. Meggers, *ACS Catal.* 2022, **12**, 10304-10312.

15 *X-Area 1.8.1*, STOE & Cie GmbH, Darmstadt, Germany, 2018.

16 *LANA-Laue Analyzer V 1.76.6*, STOE & Cie GmbH, Darmstadt, Germany, 2019.

17 *X-RED32 V 1.65*, STOE & Cie GmbH, Darmstadt, Germany, 2018.

18 G. M. Sheldrick, *Acta Crystallogr.* 2015, **A71**, 3–8.

19 G. M. Sheldrick, *Acta Crystallogr.* 2015, **C71**, 3–8.

20 C. B. Hübschle, G. M. Sheldrick, B. Dittrich, *J. Appl. Crystallogr.* 2011, **44**, 1281–1284.

21 D. Kratzert, I. Krossing, *J. Appl. Crystallogr.* 2018, **51**, 928–934.

22 A. L. Spek, *Acta Crystallogr., Sect. C: Struct. Chem.* 2015, **71**, 9–18

23 A. L. Spek, *PLATON - A Multipurpose Crystallographic Tool*, Utrecht University, Utrecht, The Netherlands, 2019.
